# High-intensity interval training versus progressive high-intensity circuit resistance training on endothelial function and cardiorespiratory fitness in heart failure: A preliminary randomized controlled trial

**DOI:** 10.1371/journal.pone.0257607

**Published:** 2021-10-01

**Authors:** Natália Turri-Silva, Amanda Vale-Lira, Kenneth Verboven, João Luiz Quaglioti Durigan, Dominique Hansen, Gerson Cipriano

**Affiliations:** 1 Health and Technologies in Health Sciences Program, University of Brasilia, Brasilia, Brazil; 2 BIOMED-REVAL (Rehabilitation Research Centre), Faculty of Rehabilitation Sciences, Hasselt University, Hasselt, Belgium; 3 Rehabilitation Sciences Program, Faculty of Physical Education, University of Brasília, Brasilia, Brazil; 4 Physical Therapy Department, University of Brasilia, Brasilia, Brazil; 5 Heart Centre Hasselt, Jessa Hospital, Hasselt, Belgium; Prince Sattam Bin Abdulaziz University, College of Applied Medical Sciences, SAUDI ARABIA

## Abstract

**Introduction:**

Exercise training is strongly recommended as a therapeutic approach to treat individuals with heart failure. High-intensity exercise training modalities still controversial in this population. The study aims to preliminary assess the consequences of high-intensity exercise training modalities, aerobic interval training (HIIT) and progressive high circuit-resistance training (CRT), on primarily endothelial function and cardiorespiratory fitness, and secondly on muscle strength and physical performance in heart failure patients.

**Methods:**

This preliminary multicentric randomized controlled trial comprised 23 heart failure patients, aged 56 ± 10 years old, mainly New York Heart Association classification I and II (%), hemodynamically stable, who compromise at least 36 exercise sessions of a randomly assigned intervention (HIIT, CRT or control group). Endothelial function, cardiopulmonary exercise testing, muscle strength and physical performance were completed at baseline and post-intervention.

**Results:**

Although no effects on endothelial function; both HIIT and CRT modalities were able to produce a positive effect on $\dot{V}O_{2}$ peak (HIIT = +2.1±6.5, CRT = +3.0±4.2 and control group = -0.1± 5.3 mL/kg/min, time*group p-value<0,05) and METs (HIIT = +0.6±1.8, CRT = +0.9±1.2 and control group = 0±1.6, time*group p-value<0,05). Only HIIT increased isokinetic torque peak (HIIT = +8.8±55.8, CRT = 0.0±60.7 and control group = 1.6±57.6 Nm) matched p-value<0,05. Regarding the physical performance, the CRT modality reduced chair stand test completion time (HIIT = -0.7±3.1, CRT = -3.3±3.2 and control group = -0.3±2.5 s, matched p-value<0,05 and HIIT improved global physical performance(time*group p<0,05).

**Conclusion:**

This preliminary study trends to indicate for the first time that high-intensity interval training promotes a jointly superior effect compared to progressive high intensity circuit-resistance training by improving cardiorespiratory fitness, muscular strength, and physical performance. Further research with larger cohort is necessary.

**Clinical trial registration number:**

ReBEC RBR-668c8v.

## Introduction

Cardiovascular disease is the most essential cause of premature mortality, causing over 17 million deaths worldwide [1]. More importantly, heart failure (HF) leads to significant reductions in quality of life and disease-free life years, and hence to elevations in healthcare expenditure (e.g., hospitalizations and medication) [2]. These reductions in quality of life can, at least in part, be explained by the exercise intolerance development [3]. Numerous pathophysiological mechanisms can lead to exercise intolerance, including skeletal muscle dysfunction and endothelial dysfunction [4].

Endothelial dysfunction also related to increased cardiovascular risk and the higher incidence of cardiovascular events in HF individuals [5]. Several mechanisms are involved in this endothelial dysfunction, including reduced production of nitric oxide [6], altered vessel thickness [7], and increased oxidative stress [8–10]. The vascular supply to skeletal muscles and alveoli is significantly reduced [11] and, thereby lowering the exercise capacity. Furthermore, peripheral adaptations such as sarcopenia, inflammation [12], and muscle strength reduction, alongside oxidative capacity reductions and decreased perfusion, are muscular modifications awaited in HF [13, 14], contributing to exercise intolerance [15].

Exercise intervention has been used as a clinically effective and safe treatment for HF (moderate GRADE quality of evidence for hospitalizations reduction and low for quality of life improvement) [16] to counteract endothelial and skeletal muscle dysfunction, [17–19]. Also, improvements in exercise capacity [20], cardiac function [21], and vascular flow [10, 11], induced by exercise training, are often observed.

Current guidelines recommend moderate-intensity exercise training for heart failure patients, 3 to 5 days/week, with a duration ranging up to 60 minutes [22]. However, some patients cannot sustain continuous aerobic exercise training at adequate intensity for prolonged duration, eliciting leg discomfort and/or dyspnea [23]. As a result, alternative and feasible exercise modalities should be explored. The aerobic interval training modality, for example, allows higher-intense bouts of shorter duration [24, 25] without losing patient adherence and good clinical effectiveness [26]. Moreover, resistance exercise recommendations for HF patients indicate intensities lower than 15 (out of 20) on the perceived exertion Borg scale, with a load ranging from 40 to 60% of one-repetition maximum test (1RM); however, the effect of progressive resistance training reaching higher loads in such population it is still necessary to explore since remains under intense debate [27]. In this sense, circuit-resistance training (CRT) might be considered an engaging alternative training modality.

Recent literature attributes to high-intensity interval training (HIIT), and CRT modalities positive effects in patients with HF [25, 28], as evidenced by a favourable effect on exercise capacity [25, 29, 30]. HIIT also improves peripheral O_2_ extraction in HF [13], while CRT improves skeletal muscle strength and energetic metabolism capacity through an increase in mitochondrial adenosine triphosphate (ATP) production [30]. However, what remains to be studied is the direct comparison of HIIT vs. CRT in HF patients on vascular function and exercise capacity which is this study objective. Considering that HIIT and CRT are training modalities characterized by shorter exercise bouts of greater intensity, with a specific focus/impact on skeletal muscle physiology, we hypothesized with this preliminary report that both exercise modalities promote improvement on vascular function and exercise capacity to a similar extend. If so, additional study could be initiate with large cohort with heart failure patients.

## Material and methods

### Trial design

**Fig 1:**
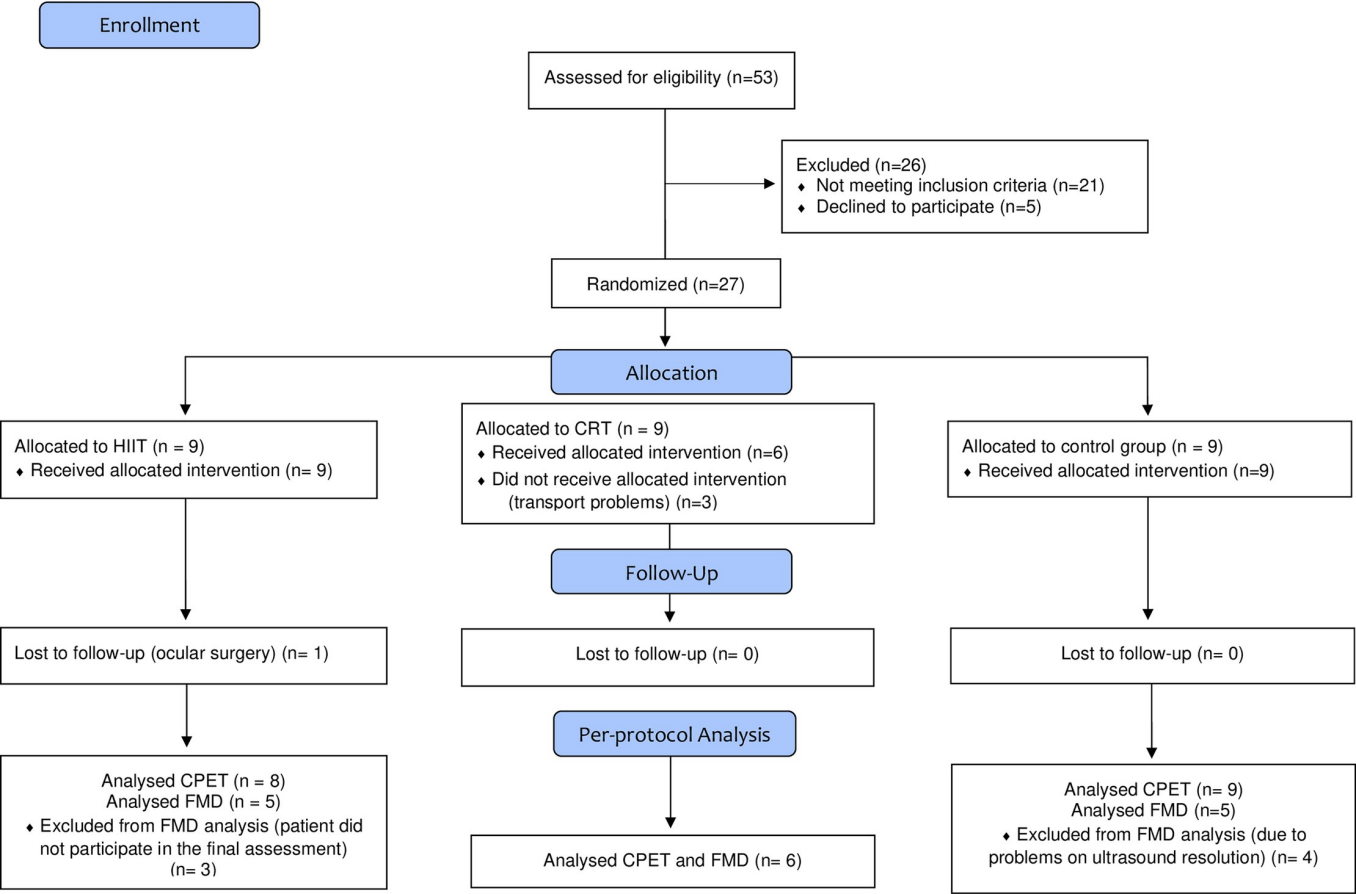
Study flowchart. HIIT, high-intensity interval training; CRT, circuit-resistance training; CPET, cardiopulmonary exercise testing; FMD, flow-mediated dilatation.

**Fig 2:**
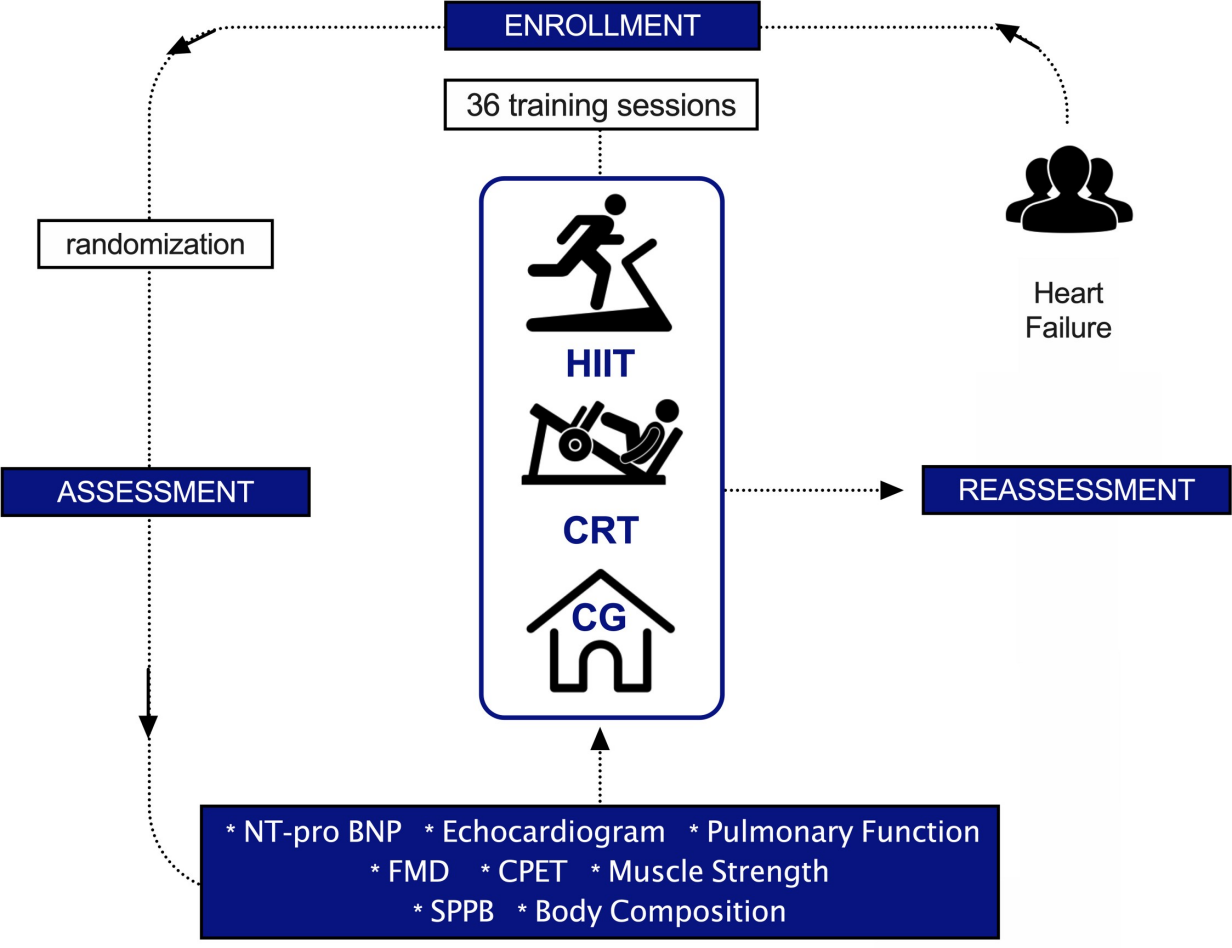
Study design. Study overview flowchart. HIIT, high-intensity interval training; CRT, circuit-resistance training; CG, control group; NT-pro BNP, N‐terminal pro‐brain natriuretic peptide; FMD, flow-mediated dilatation; CPET, cardiopulmonary exercise test; SPPB, Short Physical Performance Battery.

This preliminary randomized controlled trial is designed as a longitudinal, parallel, and quantitative study, following CONSORT recommendations (Consolidated Standards for Reporting) [31]. Patients were randomized into three groups: High-Intensity Interval Training (HIIT), Circuit-Resistance Training (CRT), and control (CG) with allocation ratio 1:1:1. Assessments occurred in two periods: at baseline and after 36 training sessions of intervention (reassessment) or 12 weeks of follow-up in the control group. One patient on the HIIT group did not complete all expect sessions due to ocular problem as indicated on CONSORT flow chart (Fig 1). Fig 2 shows the study design. CONSORT checklist can be found in the S1 File.

### Subjects and randomization

A total of 53 HF stable patients on optimal medical therapy were recruited for this randomized trial at University of Brasilia (Brazil). After exclusions, 27 patients were randomized and 23 completed the training protocol as indicated on CONSORT Flowchart (Fig 1). HF patients were included based upon HF diagnosis with reduced (HFrEF) and preserved ejection fraction (HFpEF) following 2016 ESC Guidelines [32], all referred by a cardiologist. Male and female individuals older than 35 years, not involved in an exercise training program within six months prior to the study, and without Chagas etiology diagnosis were included. Exclusion criteria were: smokers, active bacterial and viral infections, orthopedic symptoms/diseases that could limit exercise performance, and difficulties in reaching the exercise center three times per week. An independent researcher prepared the allocation of a random sequence (AVL), by sealed envelopes, to either HIIT, CRT, or CG groups. Blocks of 3 patients were always considered for randomization. The allocation concealment was maintained until starting intervention once it occurred after finishing all assessments, at least two weeks before starting the protocol. An independent researcher assigned participants to interventions (NTS). The study was performed in accordance with the standards set by the latest revision (2013) of the Declaration of Helsinki and approved by the local ethical committee (University of Brasilia, Brasilia, Brazil). All participating patients gave written informed consent after careful explanation about the nature and risks of the study’s experimental procedures (registration number RBR-668c8v).

### Clinical measures

#### Patient’s characteristics

Based on the medical anamnesis and clinical examination, the prevalence of positive cardiovascular risk factors, surgery procedures, and medication prescription were collected. The NYHA (New York Heart Association) and Weber clinical assessment [33], body weight, height, and BMI were obtained. A venous blood sample at a fed state was kept at heparinized (sodium) tubes ^a^ (Vacuette^®^) at room temperature and analyzed within 5 hours. The manufacturer’s control limits of acceptability were followed. N‐terminal pro‐brain natriuretic peptide (NT‐proBNP) was measured by using test strips (CARDIAC proBNP+) containing monoclonal and polyclonal antibodies against epitopes of the NT-proBNP molecule on a point-of-care device Cobas h232 ^b^ (Roche Diagnostics). Moreover, the left ventricle ejection fraction (LVEF) was measured by ultrasound for echocardiography ^c^ (Philips ATL) in semi-lateral decubitus position (by Simpson method, LVEF, %) by using a 4–2 MHz transducer equipped with a second harmonic image through digital Imaging and communication in medicine format and followed recommendations of the American Society of Echocardiography and the European Association of Cardiovascular Imaging [30, 31]. Pulmonary Function parameters [maximum inspiratory pressure (MIP, cmH_2_O); forced volume expiratory in 1 second (FVE_1_, L/s); forced vital capacity (FVC, L); and FVE_1_/FVC ratio, % were assessed by a spirometry ^d^ test (MicroLab) following standard recommendations of the European Respiratory Society (ERS) [34].

#### Deviations from the initial study protocol

Changes were made in the initial protocol in order to adapt the trial to a multicentric one. More informations about the study protocol can be found on S2 and S3 Files. The outcomes initially cited on Brazilian Ethical Committe “quality of life” and “oxygen extraction” were not included on Belgic ethical Commite due to technical limitations. In addition, the outcomes “autonomic modulation”, “muscle ultrasound”, “pulmonar function” and "oxygen extraction" were not included on this paper, but they were obtained and will be presented in a future paper.

### Treatment outcomes

All outcomes were evaluated before and after finishing the protocol period.

#### Primary outcomes

*Endothelial function*. Prior to evaluating endothelial function [35], subjects received prior instructions, which included a light meal within two hours before testing and abstain from caffeine, alcohol, and exercise for 24 h prior to testing. Tests were performed in the same period of the day to avoid any circadian effect, and the temperature was set at 24° C. Subjects preparation followed standard recommendations [36]. A cuff was attached to the subjects’ right arm, who remained at rest supine position for 5 min. The diameter was measured for two minutes using high-resolution Doppler duplex ultrasound ^e^ equipment in Brazil (Phillips) according to Thijssen et al. 2011 [37]. For this purpose, a 9 MHz linear matrix transducer was positioned over the brachial artery discretely proximal to the cubital fossa. Signals were obtained in duplex mode at a pulse frequency of 5 MHz and corrected by an insonation angle of 60°. Sample volumes were adjusted to encompass the entire vessel’s lumen without extending beyond the walls, and the slider adjusted in the middle of the vessel. The brachial artery’s flow-mediated dilation (FMD) was evaluated by a single examiner on the right arm in the supine position, as previously described [38, 39]. After resting period evaluation, the cuff was inflated to a pressure of 220 mmHg and held for 5 minutes. The measurements were recorded continuously for 3 min after rapid cuff deflation. All vascular variables were obtained by using specialized edge-detection software ^f^ (Cardiovascular Suite). Percentage variation of FMD was normalized [40]. Baseline arterial diameter (mm), peak arterial diameter (mm), FMD (%)–formula (peak diameter—baseline diameter) / (baseline diameter) * 100—and delta variations were considered for analysis [36, 41]. Images with inadequate resolution were excluded from the analysis. The physician who made the data acquisition was blinded to allocation for pre measurements, but not for post measurements.

*Cardiorespiratory fitness*. Cardiorespiratory fitness was obtained by a maximal cardiopulmonary exercise testing (CPET) on a cycle ergometer ^g^ (Corival) through an incremental symptom-limited test on a 0-watt electro-magnetic under the supervision of a cardiologist blinded to allocation. Room temperature was controlled (24° C). A ramp protocol started with an initial workload (warm up) of 0 W (free wheel) follow by an individual incremental protocol with a workload ranging from 10 to 20 Watt per minute according to clinical assessment based on NYHA class [42]. Pre and post tests were performed by using the same workload. The subjects were instructed to wear comfortable clothes without movement restriction, and the same prior instructions from the endothelial procedure. A breath-by-breath gas analyzer ^h^ was used to measure oxygen uptake, carbon dioxide production, and ventilation (CPET, Cosmed, Rome, Italy), while the heart rate and electrocardiogram ^i^ (Quark 3T12x, Cosmed, Rome, Italy) were measured continuously. All patients cycled until volitional exhaustion within a fatigue-limited exercise duration of 8 to 12 minutes [43]. Verbal encouragement was given to the patients on each minute and when approaching to the end of the test in order to maintain the requested RPM ≥55. The test was ended when patients were no longer able to maintain a cycling frequency of ≥55 rpm. Peak exercise effort was confirmed when the respiratory gas exchange ratio (RER) was ≥1.10, in combination with exertional dyspnoea, leg, and/or general fatigue. The first and second ventilatory thresholds (VT1 and VT2, respectively) from CPET were considered for exercise prescription. Outcome measures also included exercise duration (s), cycling power output (watt), peak respiratory exchange ratio (RER peak), heart rate at the first ventilatory threshold (HR VT1, bpm), heart rate at the second ventilatory threshold (HR VT2, bpm), peak heart rate (HR peak, bpm), oxygen uptake at the first ventilatory threshold ($\dot{V}O_{2}$ VT1, mL/kg/min), oxygen consumption and at the second ventilatory threshold ($\dot{V}O_{2}$ VT2, mL/kg/min), peak oxygen uptake ($\dot{V}O_{2}$ peak, mL/kg/min and mL/min) (30 s average [42], maximal metabolic equivalent (METs max) [44].

The first ventilatory threshold (VT1) was determined using the V-slope method, and this threshold was double-checked by establishing the nadir of the VE/VO_2_ versus work rate relationship. The VT1 marks the limit between the light-to-moderate and the moderate-to-high intensity effort domains. Next, the second ventilatory threshold (VT2) was determined, using the VE vs. VCO_2_ plot, on the point where VE increases out of proportion to VCO_2_, and this threshold was double-checked by establishing the nadir of the VE/VCO_2_ versus W relationship. The VT2 is related to the critical power, which is the upper intensity limit for prolonged aerobic exercise.

#### Secondary outcomes

*Muscle strength*. Muscle strength was obtained by isokinetic test on an isokinetic dynamometer ^j^ (Biodex System 3 PRO, Medical Inc., New York, EUA). Patients adopted a seated position (90° hip) in a good posture. Belts were used to stabilize the thigh, pelvis, and trunk. Legs alignment was verified.

All patient measures and dynamometer position (as chair height, chair base, seat backrest, dynamometer distance, arms distance) were standardized at baseline and follow-up evaluation. Patients started isokinetic familiarization by performing six submaximal repetitions as fast as possible. After 3 minutes of recovery, the endurance protocol started, which consisted of 20 maximal repetitions at 180°/s, once [45]. Peak torque (N-M); peak torque and body weight ratio (%); total repetition maximum work (J); total work (J); work fatigue (%) and average power (W) were obtained.

*Physical performance*. The physical performance was assessed through the Short Physical Performance Battery (SPPB) test. SPPB execution followed previous recommendations [46] to test the balance, lower limb strength, and walking velocity. The total score of SPPB results from the sum of balance, walking speed, and lower limb strength, totalizing 12 points. Scores from 0 to 3 points were considered incapacity or very poor; 4 to 6 low capacity; 7 to 9 moderate and 10 to 12 good performance.

*Body composition*. The whole-body composition was estimated by dual x-ray absorptiometry scan ^k^ (GE Healthcare) [47, 48]. Total body fat mass and total body lean mass were expressed in absolute values and percentages.

#### Intervention

**Fig 3:**
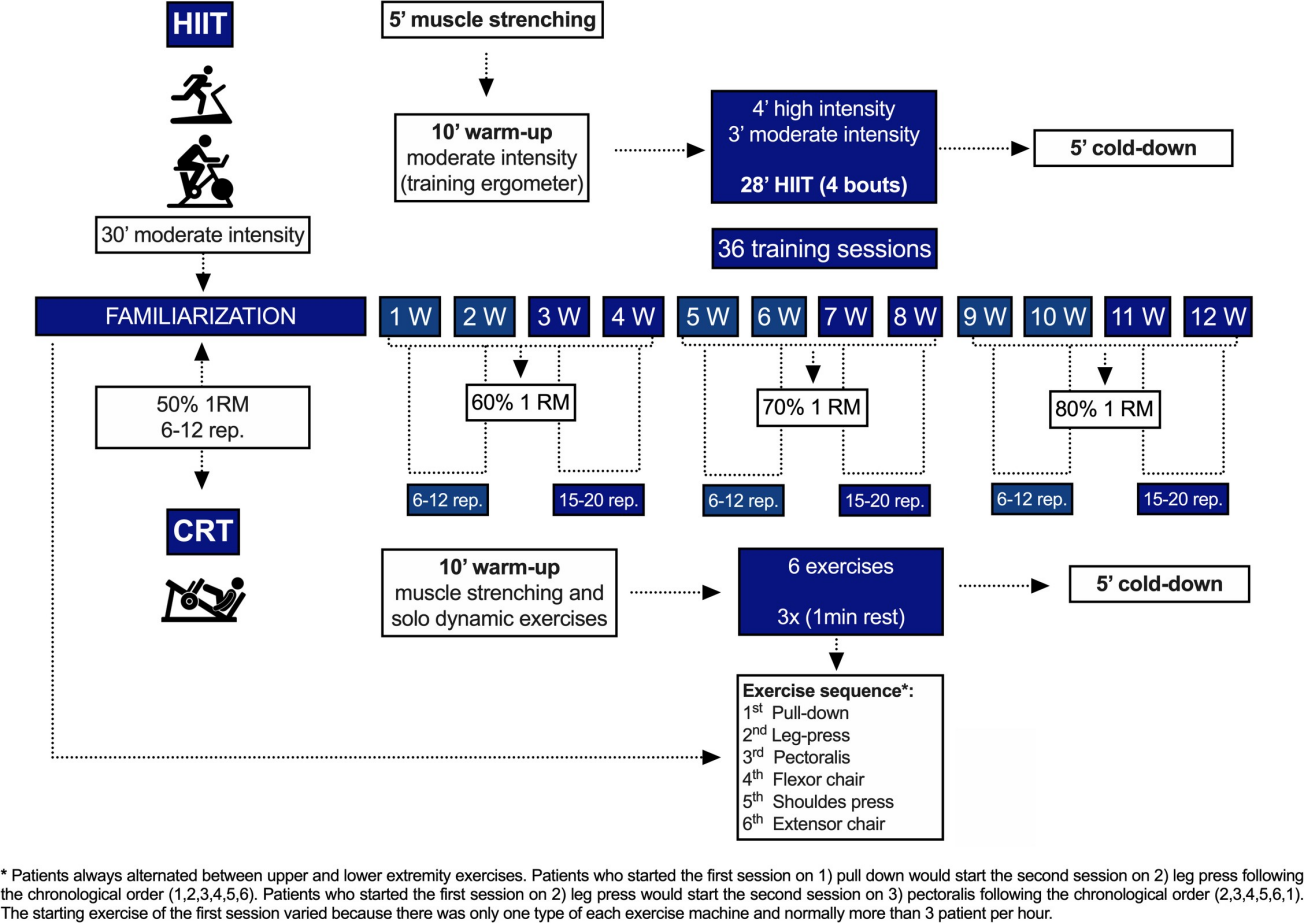
Training protocol. Design of the training protocol. HIIT, high-intensity interval training; CRT, circuit-resistance training; 1RM, one-repetition maximum; W, weeks; rep, repetitions; min, minute.

*Exercise training protocols*. The exercise sessionsoccurred 3 times per week along 36 sessions with a matched session duration (approximately 50 minutes). Training familiarization for both modalities was established for the patient’s adaptation (6 to 10 sessions) according to the patients performance to be able to reach HIIT prescription. HR monitorization was continuously registered by Polar^® l^ (RS800) during training sessions for clinic monitorization control. The exercise training protocol design is illustrated on Fig 3.

*Safety*, *tolerability and adherence*. Trained physiotherapists undertook both training protocols with the supervision of a cardiologist who was available in case of health concerns. Before and after finishing the training sessions, blood pressure, heart rate, and perceived exertion rate were assessed. Also, clinical signs, such as excessive tiredness, intense sweating, paleness, palpitations, and chest pain, were always investigated. There were no medical complications during the training sessions. In each session the physiotherapists recorded whether or not the patients from the HIIT group reached the expected heart rate according to the training zones. Information about patient adherence was always registered in each session.

*Circuit-Resistance Training (CRT)*. CRT exercises were executed to target large muscle groups, including: 1) pull-down, 2) leg press, 3) pectoralis, 4) flexor chair, 5) shoulder press, and 6) extensor chair machines. Strength measurements were estimated by a one-repetition maximum test (1RM) [49]. The CRT session was preceded by a 10-minute warm-up session led by a physiotherapist, consisting 5 minutes of muscle stretching and 5 minutes of dynamic movements. CRT was performed in resistive stations ^m^ (EN-Dynamic). The order of the exercise sequence was based on the exercise machines availability at the gymnasium, which were numbered as 1) pull-down, 2) leg press, 3) pectoralis, 4) flexor chair, 5) shoulder press, and 6) extensor chair machines. Patients always alternated between upper and lower extremity exercises. Patients who started the first session on 1) pull down would start the second session on 2) leg press following the chronological order (1,2,3,4,5,6). Patients who started the first session on 2) leg press would start the second session on 3) pectoralis following the chronological order (2,3,4,5,6,1). The starting exercise of the first session varied because there was only one type of each exercise machine and normally more than 3 patient per hour. During the 6 sessions of familiarization on CRT, the training load started at 50% of 1RM, including 3 circuit series of 12 repetitions. After finishing the familiarization, the workloads were set at 60% 1RM for the 1^st^ month, 70% 1RM for the 2^nd^ month, and 80% 1RM for the 3^rd^ month [57]. Repetitions varied from 6 to 12 in the first two weeks of each month and from 15 to 20 in the last two weeks. 3 circuit series of each exercise with 1 min of rest between exercises were executed. Exercise cadence ratio was 1:2. Exercise training protocol design is demonstrated on Fig 3.

*High-Intensity Interval Training (HIIT)*. HIIT was performed on a treadmill ^n^ (Biodex Medical Systems) and ergometric bicycle ^o^ (Biodex Medical Systems) alternately at each session. During the familiarization period, patients performed moderate-intensity continuous aerobic training for 30 minutes. HIIT was gradually incorporated during familiarization in such a way as to guarantee adequate HR response during the research protocol. In each HIIT session, individuals initially performed 10 minutes of continuous aerobic training at moderate intensity which was established according to previous CPET by considering a heart rate range 5% below (lower limit of target heart rate) and 5% above (upper limit of target heart rate) of the first ventilatory threshold (VT1), followed by a 28 minutes of HIIT, now at light- and high-intensity loads (i.e., light intensity–defined as a heart rate range between the moderate-intensity lower limit of target heart rate and 10% below; and high intensity—defined as a heart rate range between moderate-intensity upper limit of target heart rate and 10% above); as recommended by the joint position statement of the European Association for Cardiovascular Prevention and Rehabilitation, the American Association of Cardiovascular and Pulmonary Rehabilitation and the Canadian Association of Cardiac Rehabilitation and by the latest position statement from the Secondary Prevention and Rehabilitation Section of the European Association of Preventive Cardiology which is in press [50, 51] HIIT protocol periods was characterized by 3 minutes of exercise at high intensity followed by 4 minutes of exercise at moderate intensity, totalizing 4 cycles of 7 minutes, resulting in 28 minutes of HIIT [52].

The loads adjustment for HIIT started always by following increments on velocity, however when patient could not tolerate higher speed the load was adjusted by increments on inclination. Regardless the type of increment, patient should always reach the desirable heart rate according to the expected intensity.

Exercise training protocol design is demonstrated on Fig 3.

*Control group*. Patients from the control group were instructed to keep their routine without changing habits during the research period.

### Statistical analysis

Descriptive analyses were used for sample characterization of the primary and secondary outcomes. Shapiro-Wilk test was applied to check data normality, and parametric or non-parametric tests were applied accordingly. A mixed ANOVA with post-hoc Tukey was used for parametric distribution for all outcomes considering factors group (between) and time (within) to compare HIIT, CRT, and control group. The Friedman two-way test for non-parametric distribution following within non-parametric comparisons for the outcome SPPB. Delta comparisons were performed by following one-way ANOVA (parametric distribution) or Kruskal-Wallis (non-parametric distribution) for the outcome FMD. Comparison between pre and post moments at the same group was made by following the paired student T-test (parametric distribution) or Wilcoxon test (non-parametric distribution) for all outcomes. Sample size calculation was made by using the G*Power Software 3 ^p^ (Heinrich Heine Universität Düsseldorf), selecting an F test with the statistical test ANOVA (fixed effects, special, main effects and interactions), following a priori test. The total sample size was based on a pilot study (three patients in each group), calculated from the partial eta square (mixed ANOVA, between groups) for the primary outcomes $\dot{V}O_{2}$ peak, mL/kg/min (eta partial squared 0.19; effect size 0.48; total sample size 45–15 patients per group) and FMD, % (eta partial squared 0.23; effect size 0.55; total sample size 36–12 patients per group). The alpha error considered was 0.05 and power 1-beta was 0.80. Considering this is a stop early trial due to covid outbreak, the total sample size was not reached, but the preliminary findings are expressed. Statistical software SPSS version 22.0 ^q^ and GraphPad Prism (8.4.0 version) ^r^ were used for statistical analysis and figure production. P-value followed a 5% significance level.

## Results

Recruitment and follow up occurred from 2018 and 2019. Patients’ characteristics are presented in Table 1. The trial was stopped early due to covid outbreak. At baseline, patients in all groups were similar in terms of sex, age, body mass index, functional classification (NYHA and Weber classifications), heart failure classification (reduced, mid-range, or preserved ejection fraction), cardiorespiratory fitness (as represented by $\dot{V}O_{2}$ peak, mL/kg/min and mL/min, and cycling peak power output, Watt), left ventricular ejection fraction (%) and pulmonary function (p > 0.05).

**Table 1:** Sample characterization.

	HIIT (n = 8)	CRT (n = 6)	CG (n = 8)	p value
	Baseline	Baseline	Baseline	
Male (n, %)	5 (62.5)	4 (66.7)	7 (87.5)	0.590^a^
Age (years)	60.9 ± 9.7	55.0 ± 10.9	56.0 ± 9.7	0.497^b^
BMI (kg/m^2^)	29.4 ± 5.2	32.9 ± 6.5	28.6 ± 4.5	0.706^b^
***Classification***				
NYHA I/II (n, %)	3 (37.5) / 3 (37.5)	2 (33.3) / 3 (50.0)	5 (62.5) / 2 (25.0)	0.831^a^
NYHA III (n, %)	2 (25.0)	1 (16.7)	1 (12.5)	
Weber Class I/II (n, %)	3 (37.5) / 1 (12.5)	1 (16.7) / 3 (50.0)	5 (50.0) / 3 (37.5)	0.421^a^
Weber Class III (n, %)	4 (50.0)	2 (33.3)	1 (12.5)	
HFrEF (n, %)	3 (37.5)	3 (50.0)	2 (25.0)	0.273^a^
HFmEF (n, %)	0 (0.0)	2 (33.3)	3 (37.5)	
HFpEF (n, %)	5 (62.5)	1 (16.7)	3 (33.3)	
***Cardiorespiratory fitness***				
$\dot{V}O_{2}$ peak (mL/kg/min)	17.5 ± 4.2	16.9 ± 2.5	20.2 ± 3.3	0.186^b^
$\dot{V}O_{2}$ peak (mL/min)	1437.3 ± 411.3	1565.7 ± 365.6	1605.0 ± 458.4	0.712^b^
Cycling peak power output (Watt)	96.8 ± 26.3	104.2 ± 27.7	123.3 ± 35.2	0.225^b^
***Echocardiogram***				
LVEF *Simpson* (%)	50.4 ± 17.0	42.2 ± 13.5	46.8 ± 14.4	0.614^b^
***Pulmonary Function***				
MIP (cmH_2_O)	83.8 ± 38.5	83.0 ± 34.5	103.5 ± 28.5	0.511^b^
FVE_1_ (L/s)	2.3 ± 0.7	2.3 ± 0.9	2.5 ± 0.8	0.888^b^
FVC (L)	3.2 ± 0.9	3.0 ± 1.1	3.2 ± 0.8	0.904^b^
FVE_1_/FVC (%)	70.4 ± 8.2	74.5 ± 7.6	76.0 ± 11.4	0.522^c^
***Etiology for HF***				
Ischaemic (n, %)	5 (62.5)	4 (66.7)	7 (87.5)	0.566^a^
Hypertension (n, %)	1 (12.5)	1 (16.7)	0 (0.0)	
Idiophatic (n, %)	2 (25.0)	1 (16.7)	1 (12.5)	
Coronary Artery Disease (n, %)	4 (50.0)	4 (66.7)	7 (87.5)	0.279^a^
***Risk Factors***				
Arterial Hypertension (n, %)	4 (50.0)	4 (66.7)	4 (50.0)	0.758^a^
Diabetes Mellitus (n, %)	3 (37.5)	3 (50.0)	2 (25.0)	0.853^a^
Dyslipidaemia (n, %)	5 (62.5)	5 (83.3)	6 (75.0)	0.842^a^
Obesity (n, %)	4 (50.0)	4 (66.7)	3 (37.5)	0.520^a^
***Drugs***				
Beta-blocker (n, %)	7 (87.5)	5 (83.3)	8 (100.0)	0.723^a^
ACE-inhibitors (n, %)	4 (50.0)	4 (66.7)	7 (87.5)	0.279^a^
Angiotensin Receptor Blockers (n, %)	1 (12.5)	1 (16.7)	2 (25.0)	1.000^a^
Loop Diuretics (n, %)	6 (75.0)	5 (83.3)	4 (50.0)	0.522^a^
Antiplatelet (n, %)	3 (37.5)	5 (83.3)	3 (37.5)	0.216^a^
Statins (n, %)	4 (50.0)	5 (83.3)	7 (87.5)	0.290^a^
Coronary Vasodilators (n, %)	2 (25.0)	0 (0.0)	1 (12.5)	0.751^a^
Antacids (n, %)	0 (0.0)	1 (16.7)	2 (25.0)	0.460^a^
Antiarrhythmic (n, %)	1 (12.5)	0 (0.0)	2 (25.0)	0.751^a^
Antidiabetic (n, %)	2 (25.0)	2 (33.3)	2 (25.0)	1.000^a^
Anticoagulants (n, %)	1 (12.5)	0 (0.0)	2 (25.0)	0.751^a^

Values are expressed as mean ± standard deviation (SD) and frequencies (%). HIIT, high-intensity interval training; CRT, circuit-resistance training; CG, control group; BMI, Body Mass Index; kg, kilogram; m^2^, squared meter; NYHA, New York Heart Association Functional Classification); HFrEF, heart failure reduced ejection fraction; HFmEF, heart failure mid-range ejection fraction; HFpEF, heart failure preserved ejection fraction; $\dot{V}O_{2}$ peak, oxygen consumption uptake; mL, mililiter; min, minute; LVEF, left ventricular ejection fraction; MIP, maximum inspiratory pressure; cmH_2_O, centimeters of water; FVE_1_, forced expiratory volume in first second; L/s, liters per second; FVC, forced vital capacity; L, liters; FVE_1_/FVC, ratio between forced expiratory volume in first second and forced vital capacity; ICD, implantable cardioverter-defibrillator; CP, cardiac pacemaker; ACE, angiotensin-converting enzyme.

^a^ Fisher’s Exact Test.

^b^ One-way ANOVA.

^c^ Kruskal-Wallis H Test.

*p ≤0.05.

### Safety, tolerability and adherence

**Fig 4:**
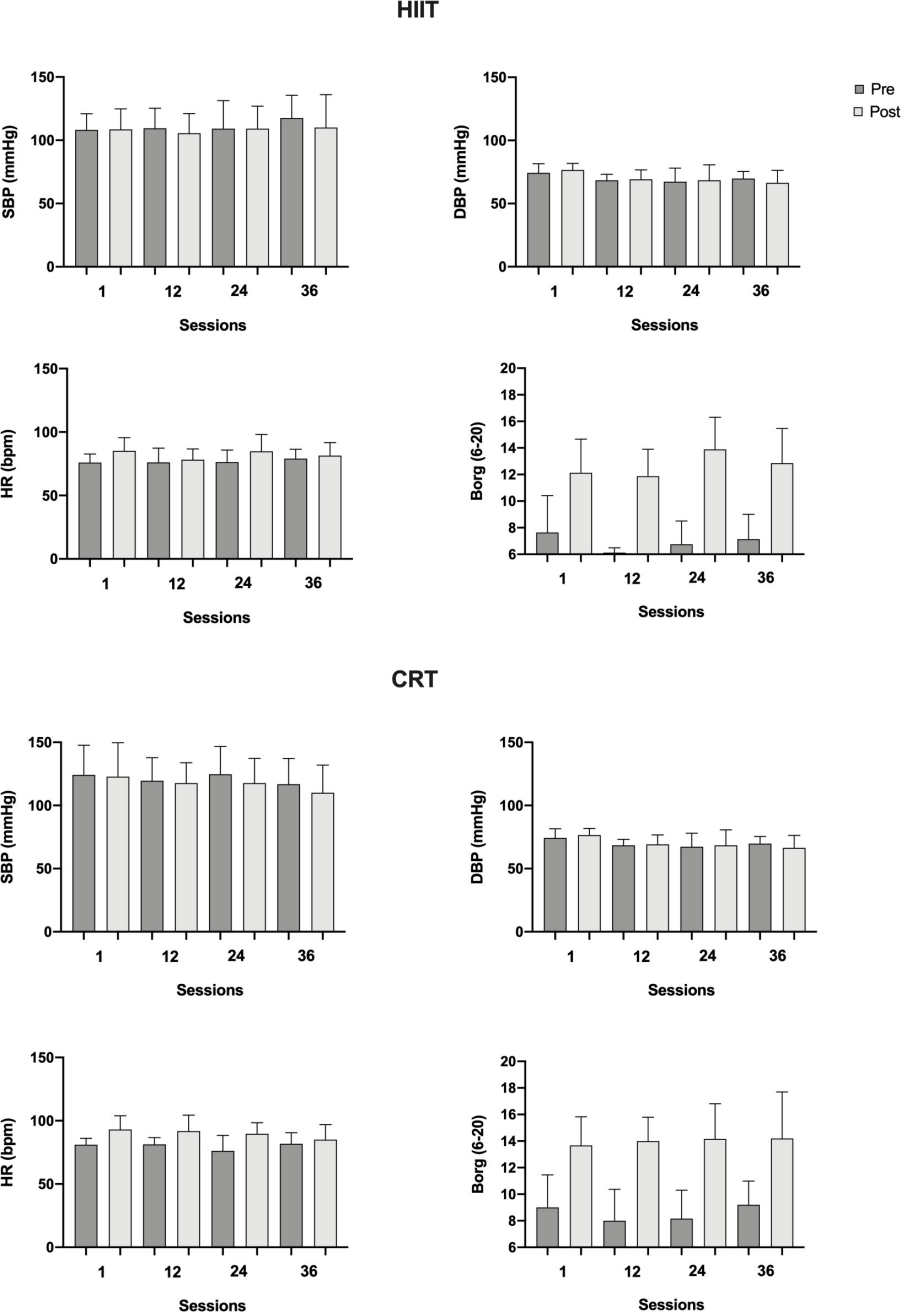
Clinical measurements at training sessions. HIIT, high-intensity interval training; CRT, circuit-resistance training; CG, control group; SBP, systolic blood pressure; mmHg, milimeters of mercury; DBP, diastolic blood pressure; HR, heart rate; bpm, beats per minute; Borg (6–20), perceived of exertion.

For safety and exercise prescription reasons, heart rate were always clinically monitored by using wearable heart rate monitors in both training groups. Also, blood pressure was measured before and after an exercise session. Throughout the intervention period of CRT occurred the following events: one patient presented hypotension, one diabetic patient presented three peaks of hyperglycemia on different days, one patient presented hypoglycemia at one session, and one patient referred higher dyspnea during the first three sessions of intervention. All events were controlled. For HIIT modality, the following events occurred: one patient did not reach the anaerobic threshold at two different sessions, and another patient-reported angina at the end of session thirty-two. The patient who reported angina was followed to the medical doctor, who did not detect any problem that could affect exercise training. Borg scores indicated good tolerability, not reaching mean values higher than 15 (Borg scale 6–20) in any intervention group, indicating good tolerability. In both HIIT and CRT, adherence to the exercise sessions was high (93.51% and 97.22% of sessions performed, respectively). Fig 4 demonstrates the clinical measurements of the parameters assessed before starting and at the end of the training sessions number 1, 12, 24 and 36.

### Endothelial function

Table 2 illustrates the endothelial function before and after training and in the control group. Patients were equally distributed at baseline, meaning there was no differences between groups at the baseline moment (p>0,05). No changes over time or between groups were found (p>0.05). To conclude, HIIT nor CRT was potent to improve vascular function.

**Table 2:** Impact of interventions on endothelial function in heart failure.

FMD	HIIT		CRT		CG		Within-group difference (post minus pre)			Between-group difference		
	Mean ± SD (95% CI)		Mean ± SD (95% CI)		Mean ± SD (95% CI)		MD (95% CI)			MD (95% CI)		
	pre (n = 5)	post (n = 5)	pre (n = 6)	post (n = 6)	pre (n = 4)	post (n = 4)	Δ HIIT	Δ CRT	Δ CG	HIIT vs. CRT	HIIT vs. CG	CRT vs. CG
Rest diameter (mm)	4.54 ± 1.09 (3.19 to 5.88)	4.54 ± 0.92 (3.39 to 5.68)	4.38 ± 0.58 (3.77 to 4.98)	4.52 ± 0.61 (3.88 to 5.17)	4.36 ± 1.17 (2.94 to 6.23)	4.35 ± 0.51 (3.55 to 5.16)	0.00 (-0.67 to 0.67)	0.15 (-0.47 to 0.76)	-0.01 (-0.76 to 0.74)	0.09 (-1.24 to 1.41)	0.18 (-1.29 to 1.65)	0.09 (-1.32 to 1.50)
Peak diameter (mm)	4.77 ± 1.10 (3.41 to 6.13)	4.75 ± 0.88 (3.66 to 5.84)	4.57 ± 0.59 (3.95 to 5.19)	4.74 ± 0.57 (4.14 to 5.33)	4.54 ± 1.12 (2.77 to 6.31)	4.58 ± 0.39 (3.96 to 5.21)	-0.02 (-0.72 to 0.68)	0.17 (-0.47 to 0.80)	0.04 (-0.74 to 0.82)	0.10 (-1.16 to 1.37)	0.19 (-1.21 to 1.60)	0.09 (-1.26 to 1.44)
FMD (%)	5.37 ± 2.59 (2.16 to 8.59)	5.01 ± 3.93 (0.13 to 9.90)	4.54 ± 2.65 (1.76 to 7.32)	4.96 ± 4.54 (0.19 to 9.72)	4.62 ± 2.60 (0.48 to 8.76)	5.51 ± 3.61 (-0.22 to 11.25)	-0.36 (-5.05 to 4.33)	0.42 (-3.86 to 4.70)	0.90 (-4.35 to 6.14)	0.45 (-4.42 to 5.31)	0.13 (-5.27 to 5.52)	-0.32 (-5.51 to 4.87)

Values are expressed as mean ± standard deviation (SD), mean difference (MD) and 95% CI (confidence interval). Comparisons between groups were analyzed by the mixed ANOVA with Bonferroni post-test (within and between) for all parameters.

*p ≤0.05. HIIT, high-intensity interval training; CRT, circuit-resistance training; CG, control group; FMD, flow-mediated dilatation; mm, millimeter; Δ, post—pre values.

As, S1 Fig illustrates the individual dynamic of flow-mediated dilation per group.

### Cardiorespiratory fitness

Table 3 illustrates the effects of the interventions on cardiorespiratory fitness. Patients were equally distributed in relation to all CPET parameters at baseline (p>0,05). Differences were found among groups for $\dot{V}O_{2}$ peak (mL/.kg/min) and METs max in which patients from HIIT experienced significant improvements (p<0.05), next to improvements in cycling power output (p = 0.019). As a result of CRT, improvements also occurred for $\dot{V}O_{2}$ peak (mL/kg/min) (p = 0.026) and METs max (p-values of 0.025).

**Table 3:** Impact of interventions on exercise capacity in heart failure patients.

CPET	HIIT		CRT		CG		Within-group difference (post minus pre)			Between-group difference		
	Mean ± SD (95% CI)		Mean ± SD (95% CI)		Mean ± SD (95% CI)		MD (95% CI)			MD (95% CI)		
	pre (n = 8)	post (n = 8)	pre (n = 6)	post (n = 6)	pre (n = 8)	post (n = 8)	Δ HIIT	Δ CRT	Δ CG	HIIT vs. CRT	HIIT vs. CG	CRT vs. CG
Time Exercise (min)	9.2 ± 2.6 (7.0 to 11.4)	10.6 ± 2.4 (8.5 to 12.6)	10.0 ± 2.8 (7.0 to 12.9)	10.8 ± 2.1 (8.6 to 13.0)	10.2 ± 2.6 (8.1 to 12.4)	10.4 ± 2.6 (8.2 to 12.5)	-1.3 (-0.3 to 3.0)	0.8 (-1.1 to 2.7)	0.1 (-1.5 to 1.8)	0.5 (-2.9 to 3.9)	0.4 (-2.7 to 3.5)	-0.1 (-3.5 to 3.3)
Cycling peak power output (watt)	**96.8 ± 26.3** (74.8 to 118.7)	**114.9 ± 34.7** (85.9 to 143.9)	104.2 ± 27.7 (75.1 to 133.3)	112.0 ± 34.1 (76.2 to 147.8)	123.3 ± 35.2 (93.8 to 152.7)	123.6 ± 37.5 (99.3 to 155.0)	18.1 (4.4 to 31.9)*	7.8 (-8.1 to 23.7)	0.4 (-13.4 to 14.1)	2.3 (-43.4 to 47.9)	17.6 (-24.6 to 59.9)	15.4 (-30.3 to 61.0)
RER peak	1.2 ± 0.1 (1.1 to 1.3)	1.2 ± 0.1 (1.1 to 1.2)	1.2 ± 0.1 (1.0 to 1.3)	1.1 ± 0.1 (1.0 to 1.2)	1.2 ± 0.1 (1.1 to 1.3)	1.2 ± 0.1 (1.2 to 1.3)	-0.1 (-0.2 to 0.1)	-0.1 (-0.2 to 0.1)	0.0 (-0.2 to 0.1)	-0.1 (-0.2 to 0.1)	0.0 (-0.1 to 0.1)	0.1 (-0.1 to 0.2)
HR VT1 (bpm)	100.3 ± 13.0 (89.4 to 111.1)	100.4 ± 11.0 (91.2 to 109.6)	109.2 ± 12.8 (93.4 to 125.0)	107.6 ± 8.5 (97.1 to 118.1)	101.4 ± 20.5 (84.3 to 118.5)	96.3 ± 15.3 (83.5 to 109.0)	0.1 (-13.5 to 13.8)	-1.6 (-18.8 to 15.6)	-5.1 (-18.8 to 8.5)	8.1 (-10.7 to 26.9)	-1.5 (-18.0 to 15.0)	-9.6 (-28.4 to 9.2)
HR VT2 (bpm)	113.1 ± 19.0 (97.3 to 129.0)	122.4 ± 13.2 (110.2 to 134.7)	119.3 ± 16.4 (102.1 to 136.6)	123.2 ± 5.6 (116.1 to 130.1)	122.8 ± 20.8 (105.3 to 140.2)	116.4 ± 12.7 (105.8 to 127.0)	7.6 (-7.8 to 23.0)	2.0 (-16.1 to 20.2)	-6.4 (-21.0 to 8.3)	3.4 (-17.0 to 23.8)	2.6 (-16.0 to 21.3)	-0.8 (-21.0 to 19.4)
HR peak (bpm)	125.0 ± 24.7 (104.3 to 145.7)	129.4 ± 20.5 (112.2 to 146.6)	122.7 ± 16.9 (104.9 to 140.4)	129.2 ± 9.9 (118.8 to 139.5)	142.0 ± 17.9 (127.0 to 157.0)	139.9 ± 14.5 (128.8 to 152.0)	4.4 (-13.4 to 22.1)	6.5 (-14.0 to 27.0)	-2.1 (-19.9 to 15.6)	-1.3 (-23.5 to 21.0)	13.8 (-6.9 to 34.4)	15.0 (-7.3 to 37.3)
$\dot{V}O_{2}$ VT1 (mL/kg/min)	11.0 ± 2.2 (9.2 to 12.8)	13.8 ± 1.9 (12.2 to 15.4)	13.8 ± 3.2 (10.4 to 17.2)	15.0 ± 2.9 (12.0 to 18.1)	13.5 ± 6.4 (8.1 to 18.9)	13.1 ± 3.0 (10.6 to 15.6)	2.7 (-1.6 to 7.1)	1.3 (-3.8 to 6.3)	-0.4 (-4.8 to 4.0)	2.0 (-2.0 to 6.0)	0.9 (-2.8 to 4.6)	-1.1 (-5.1 to 2.9)
$\dot{V}O_{2}$ VT2 (mL/kg/min)	**15.1 ± 2.5** (13.1 to 17.1)	**18.8 ± 3.4** (15.6 to 21.9)	17.9 ± 5.0 (12.7 to 23.2)	19.2 ± 2.6 (16.5 to 21.9)	17.4 ± 2.0 (15.7 to 19.0)	16.2 ± 2.3 (14.2 to 18.1)	3.7 (1.0 to 6.3)*	1.3 (-1.6 to 4.2)	-1.2 (-3.7 to 1.3)	1.6 (-2.2 to 5.4)	-0.2 (-3.7 to 3.1)	-1.8 (-5.6 to 2.0)
$\dot{V}O_{2}$ peak (mL/kg/min)	**17.5 ± 4.2** (14.0 to 21.0)	**19.6 ± 4.9** (15.6 to 23.7)	**16.9 ± 2.5** (14.3 to 19.5)	**19.9 ± 3.4** (16.4 to 23.5)	20.2 ± 3.3 (17.4 to 23.0)	20.1 ± 4.2 (16.6 to 24.0)	2.2 (0.2 to 4.1)*	3.1 (0.8 to 5.3)*	-0.1 (-2.0 to 1.9)	-0.1 (-5.5 to 5.2)	1.6 (-3.4 to 6.5)	1.7 (-3.6 to 7.1)
$\dot{V}O_{2}$ peak (mL.min)	1437.3 ± 411.3 (1093.4 to 1781.1)	1563.4 ± 445.7 (1190.7 to 1936.0)	1565.7 ± 365.6 (1182.0 to 1949.3)	1749.0 ± 421.7 (1306.4 to 2191.6)	1605.0 ± 458.4 (1221.8 to 1988.2)	1586.4 ± 474.3 (1189.9 to 1982.9)	126.1 (-37.4 to 289.6)	183.3 (-5.5 to 372.2)	-18.6 (-182.1 to 144.9)	157.0 (-446.3 to 760.4)	95.4 (-463.2 to 654.0)	-61.7 (-665.0 to 541.7)
Slope VE/VCO_2_	30.1 ± 5.8 (25.3 to 35.0)	31.9 ± 5.8 (27.1 to 36.7)	27.1 ± 5.0 (21.8 to 32.4)	30.4 ± 7.2 (22.9 to 38.0)	**28.7 ± 7.1** (22.8 to 34.6)	**32.9 ± 6.7** (27.3 to 38.4)	1.8 (-1.6 to 5.1)	3.3 (-0.6 to 7.2)	4.2 (0.8 to 7.5)*	2.3 (-6.3 to 10.8)	0.2 (-7.7 to 8.2)	-2.0 (-10.6 to 6.6)
METs max	**5.0 ± 1.2** (4.0 to 6.0)	**5.6 ± 1.4** (4.4 to 6.8)	**4.8 ± 0.7** (4.1 to 5.6)	**5.7 ± 1.0** (4.7 to 6.7)	5.8 ± 1.0 (5.0 to 6.6)	5.8 ± 1.2 (4.8 to 6.7)	0.6 (0.0 to 1.2)*	0.9 (0.2 to 1.5)*	0.0 (-0.6 to 0.5)	0.0 (-1.6 to 1.5)	0.5 (-1.0 to 1.9)	-0.5 (-1.1 to 2.0)

Values are expressed as mean ± standard deviation (SD), mean difference (MD) and 95% CI (confidence interval). Comparisons between groups were analyzed by the mixed ANOVA with Bonferroni post-test (within and between) for all parameters.

*p ≤0.05. HIIT, high-intensity interval training; CRT, circuit-resistance training; CG, control group; CPET, cardiopulmonary exercise testing; RER, respiratory exchange ratio; HR, heart rate; VT1, first ventilatory threshold; VT2, second ventilatory threshold; $\dot{V}O_{2}$, oxygen uptake; mL, millimeter; kg, kilogram; min, minute; METs max, maximal metabolic equivalent.

**Fig 5:**
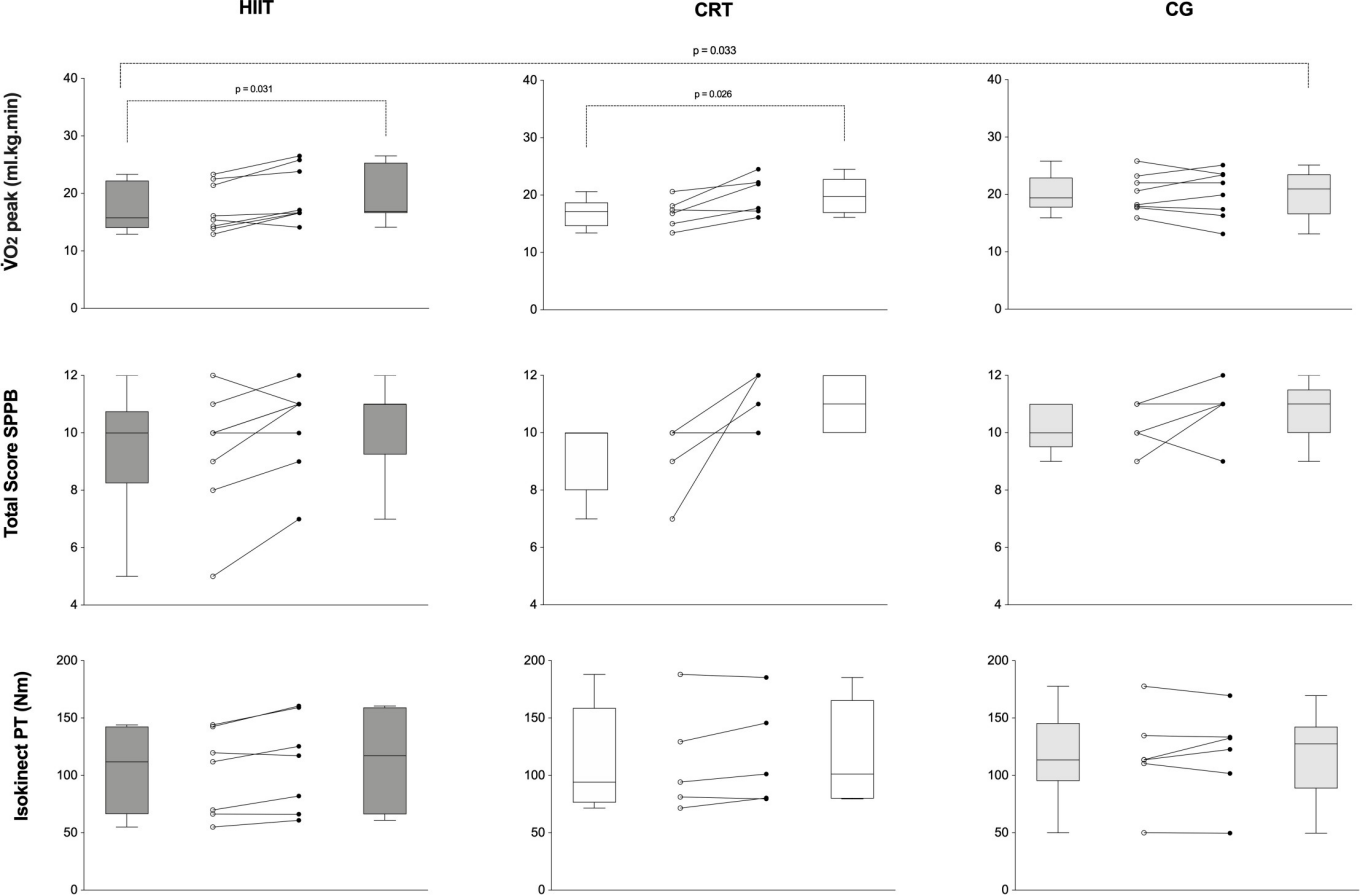
Individuals dynamics per groups. HIIT, high-intensity interval training; CRT, circuit-resistance training; CG, control group; $\dot{V}O2$, oxygen uptake; mL/kg/min, millimeter per kilogram per minute; SPPB, short physic performance battery; PT, peak torque; Nm, newton-meter; p, statistical significance.

**Fig 6:**
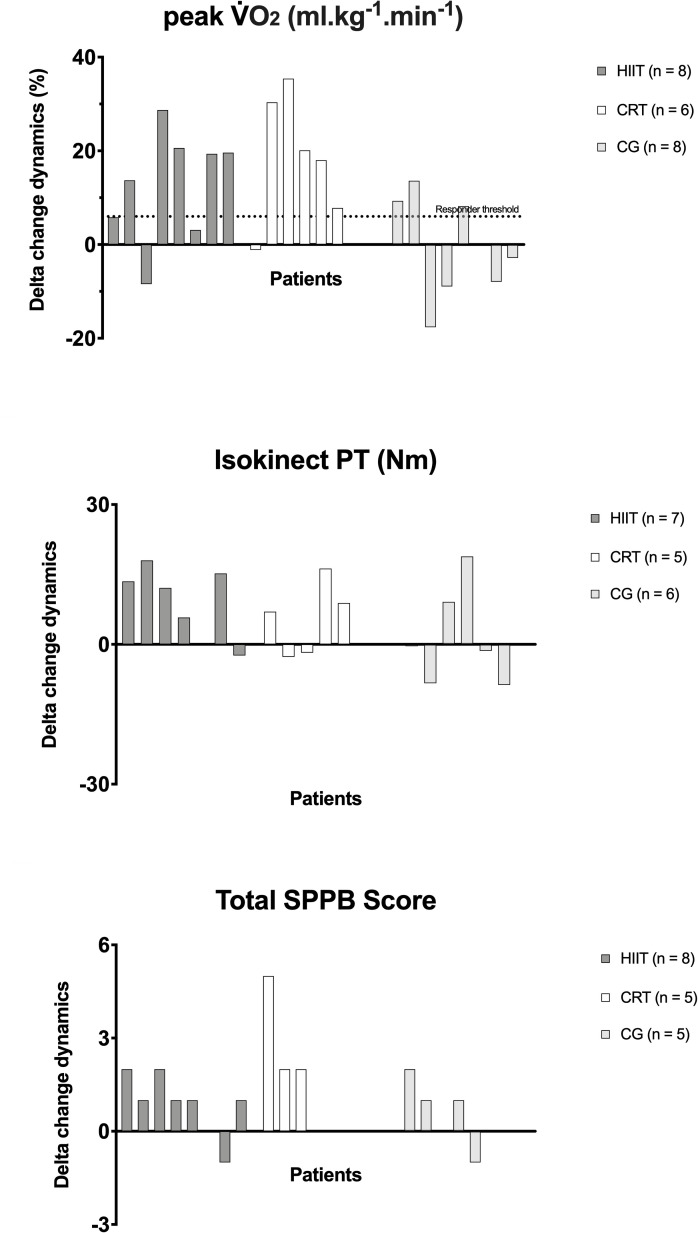
Delta change variation. Responder threshold for $\dot{V}O2$ (minimum 6% increase mL/kg/min). HIIT, high-intensity interval training; CRT, circuit-resistance training; CG, control group; $\dot{V}O2$, oxygen uptake; mL/kg/min, millimeter per kilogram per minute; W, watt; SPPB, short physic performance battery.

The individual dynamic of $\dot{V}O_{2}$ peak along the groups are demonstrated on Fig 5. In order to complement this, the responders treshold is illustrated on Fig 6 for VO2 peak, i.e., the minimum expected improvement range desirable according to previous literature. This responder threshould was based on previous literature [53] which reported that every 6% increase in VO2 peak in heart failure population, was associated with a 8% lower risk of cardiovascular mortality or heart failure hospitalization and a 7% lower all-cause mortality. The majority of patients responded for improvements on $\dot{V}O_{2}$ peak in both intervention groups.

#### Absence of patients on muscle strength and physical performance test

Two patients from CG did not have their muscle strength and physical performance assessed due to absence at the evaluation centre. One patient from HIIT group were not able to execute properly both tests. One patient from CRT group referred joint pain in the knee during the post test (due to a small incident at his home). All other patients were normally tested.

### Muscle strength

Table 4 indicates the effects of both training modalities and control period on muscle strength parameters in heart failure individuals. Figs 5 and 6 indicated the individual variations and delta changes for muscle strength, respectively, in all groups. Considering the absence of a prognostic cutoff point in the literature about knee extension muscle strength in heart failure patients, a responder treshould, i.e., the minimum expected improvement range desirable according to previous literature was not indicated for this parameter, but the delta changes are graphically demonstrated on Fig 6. Groups were statistically similar at baseline moment. HIIT led to significant increments in isokinetic torque peak and average power, while in CRT, there was not statistical increase. To conclude, the HIIT group had a superior effect on muscle strength indicators vs. CRT.

**Table 4:** Impact of interventions on muscle strength in heart failure patients.

BIODEX	HIIT		CRT		CG		Within-group difference (post minus pre)			Between-group difference		
	Mean ± SD (95% CI)		Mean ± SD (95% CI)		Mean ± SD (95% CI)		MD (95% CI)			MD (95% CI)		
	pre (n = 7)	post (n = 7)	pre (n = 5)	post (n = 5)	pre (n = 6)	post (n = 6)	Δ HIIT	Δ CRT	Δ CG	HIIT vs. CRT	HIIT vs. CG	CRT vs. CG
Isokinetic torque peak (Nm)	101.4 ± 37.2 (66.9 to 135.8)	110.2 ± 41.6 (71.8 to 148.7)	112.9 ± 47.4 (54.1 to 171.8)	118.5 ± 46.1 (61.3 to 175.6)	116.7 ± 41.4 (73.3 to 160.1)	118.3 ± 40.1 (76.1 to 160.4)	8.9 (-0.2 to 18.0)	5.5 (-5.2 to 16.3)	1.5 (-8.3 to 11.4)	9.9 (-55.9 to 75.7)	11.7 (-50.8 to 74.2)	1.8 (-66.2 to 69.8)
Isokinetic torque peak / Body weight (%)	**117.3 ± 28.8 (90.7 to 144.0)**	**129.4 ± 32.3 (99.6 to 159.3)**	122.6 ± 40.5 (72.4 to 172.9)	129.3 ± 37.3 (83.0 to 175.6)	146.0 ± 29.1 (115.4 to 176.6)	151.9 ± 36.7 (113.4 to 190.4)	12.1 (1.8 to 22.4)*	6.7 (-5.5 to 18.8)	5.9 (-5.2 to 17.0)	2.6 (-50.2 to 55.4)	25.6 (-24.6 to 75.7)	23.0 (-31.6 to 77.5)
Total Work (J)	1742.5 ± 590.5 (1196.3 to 2288.6)	1902.1 ± 610.2 (1337.8 to 2466.5)	2016.3 ± 733.7 (1105.0 to 2927.5)	1981.3 ± 1181.1 (514.8 to 3447.9)	2060.5 ± 664.4 (1363.3 to 2757.7)	2117.9 ± 731.8 (1350.0 to 2885.8)	159.7 (-224.7 to 544.1)	-34.9 (-489.8 to 419.9)	57.4 (-357.8 to 472.6)	273.8 (-1358.7 to 811.1)	-318.1 (-1348.8 to 712.8)	-44.3 (-1166.2 to 1077.7)
Work / Body weight (%)	132.3 ± 32.5 (102.3 to 162.4)	148.5 ± 39.7 (111.8 to 185.2)	139.6 ± 39.1 (91.0 to 188.1)	144.7 ± 38.5 (97.0 to 192.5)	166.8 ± 31.5 (133.7 to 199.9)	176.9 ± 40.0 (134.9 to 218.8)	16.2 (-3.4 to 35.9)	5.2 (-18.1 to 28.4)	10.1 (-11.2 to 31.3)	1.7 (-54.4 to 57.8)	31.4 (-21.9 to 87.7)	29.7 (-28.4 to 87.7)
Total Work Max Repetition (J)	115.1 ± 45.8 (72.4 to 157.4)	124.6 ± 42.1 (85.7 to 163.5)	127.4 ± 45.2 (71.3 to 183.5)	132.7 ± 49.9 (70.6 to 194.6)	132.9 ± 42.6 (88.3 to 177.6)	138.0 ± 45.2 (90.6 to 185.4)	9.5 (-6.8 to 25.9)	5.5 (-14.1 to 24.6)	5.1 (-12.6 to22.7)	10.2 (-59.5 to 80.0)	15.6 (-50.6 to 81.9)	5.4 (-66.7 to 77.5)
Work Fatigue (%)	35.3 ± 14.3 (26.7 to 43.9)	41.3 ± 4.2 (38.8 to 43.7)	39.2 ± 6.8 (34.3 to 44.1)	35.7 ± 11.6 (23.5 to 44.0)	39.2 ± 12.7 (25.8 to 52.5)	43.4 ± 4.9 (38.3 to 48.5)	8.1 (-4.3 to 20.5)	-3.1 (-17.7 to 11.6)	4.2 (-9.2 to 17.6)	-0.1 (-14.2 to 14.0)	4.1 (-9.4 to 17.5)	4.2 (-10.5 to 18.8)
Average power (Watt)	**141.1 ± 50.8 (94.2 to 188.1)**	**161.9 ± 63.2 (103.4 to 220.4)**	175.2 ± 77.4 (79.1 to 271.4)	191.3 ± 75.7 (97.3 to 285.2)	167.6 ± 59.7 (105.0 to 230.2)	169.0 ± 60.5 (105.5 to 232.6)	20.8 (4.7 to 36.8)*	16.0 (-2.9 to 35.0)	1.4 (-15.9 to 18.7)	31.7 (-68.3 to 131.8)	16.8 (-78.2 to 111.9)	-14.9 (-118.3 to 88.5)

Values are expressed as mean ± standard deviation (SD), mean difference (MD) and 95% CI (confidence interval). Comparisons between groups were analyzed by the mixed ANOVA with Bonferroni post-test (within and between) for all parameters.

*p ≤0.05. HIIT, high-intensity interval training; CRT, circuit-resistance training; CG, control group; Nm, newton-meter; J, Joules; Max, maximum.

### Physical performance

Groups were similar at baseline. Figs 5 and 6 indicated the individual variations and delta changes, respectively, for muscle strength in all groups. Considering the absence of studies indicating an appropriate prognostic cutoff point for physical performance on this population, a responder treshould was not indicated for this parameter, but the delta changes are graphically demonstrated on Fig 6. Differences among groups were seen for the parameters balance test and total SPPB (Table 5). HIIT group demonstrated improvement for the parameter total SPPB score (p = 0.041) which was not statistically indicated for CRT (p = 0.250). CRT reduced the time to complete the chair stand test (p = 0.050), demonstrating an improvement on this parameter. To conclude, CRT was more effective in improving physical performance when compared to HIIT.

**Table 5:** Impact of interventions on short physical performance battery in heart failure patients.

SPPB	HIIT		CRT		CG		Within-group difference (post minus pre)			Between-group difference		
	pre (n = 8)	post (n = 8)	pre (n = 5)	post (n = 5)	pre (n = 5)	post (n = 5)	Δ HIITMD (95% CI)	Δ CRTMD (95% CI)	Δ CG MD (95% CI)	HIIT vs. CRT	HIIT vs. CG	CTR vs. CG
**Balance test**												
Score [Q1-Q3]	4.0 [2.5–4.0]	4.0[4.0–4.0]	4.0 [3.0–4.0]	4.0 [4.0–4.0]	4.0 [4.0–4.0]	4.0 [4.0–4.0]	-	-	-	0.046*		
**Gait test**												
Score [Q1-Q3]	3.5 [3.0–4.0]	3.5 [3.0–4.0]	3.0 [3.0–4.0]	4.0 [3.0–4.0]	3.0 [3.0–4.0]	4.0 [3.0–4.0]	-	-	-	0.739		
Seconds (95% CI)	3.6 ± 0.6 (3.1 to 4.1)	3.7 ± 0.9 (2.9 to 4.4)	3.5 ± 0.6 (2.8 to 4.2)	3.4 ± 0.6 (2.7 to 4.1)	3.6 ± 0.7 (2.7 to 4.5)	3.4 ± 0.7 (2.6 to 4.2)	0.0 (-0.8 to 0.8)	-0.1 (-1.1 to 0.9)	-0.2 (-1.2 to 0.8)	-0.2 (-1.1 to 0.6)	-0.2 (-1.0 to 0.7)	0.0 (-0.9 to 1.0)
**Chair stand test**												
Score [Q1-Q3]	2.5 [2.0–3.0]	3.0 [2.3–3.8]	2.0 [1.0–3.5]	3.0 [3.0–4.0]	3.0 [2.0–3.3]	3.0 [2.5–4.0]	-	-	-	0.083		
Seconds (95% CI)	13.5 ± 2.0 (11.8 to 15.1	12.8 ± 2.4 (10.8 to 14.8)	**14.3 ± 2.9 (10.7 to 18.0)**	**11.0 ± 1.4(9.3 to 12.7)**	12.9 ± 1.6 (11.0 to 14.9)	12.6 ± 2.1 (10.0 to 15.3)	-0.6 (-3.1 to 1.9)	-3.3 (-6.5 to -0.2)*	-0.3 (-3.4 to 2.9)	-0.5 (-3.1 to 2.1)	-0.4 (-3.0 to 2.2)	0.1 (-2.8 to 3.0)
**Total SPPB**												
Score [Q1-Q3]	10.0 [8.3;10.8]	11.0 [9.3;11.0]	10.0 [8.0;10.0]	11.0 [10.0;12.0]	10.0 [9.5;11.0]	11.0 [10.0;11.5]	-	-	**-**	0.008*		

SPPB, short physical performance battery; HIIT, high-intensity interval training; CRT, circuit-resistance training; CG, control group. Values are expressed as median and interquatile range (Q1-Q3) for scores and mean ± standard deviation (SD), mean difference (MD) and 95% CI (confidence interval) for seconds. Comparisons between groups were analyzed by mixed ANOVA for parametric distribution, while Friedman two-way test was applied for the difference among groups (HIIT, CRT and CG) which does not allow the comparison between groups pairwise.

*p ≤0.05.

All tables were also made in a different format containing the p values, and can be seen in S2–S5 Tables in S1 Data.

### Body composition

No changes were observed for dexa scan parameters. The results obtained from DXA scan are illustrated on S1 Table in S1 Data.

## Discussion

The novelty of this study is the comparison of high-intensity protocols of aerobic and resistance-training modality in heart failure patients. This preliminary study indicated that aerobic high-intensity interval training promotes similar effects than circuit-resistance training over cardiorespiratory fitness, although only HIIT indicated better responses on muscular strength improvement. Both training impacted physical performance but better global effects were seen on HIIT. Taking together, HIIT trend to demonstrate superior benefits over high intensity circuit-resistance training in heart failure.

HIIT’s role as an exercise prescription modality for heart failure patients has increased and started to be recommended for low-risk heart failure patients [22]. Although the HIIT has been more explored in HF with reduced ejection fraction [54, 55] and needs to be carefully applied in HF patients with NYHA III [56], the present study, which included all ejection fraction classifications for HF, did not indicated risks related to training. Despite the high intensities, HIIT and CRT were well tolerated, which can be related to the absence of severe muscle dysfunction and the mean age of the patients included (general mean age around 57 years old). High intensity training, independent of aerobic or resistance modality, demonstrated to be safe in HF.

Interestingly, similar effects on exercise capacity was evidenced in both training groups, with improvements in $\dot{V}O_{2}$ peak and METs max. However, only HIIT demonstrated improvements on cycling peak power output and isokinetic peak torque, positively impacting muscular strength and better prognosis [57]. Muscular adaptations promoted by HIIT, as similar effects on microvascular and mitochondrial adaptations in type I and type II muscle fibers [58], and less obesity present on this group (mean BMI lower than 30) may have influenced such greater muscle strength responses [59]. Considering HIIT was based on alternately sessions on the treadmill and cycle ergometer, patients from this group were submitted to half of the training period on dynamic contractions of quadriceps and hamstring, which may also have influenced the positive anwers on muscle strength. Also, considering higher aerobic loads were necessary to maintain an adequate heart rate range on HIIT along the training period, higher muscle demands were required reversing skeletal myopathy [60] and impacting muscle strength gains. Improvements on physical performance were seen on both training groups. Taking together, HIIT indicated better global responses than CRT.

Resistance-training intervention as a single intervention have demonstrated to increase aerobic capacity [28], and our results also confirm this increase when adopting a progressive high intensity circuit-resistance training. Also, the best training effects on chair stand component from the functional performance test seen on CRT, corroborate with previous findings that resistance training shows good response on physical disability in HF patients [61]. Some evidence points to high resistance loads as more useful to increase acutely myofibrillar protein synthesis causing neural adaptations, and further muscle strength [62–66], however our fundings did not indicated muscle strength improvements after CRT. Considering the superior prognostic value of the $\dot{V}O_{2}$ peak and muscle strength [67] in HF by following gold standard measurements (cardiopulmonary exercise testing and isokinetic peak torque), our findings indicate better results for HIIT.

One of the main targets of this study was also to verify the impact of both high intensity traininig on endothelial function of HF patients. Considering that endothelial dysfunction in HF [68] is mainly caused by lower production of nitric oxide, increasing oxidative stress, and vasoconstriction response [69] and has been associated with increased mortality hazard, potential effective approaches to tackle the dysfunction is wanted. The majority of studies evaluating the training effects on endothelial function applied aerobic training modality indicating improvements on this variable [70–72] however, neither HIIT or CRT findings have indicated changes on this parameter. Resistance training combined with aerobic training already demonstrated improvement on FMD [73], but the isolated effect of high-intensity resistance training was not previously demonstrated. Our findings did not indicate the potential to increase endothelial function parameters in HF. Different cardiovascular aetiologies, ejection fractions and the small sample size for this outcome may also have influenced the results.

These preliminary findings’ main clinical implications shows that reaching high loads of training can improve exercise capacity in HF, independently of the training modality. However, HIIT trend to be more attractive to impact the global health status than high intensity circuit resistance training in HF patients by jointly increasing exercise capacity, muscle strength and physical performance. In the era in which refined exercise prescription is under intense debate, this study stresses the clinical practice revealing that although its safety high loads of training needs to be carefully applied in HF after a detailed investigation about the patient needs. Clinical trials with more patients are expected to keep building knowledge on exercise prescription with high-intensity loads in HF, mainly for circuit-resistance training modality.

### Study limitations

Sources of bias was analyzed by Rob2 tool, and we identified bias related to missing outcome data criteria for the outcomes FMD, SPPB and muscle strength due to different reasons as reported on consort flowchart and results session. The main limitation of this study was the covid-19 outbreak, which forced this multicentre in-progress collaboration with Belgium be early terminated and only data from one center could be provided. This made us recognize the imprecision of the findings due to the relatively small sample size which restricts the generalization of our findings to the entire heart failure patient population. Despite those limitations, the current study offers a unique perspective in heart failure population comparing high intensity training protocols also providing head-to-head results on exercise capacity, endothelial function, muscular strength and physical performance. This preliminary study indicated trends that helps building up knoledgement about the therapeutic value of high-intensity protocols on cardiovascular rehabilitation.

## Conclusions

This preliminary study indicates that high-intensity interval training seems to promote a superior effect than high-intensity progressive circuit-resistance training by increasing cardiorespiratory fitness, muscular strength, and physical performance in heart failure patients. To a greater extend, no training effects were detected on endothelial function. Further studies with larger cohorts of patients are however needed.

## Supporting information

S1 Dataset (XLSX)

S1 File Consort checklist. (DOC)

S2 File Study protocol. (DOCX)

S3 File Chronological dates of the study steps. (DOCX)

S1 Fig Individual dynamic of flow mediated dilation. Flow-mediated dilation individual dynamics. HIIT, high-intensity interval training; CRT, circuit-resistance training; CG, control group.
(TIF)

S1 Data Body composition parameters in heart failure. DXA (dual x-ray absorptiometry); HIIT (high-intensity interval training); CRT (circuit-resistance training); CG (control group). Comparisons between groups were analyzed by the Two-way ANOVA (group*time interaction and group interaction). The baseline and post were analyzed by Kruskall-Wallis Test. The normality was analyzed by Kolmogorov-Smirnov test. Values are expressed as mean ± standard deviation (SD). A statistically significant difference was considered when there was a p value <0.05. Obs. Considering some patients made use of pacemakers, only 16 patients made biodex assessment. No differences among baseline parameters were found, neither differences related to the intervention.
(DOCX)

S1 List (DOCX)
