# Supplementary material for: High-intensity interval training versus progressive high-intensity circuit resistance training on endothelial function and cardiorespiratory fitness in heart failure: A preliminary randomized controlled trial
Source: PLoS One. 2021 Oct 1;16(10):e0257607. doi: 10.1371/journal.pone.0257607 (PMC8486136; doi:10.1371/journal.pone.0257607)
Supplement: S2 File — (DOCX) [file pone.0257607.s003.docx]

Doctoral Research Project

COMPARISON OF HIGH-INTENSITY VS. AEROBIC TRAINING TRAINNING VERSUS CIRCUIT RESISTANCE TRAINING ON ENDOTHELIAL FUNCTION AND THE OXYGEN KINETIC IN HEART FAILURE : a randomized controlled trial

NATÁLIA TURRI DA SILVA

Collaborators: Amanda Oliveira do Vale Lira, Lilian Bocchi Portugal, Marianne Lucena da Silva, Tatiana Zacarias Rondinel , Alexandra Corrêa de Lima , Júlia Fontenele dos Santos, Amanda Rafaely Baldoino , Daniela Mendonça , Jessica Desirr and Lima Macedo de Oliveira , Luciana D'Ávila , Sérgio Henrique Rodolfo Ramalho, Robson Fernando Borges, Dominique Hansen, Graziella França Cipriano

University of Brasilia - UnB , Faculty of Ceilândia - FCE . Project presented to the Graduate Program in Science and Technology in Saúde.Área concentration: Promotion, Prevention and Intervention in Saúde.Linha Research: Health, Functionality, Occupation and Cuidado.Temática : Clinical Exercise Physiology.

Advisor: Prof. Dr. Gerson Cipriano Júnior

Brasilia December of 2017

ABSTRACT

INTRODUCTION : Individuals with heart failure (HF) have dyspnea and fatigue, symptoms that decrease exercise tolerance and functional physical performance. Among the modalities of training physical that can contribute to this framework, the aerobic training high intensity interval (TAAI) and resistance training circuit (TRC) are alternatives that have shown benefits . But there are still gaps important about the its effects on endothelial function (EF) and kinetics of oxygen (ΔVO2) and fill this gap can bring relevant information quantotratamento these individuals. The potential improvement in EF and ∆VO2 can result in a better blood and oxygen supply to peripheral muscles

. OBJECTIVE : To analyze and compare the TAAI and CRT modalities in the endothelial function and oxygen kinetics outcomes in patients with HF. METHODS : Randomized controlled, longitudinal, parallel clinical trial involving HF patients with reduced ejection fraction aged over 35 years , divided into 3 groups: high intensity interval aerobic training, resistance circuit training and control group . The trainings will take place 3 times a week, totaling 36 sessions. The s reviews

of endothelial function and oxygen kinetics occur in times pre and post intervention. The endothelial function will be assessed by flow-mediated dilation (DMF) and the assessment of oxygen kinetics will be performed on an exercise bike following an exercise protocol under constant load.

- 1. INTRODUCTION

Cardiovascular diseases have been the leading cause of death in Brazil since the late 1960s, among which heart failure stands out for its higher prevalence (HF) (1) . The IC generates dyspnea and fatigue, symptoms that decrease exercise tolerance, r epercuti ing performance physical functions of these patients (1) . The physical training through exercises is essential for improving this situation

, and are strongly recommended (Class I indication) for patients after a cardiovascular event (2) . The literature indicates benefits obtained both by mode aeróbi to or resistid to, including aerobic training interval of high-intensity resistance training circuit (TRC) form part . However , it is not known which of these modalities generate better systemic responses in HF.

Regarding high-intensity interval aerobic training (TAAI) , a recent meta-analysis ( Xie 2017) indicated better responses in cardiorespiratory capacity when compared to training with lower intensities in patients with HF (3) . In this meta-analysis, higher values of peak VO2 were observed

, which occurred regardless of age or anaerobic threshold (3) . This finding corroborates with the information that intensity seems to be an important predictor of the effectiveness of cardiac rehabilitation programs , even after adjusting for other training-related variables (4) . More recently, multicenter study showed no taai benefits in patients with HF compared ca moderate intensity. And ntretanto, this study 51% taai trained below the intensity adequate while 80% of moderate aerobic training trained intensity above the desired (5) . Thus, TAAI in this population still seems to be a therapeutic highlight (3,4,6) .

As for mode resistance training , although when isolated be beneficial to gain muscle strength, it is known that this method exerts meno rinfluência of which the training aerobic to gain cardiorespiratory fitness (7) , thereby reinforcing the advantage of combining dest the d your training modalities . In this sense the TRC stands , po ise able to stimulate r also adaptations of aerobic and cardiovascular systems as demand higher values of heart rate during training (8) . This is because the maximum HR values normally occur during the last repetitions of a series (8) . The TRC also has shown benefits in heart failure in muscular strength skeletal, VO2 peak (9-11) in

addition to having been expressed strong correlation between the change in mitochondrial ATP production rate of skeletal muscle and the change in maximum oxygen consumption total body (peak VO2) (11) . Such findings demonstrate that CRT seems to be an interesting modality for patients with HF .

Despite the benefits already reported from both TAAI and TRC, there are still important gaps in the effects of both training on the CI population. Understand all the effects of the methods of physical training in the health of these patients is important because it contributes to the proper therapeutic choice in the rehabilitation of the same . In this regard we have not found studies evaluating the responses in endothelial function and either as the oxygen kinetics between these modes in these individuals . The importance of studying such outcomes will be elucidated in the following paragraphs.

The study of endothelial function in patients with HF is essential, since stasis is reduced. The reduction in endothelial function is the result of a lower production of nitric oxide (12) and an increase in oxidative stress (13–15) , which generates an increase in the response to vasoconstriction and vascular resistance . This compromises peripheral vasodilation assessed by means of flow- mediated dilation (DMF) , which leads to a reduction in blood supply to the muscle. Therapeutic resources, such as exercise, are able to improve endothelial function, which may result in a better blood supply to the peripheral and cardiac muscles (15) (16) . The improvement in endothelial function reduces cardiac dysfunction in HF (12), which reinforces the relevance of its assessment in TAAI and CRT interventions.

In addition to the importance shown in studying endothelial function in patients with HF , studying the kinetics of oxygen consumption ( Δ VO2), that is, the magnitude and nature of the adjustment in oxygen consumption during exercise (17,18) , is also important. also relevant for these patients . This is because the perfusion and diffusion of oxygen (O2) is impaired in this population thanks to disturbances within the O2 transport path, which reduces the physical capacity of these individuals

(19) . The improvement in oxygen consumption kinetics minimizes the damage caused by HF in these individuals, emphasizing the relevance of its assessment after physical training interventions

(19) . This review can provide and and lucidar the adaptations within the O2 utilization and distribution system for skeletal muscle providing relevant information in the study of this population (19), in addition to providing r additional resources for interpretation of cardiopulmonary exercise test.

The resolution of the mechanisms underlying skeletal muscle dysfunction and exercise intolerance is essential for the development and improvement of the most effective treatments for patients with HF.

Taking into account the above, and seeking to remedy the gaps in the literature regarding TAAI and CRT, the present study will aim to analyze and compare such modalities in the endothelial function and oxygen kinetics outcomes in patients with heart failure.

The main hypothesis is that individuals allocated to TAAI group will promote better results compared to the TRC, generating greater increases in endothelial function and optimization of oxygen kinetic curve.

- 1. GENERAL OBJECTIVE

Analyze and compare high-intensity aerobic training and resistance circuit training in heart failure patients

- - 1. SPECIFIC OBJECTIVES Primary Objective:

Analyze and compare endothelial function , oxygen kinetics and functional capacity before and after physical training interventions (TAAI and CRT) and control group.

Secondary Objective:

Analyze and compare responses in autonomic modulation, body composition, muscle quality, quality of life, handgrip strength, muscle strength and functional physical capacity in patients with HF before and after physical training interventions (TAAI and CRT) and control group.

- 1. MATERIAL AND METHODS
     1. DESIGN STUDY

This is a randomized controlled, multicentric, longitudinal, parallel clinical trial with a quantitative approach . Study will follow the recommendations issued by the CONSORT 16 for testing Clini c the high methodological quality, with the participation of IC holders of individuals allocated in three groups: training protocols of high intensity interval aerobic (TAAI) , protocol training resisted circuit (TRC) and control group without intervention (GC).

- - 1. SAMPLE

The sample will be composed of individuals diagnosed with HF with reduced and preserved ejection fraction , hemodynamically stable , referred by cardiologists from Brasília and region (Federal District, Brazil) , as well as from the city of Hasselt (Belgium). According to data from a pilot study conducted in Brazil , a total sample of 42 individuals is required, considering an effect size of 0.36 alpha 0.05 power 0.8 considering the FMD outcome variable. To comply with the proposed sample size, the total sample will be composed of 18 participants in Brazil and 24 in Belgium.

- - 1. INCLUSION AND EXCLUSION CRITERIA

Inclusion criteria will be adopted: individuals diagnosed with HF with reduced and preserved ejection fraction , of both sexes (women in menopause), aged over 35 years , who should not have performed any type of physical activity the last 6 months, non-smoking individuals, absence of respiratory diseases and inflammatory or infectious lesion and process muscle tendon t nea or osteoarticular that desempenhasse exercise limitation . The regular accepted medications will only be used to control the necessary cardiovascular factors according to the cardiologist's indication . Exclusion criteria are: patients who perform any kind of physical activity in the rehabilitation period out those you s will be assigned, individuals who do not meet the periodization missing more than 25% of the sessions.

- - 1. RESEARCH LOCATION

The research will be conducted at the University of Brasilia, Campus Ceilândia and also at the University of Hasselt , Campus Diepenbeek , and physical training will take place at the Gymnasium Therapeutic University and Reval ( Bégica , Uhasselt ) . The evaluations for the outcomes mentioned above will be carried out at the Laboratory of Physiology and Biophysics of the University of Brasília, Ceilândia Campus and also at Jess and Hospital, Hasselt , Belgium .

- - 1. ETHICAL CONSIDERATIONS

All procedures used in this study will be forwarded to approve tion by the Ethics Committee of the Institution. Study participants will sign a free and informed consent form confirming their participation and proving their knowledge at all stages of the study, and if they choose, they may withdraw during the work.

- - 1. EVALUATION PROCEDURES AND PROTOCOL

An independent researcher will prepare the allocation of randomized random sequence , which will be obtained through the software random.com . The allocation of the type of treatment will be carried out on the first visit to the physiotherapist, at least two weeks before the beginning of the interventions. The responsible physiotherapist will know the intervention adopted by the volunteers, however the evaluator of each outcome will be blind.

The evaluations will be made in two moments for both groups: before starting the experimental protocol and after the end of it . The outcomes to be analyzed at these times will be: endothelial function, kinetics of oxygen consumption, functional capacity , quality of life, autonomic modulation , body composition , functional physical capacity , muscle quality , hand grip strength.

- - - 1. Flow-mediated vasodilation (DMF)

Previously, the subjects will be instructed to avoid any planned exercise sessions and will be asked to refrain from consuming caffeine / alcohol and to exercise for 24 hours before the tests. The subjects will be advised to participate in the assessment as long as at least 2 hours post-prandial. To avoid potential daytime variations, the tests will always be performed at the same time of day and in the same room with controlled temperature (~ 24 ° C ).

Initially, individuals will rest in a supine position for a period of 15 min to ensure the achievement and stabilization of cardiovascular variables. The brachial artery diameter and blood velocity will be measured using high- resolution duplex-Doppler ultrasound ( Ultrasound System HD11.XZ, 1 and 3 MHZ, Phillips, Barueri, SP, Brazil) following the present guidelines (20) . A 9 MHz linear matrix transducer will be placed over the brachial artery slightly proximal to the cubital fossa. The diameter and speed signals will be obtained simultaneously in duplex mode at a pulsed frequency of 5 MHz and corrected with an insonation angle of 60 °. The sample volume will be adjusted to cover the entire lumen of the vessel without extending beyond the walls and the slider will be adjusted in the middle of the vessel. The FMD of the brachial artery will be evaluated in the right arm in the supine position as previously described (21,22) .

Briefly, a cuff will be attached to the arm. For two minutes rest hemodinâmic data to be registrad them , and then the cuff is inflated to a pressure of 220 mmHg and maintained thus for 5 min. The measurements of continuous diameter and blood velocity will be recorded continuously for 3 min after rapid deflation of the cuff. The analysis of all vascular variables will be analyzed offline using the specialized edge detection software (Cardiovascular Suite , Quipu , Pisa, Italy). The variation in the percentage of DMF was normalized for the incremental area of shear rate under the curve up to the peak diameter (23) (24)

- - - 1. Incremental Cardiopulmonary Exercise Test

The assessment of functional capacity will be carried out by means of an ergospirometric examination carried out by a specialist doctor (blinded as to the allocation). This test will be important for determining the aerobic and anaerobic thresholds required for the prescription of TA AI , in addition to providing outcome measures for the groups, among which VO2 peak and VE / CO2 will be used, reported in a recent systematic review in training protocols. as the most widely used parameters and indicative of results (25) .

Patients will report to the Physiology and Biophysics Laboratory of Unb , to perform the incremental test limited by symptoms, on an electromagnetic bicycle with a 0-watt system ( Corival

, Lode Co., Groningen, Netherlands) using a ramp protocol (5- 10 watts / minute). Before the start of each test, a period of 5 minutes will be observed for adaptation to the cycle ergometer and the stabilization of gas exchange. The 12-lead electrocardiogram will be monitored continuously (T12, Cosmed , Rome, Italy) with a record associated with the gas capture program 27. Blood pressure will be checked with a standard sphygmomanometer, with the patient sitting on the ergometer, every 2 minutes during the exam and up to 15 minutes after the end of the active part of the test. Exhaled gases will be collected in aliquots at each breath by a computerized gas analyzer (Quark CPET, Cosmed , Rome, Italy ).

- - - 1. Evaluation of the kinetics of oxygen

The evaluation of oxygen kinetics will be performed on a stationary stationary bicycle. For this, the volunteers will be instructed to remain seated, with a mask to capture the expired gases by a computerized gas analyzer (Quark CPET, Cosmed , Rome, Italy) . Volunteers will be instructed to wear appropriate clothing for this exercise test prior to the assessment date . Not be will allowed the movement of people around the room during the performance test , in order to reduce the anxiety of individuals and capture errors.

The evaluation of oxygen kinetics will be performed according to the exercise protocol under constant load on the stationary stationary bike , performed with an initial phase without load (0 watt start-up system, Lode , Netherlands) for three minutes and immediately after an initial exercise. with constant load, performed below the LA (anaerobic threshold), for 6 minutes with a constant sub-LA load (1st session). Shortly after the moderate exercise session, an interval of 15 minutes will be performed and a new exercise in the sequence, carried out with an initial load phase 0 watt for three minutes and after that an exercise with supra-LA load (80% Δ VO2max) until the maximum possible (Tlim) and after the exercise the collection of the expired gases will be carried through for 15 minutes.

During the test, oxygen consumption (O 2), carbon dioxide production (CO2), minute ventilation (E), tidal volume (VC), respiratory rate (  ), respiratory exchange ratio (R), will be analyzed , equivalent ventilation for oxygen (E / O2) and carbon dioxide (E / CO2), inspiratory time (TI), expiratory time (TE), and TI / TTOT ratio. The data will continue to be collected even after the end of the exercise, at least 15 minutes of passive recovery.

Throughout the protocol, the perceived effort index for fatigue of the lower limbs will be recorded. The 12-lead electrocardiogram will be continuously monitored. Individuals will be asked about the sensation of ventilatory effort and tiredness in the lower limbs every 2 minutes, according to the Borg scale . The system will be calibrated daily before each test.

- - - 1. Evaluation of Muscular Strength

Muscular strength will be assessed using the 1RM test, which will determine the maximum load that each individual will be able to perform during the movement required by the exercise in question, to later determine the training loads. This test represents the greatest resistance that can be moved through the full range of motion in a controlled manner and with good posture.

The test starts by receiving increments according to the subject's perception, until it is concluded when the volunteer reaches the maximum load, in which he can execute the movement without mechanical failure. No more than five attempts will be allowed to establish this maximum load and if this occurs, the test will be disregarded with scheduling a new date for evaluation. This variable will be collected at the baseline moment before the start of training and 72 hours after the end of the last session.

- - - 1. Evaluation of autonomic modulation

The evaluation of autonomic modulation will be performed through Heart Rate Variability (HRV). Therefore, volunteers will be directed to remain at rest, awake supine and spontaneous breathing for 30 minutes by cardiofrequency the Polar® RS800 (Polar Electro Oy, Finland) on the wrist , previously validated equipment as its use to capture the intervals between consecutive heartbeats (in ms ) (26) . The volunteers will be guided by not consumption SNA 24 stimulants prior to this evaluation. People will not be allowed to move around the room during collections, in order to reduce individuals' anxiety and collection errors. The data obtained from HRV will be transferred to a computer using the Polar Pro Trainer software and, later, to calculate HRV indices, the Kubios HRV software - version 2.0 ( Kubios , Biosignal Analysis and Medical Image Group , Department of Physics , University of Kuopio , Finland ) , considering 1000 sinus intervals (minimum 95% sinus beats). This will occur after digital filtering (Polar Pro Trainer software ) complemented by a manual (Microsoft Excel), to eliminate premature ectopic beats and artifacts.

For HRV analysis, linear indices will be used, obtained in the time domains (RMSSD and SDNN)

(27) , geometric indices ( Poincaré plot , triangular interpolation of the NN intervals (TINN) (27) and triangular index ( RRtri ) (28 ) and in the frequency domain evaluated using the fast Fourier transform segmented at low frequency (LF - Frequency between 0.04 to 0.15Hz), high frequency (HF - Frequency between 0.15 to 0.4Hz) and LF ratio / HF expressed in normalized units and ms

(27) Non-linear methods already validated will also be used such as Recurrence (REC), Purified frequency analysis ( DFA alpha 1 and alpha 2), Determinism (DET) and entropies ( Apen and Sampen ).

- - - 1. Assessment of body composition

Body composition will be estimated using DXA (DPX-MD, software 4.7; Lunar brand, Madizon , WI, USA). The subject will be positioned in a supine position during the exam, which should be Property ions for a time of about 1 0 minutes. Fat mass (MG) and fat free mass (MLG) will be expressed in absolute values. To classify the groups in relation to body composition, due to the absence of cutoff points, the median of the greater or lesser amount of MG and MLG will be used

.

- - - 1. Evaluation of thickness and eco nicity muscle

The individuals will be evaluated in a supine position with the knee in passive extension and neutral rotation. A the aqueous solution , called transmission gel is applied to the head ultrasound to permitircontato sound without pressing the dermal surface. Two images are adquirid the s right leg : (1) image earlier: transducer placed perpendicular to the long axis of the thigh anterior two -thirds of the distance from the anterior superior iliac spine to superiorborda the patella

(29) , and (2) the side image: 5 cm laterally from the first image point. The edge image is obtained in a field prolongadode view mode at a distance of 10 cm in a running the direçãocrânio -caudal . To enable the replication of the image location in the post-training ultrasound , a mark will be drawn on the volunteer's leg and a photo will be registered for conference at the final moment . The images will be saved on the ultrasound hard disk and transferred for later analysis on a computer using the ImageJ software (NIH, Bethesda, MD) (30) .

All ultrasound measurements will be performed three times, with the average of the scores used in the final analyzes. In the previous image, muscle thickness and echogenicity of the vast intermediate

, rectus femoris , thickness of the subcutaneous tissue and transverse area of the rectus femoris will be evaluated. Tod the parameters of espessuraserão measured cent e meters, and aarea transverse straight femoralserá medid the at cent t m at the widest point and muscle .

The echogenicity will be reported in pixels. The echogenicity will be determined using quantitative analyzes using computer quantified scales. A 2 × 2 cm square pattern for analysis of the rectus femoris and vastus intermedius muscles separately will be used to determine the region of interest

(ROI). The square method has a stronger confidence compared to the tracking method (where the evaluator highlights all the visible muscle area excluding epimysium and artifacts) to define the ROI (31) . If the area to be analyzed fo r smaller than 2 × 2 cm, the largest possible square within the anatomical limits of the muscle will be examined. Mean and standard deviation of the echogenicity of this ROI will be calculated using the histogram function of the ImageJ software (NIH, Bethesda, MD) and expressed as a value between 0 (= black) and 255 (= white) (32) . In the lateral image, the measurements will include vastus lateral thickness and penile angle of the vastus lateralis (angle in degrees between fascicles of muscle fibers and deep muscular aponeurosis) .

The ultrasound measurements will be carried out in two moments: before starting the training protocol and 72 hours after the end of the last session of the same.

- - - 1. Classification of states of functionality and disability (ICF)

The classification of the states of functionality and disability will be assessed through the CIF in its comprehensive version, which consists of the application of all its codes. The assessment of the ICF will be in the form of an interview by a physiotherapist previously trained to use the instrument, according to Martins et al. 2010 (33) .

The ICF codes will be established based on the following components: I - Body functions; II Body structures; III - Activity and Participation; IV - Environmental Factors. Components I, II, and III are related to part 1 of the ICF, intended to classify functionality and disability and component IV are related to the classification of contextual factors.

The researcher will manually record the number of occurrences for all levels of coding, for all subjects. However, when processing the data, only the first level occurrences (chapters) will be considered for classification. Each specified code will be related to its respective qualifier, with qualifier being the determinant of functionality, that is, no deficiency for that code. Qualifiers 1 to 4 will be considered as determinants of disability, as they classify the presence of disability from mild to complete. Qualifier 8 will be considered as unspecified, when the presence of a disability could not be determined, and qualifier 9, as not applicable, when the code was not applicable.

- - - 1. Force to hold palmar

To measure the handgrip strength, the handgrip instrument will be used .

The positioning of the participants will be in accordance with the American Society of HandTherapists Guide l ines (34) : subject seated with supporting arm , adducted shoulders in neutral rotation, elbow flexed to 90 °, forearm in a neutral position and PU Nho between 0 and 30

Dorsiflexion °. Three measurements with the dominant and non-dominant hand will be performed. The highest value will be expressed in kg, and included in the analysis. The maneuvers must be carried out with 5 seconds of support, and 3 minutes of rest between them. We will instruct patients to maintain spontaneous breathing and to avoid performing the Valsalva maneuver , in conjunction with exercise.

Participants will be instructed to maintain positioning during tests and corrected by the examiner when necessary. Accessories such as watches, bracelets, rings and bracelets will be removed from both participants' upper limbs before testing begins. All participants will be assessed individually. Participants will be instructed not to look at the dynamometer display to avoid any visual feedback

.

- - - 1. Short battery of physical performance (SPPB)

The SPPB will be read, and applied, to the subjects in order to reduce the risk of bias. The domains explored by the questionnaires will be scored as foreseen by the SPPB.

Considering that the Short Physical Performance Battery (SPPB) is an instrument composed of three different tests , the evaluations will work as follows : 1) static balance - evaluated in three positions - side by side (feet together), semi tandem (one foot partially in front of the other) and tandem (one foot in front of the other); 2) walking speed (the time taken to cover three meters in a normal pace is timed); 3) strength of the lower limbs (the time spent to get up and sit on a chair for five consecutive times, without the aid of the hands). The score ranges from 0 to 4, for each of the tests and according to the time performed in each task, with 0 being the worst performance and 4 being the best performance. If it is impossible to carry out any of the steps, the score will be zero. (35)

The total score of the test is the result of the sum of the three tests already mentioned, that is, 12 points, with 0 being the worst performance and 12 the best performance. Score of 0 to 3 s and r will be considered very poor performance or failure, score points are 4 to 6 ether shall considered poor performance; 7 to 9 points s and r to the moderate performance and score points 10 to 12 s and r will considered good performance. (35)

- - - 1. Assessment of isokinetic muscle strength and peripheral oxygen extraction

For the assessment of isokinetic muscle strength, the dynamometer BIODEX System 3 PRO, New York will be used .

The subjects will be positioned with their hips at 90 ° with the articular axis aligned with the fulcrum of the dynamometer so that the knee of the evaluated member is free (distance of 2 fingers) and the subject is with the entire gluteus close to the back of the chair. The alignment of the evaluated leg with the contralateral limb will be checked. All measurements of the subject's position on the dynamometer (chair height; chair base; chair back; distance from the dynamometer; distance from the arm) will be recorded on a form made by the researchers in order to make the measurement reproducible after the intervention. The subjects performed knee extension at 70 ° s-1 , to assess the peak isometric torque, 5 times for 4 seconds with a rest time of 30 seconds between repetitions. The average of the 3 highest values will be considered for analysis. For the measurement of dynamic strength, the subjects will perform knee extension at 180 ° s-1 for up to 30 repetitions.

The evaluation of peripheral oxygen extraction will occur in a continuous and non-invasive way through the NIRS ( Near-Infrared Spectroscopy Portamon ( Artinis Medical Systems, Einsteinweg 17, 6662 PW, Elst, The Netherlands ). In the region close to the infrared, hemoglobin - including its two main variants: oxyhemoglobin (O 2 Hb ) and deoxyhemoglobin ( HHb ) - exhibit oxygen dependent absorption. Using a number of different wavelengths, relative changes in hemoglobin concentration can be displayed continuously. in the vastus lateralis muscle of the evaluated leg, visualized the site of the largest muscle belly after isometric contraction, where the fixation of the device will be by means of velcro strips and covered with a black bandage to eliminate the ambient light. This measurement will start 5 minutes before the procedures evaluation of isokinetic muscle strength, will be maintained throughout the evaluation, and 5 minutes after the end. the posicionament that of NIRS will be marked with a demographic pen and registered using a photographic camera. The variables analyzed will be tissue oxygenation index (TSI%), oxyhemoblogin (O 2 Hb), deoxyhemoglobin ( HHb ), total hemoglobin ( tHb ) and hemoglobin differentiation ( HbDiff ). The data will be analyzed with OxySoft Software (v2.1.2, Artinis Sistemas Médicos). The tissue saturation index (TSI) will be calculated using the equation below:

- - - 1. Lung function and respiratory muscle strength

Lung function and respiratory muscle strength are important assessments for the method of selection / respiratory assessment of patients (anamnesis) . Such assessments will be accessed by spirometry tests ( MicroLab ML3500MK8, CareFusion , USA) , manovacuometer (MVD300, Globalmed , Brazil) and dynamic inspiratory pressure .

Individuals will be instructed to abstain from autonomic stimulants (alcoholic beverages, coffee, tea or caffeine-containing foods) for 24 hours before the assessment, as well as eating a light meal at least 2 hours before the measurement. Patients must rest 10 minutes before each test (spirometry, maximum respiratory pressures and dynamic inspiratory pressure). They will be placed on a bench with legs bent at 90 degrees, upright posture, head in neutral position and nasal clip to prevent leaks.

Spirometry

Individuals will perform at least three forced expiration maneuvers according to the American Thoracic Society (ATS) / European Respiratory Society (ERS) technical procedures and acceptability and reproducibility criteria (36) . The spirometric evaluation will be performed to check for obstructive or restrictive patterns through outcome measures: forced expiratory volume in the first second (FEV1), forced vital capacity (FVC), peak expiratory flow (PEF), FEV1 / FVC ratio.

Blood pressure monitor

The MVD300® manovacuometer ( Globalmed , Porto Alegre, RS, Brazil) was used to measure positive pressures (manometer) and negative pressures (vacuometer). The manovacuometer allows the static assessment of maximum inspiratory pressure ( MIP ) and maximum expiratory pressure ( MEP ) and plays an important role in the diagnosis and prognosis of chronic diseases (37) .

The manovacuometer will be previously calibrated in cmH2O, with an operational limit of -300 to

+300 cmH2O and scales ranging from 10 to 10 cmH2O. The methods and criteria used will be those recommended by ATS / ERS (38) . To assess MIP , patients will be instructed to perform a maximum expiration, following a maximum inspiratory effort , so that the pressure is recorded close to the residual volume. For the assessment of MEP , a maximum inspiration will be requested before the maximum expiratory effort to assess the pressure, close to the total lung capacity. A vigorous verbal command will be given during the assessment.

Dynamic inspiratory pressure

The assessment of dynamic respiratory muscle strength will be performed using the POWERbreathe ® KH2 device (London, England, United Kingdom), in which the maximum inspiratory effort will be assessed from the residual volume. Maximum muscle strength (S-index) will be obtained during the dynamic contraction of the inspiratory muscles. Patients will be instructed to achieve the highest possible inspiratory flow rate after a previous expiration. To obtain the S-index , the patient must be collaborative and able to respond to verbal commands (38) .

- - - 1. NT-pro-BNP

NT-pro-BNP will be used to characterize heart failure with preserved or reduced ejection fraction

(39) , specifying patient entry criteria along with other variables (anamnesis) according to the European Society Cardiology Guidelines (40) .

Patients do not need to fast or have any special preparation for the test.

NT- proBNP serum will be measured using test strips (CARDIAC proBNP +, Roche Diagnostics , Basel, Switzerland) containing monoclonal and polyclonal antibodies against epitopes of the NT- proBNP molecule in a point- of - care device ( Cobas h232, Roche Diagnostics Basel, Switzerland). A sample of venous blood will be kept in heparinized (sodium) tubes ( Vacuette from Greiner Bio- One , Roche Diagnostics , Basel, Switzerland) at room temperature and analyzed within 5 hours. The manufacturer's controls will be used to monitor quality control with acceptability limits defined by the manufacturer (4). Assessments will always be carried out in the physiology laboratory by qualified professionals (cardiologists collaborating with the research).

- - - 1. Echocardiographic evaluation

Individuals will be instructed to abstain from caffeine for 24 hours prior to the test.

The strain echocardiographic examination with a 4-2 MHz transducer equipped with a second harmonic image (HDI 5000 2-4 MHZ, Philips ATL, Bothell , WA) will be used to access cardiac function through the DICOM (Digital Imaging and Communication format) in Medicine) ( 31) ( anamnesis ) . Outcome measures include left ventricular ejection fraction by Simpson (LEF,%), left atrial volume index (LAV, ml / m²), left ventricular mass index (IMVE, G / M²), diastolic diameter of the left ventricle left ventricle (LVDD, mm) and blood pressure (PAP, mmH ) following recommendations by the American Society of Echocardiography and the European Association for Cardiovascular Imaging (41,42)

- - 1. INTERVENTION PROTOCOL

The experimental protocol for this study will be submitted to the national clinical trial registration platform <http://www.ensaiosclinicos.gov.br/>.

It is important to note that a pilot study will be carried out as a miniature version of the main study to test whether the components of this study (all evaluations and steps) can work concurrently. This procedure will have all the outcomes of the main study that can contribute to the final analysis as well as sample calculation and, if necessary, adaptations will be made in the study design so that it is as appropriate as possible.

- - - 1. Cardiac rehabilitation

The study will consist of three randomized intervention groups: TAAI - high intensity aerobic training , CRT - resistance training in circuit (T RC ) ; CG - control group.

The training groups will perform exercises three times a week, with an interval of at least 24 hours between sessions until totaling 36 training sessions.

Previously, the training will be familiarized with the equipment as follows: for the TR C the individual will perform 10 repetitions with a load referred to as slightly intense (13 on the Borg scale ) oriented in good posture; for TA AI the individual will be instructed to walk on the treadmill for 15 minutes with somewhat intense perception (13 on the Borg scale ) . These criteria will collaborate for volunteers to perform the movements with the best possible ease, adjusted s by physiotherapists during the familiarization period.

Exercises on the ground and global stretches will be used in order to warm up (15 minutes ) , in order to avoid possible complications , reducing the risk of injury . The interval between the series of exercises will vary from 40 seconds to 1 and a half minutes, respecting the directly proportional relationship between time and exercise load. Familiarization with the equipment will also take place prior to the start of the sessions.

The muscle groups chosen for TR C will be: quadriceps, hamstrings, back, chest, shoulder, biceps and triceps. The test of a maximum repetition (1RM) will be used to measure the maximum load of each individual, to later determine the RT loads. The loads of the TR C protocol will start at 30% of 1RM and gradually increase throughout the sessions up to 80% 1RM, (intensity between moderate and intense), always respecting the principles of adaptation and overload. The loads chosen to perform TA AI , which will occur on a treadmill and exercise bike will vary between two intensities, the highest above the first anaerobic threshold and the lowest below the first anaerobic threshold obtained by the ergospirometric test, with the loads will be changed throughout the sessions according to the HR referring to reach the respective thresholds .

Already regarding the individuals the GC , they will attend lectures and will be instructed to perform light physical activity in an unsupervised manner, 3vezes week, both aerobic as resisted, and the intensity of both considered appropriate to below the first threshold obtained in the examination VO 2. For this, the individual will be previously instructed in the ergospirometric evaluation session regarding the perception of effort and the appropriate limit for exercises.

- - 1. RISKS

Physical training will be held at the Gymnasium Therapy at the University of Brasilia, Campus Ceilandia by trained physiotherapists and during its execution will be accompanied by a medical

collaborator cardiologist (Dr. Alexandra CGB Lima) that will guarantee support physician throughout the work the in order to control the risks of patients.

During the sessions, blood pressure (BP), oxygen saturation (SATO2), effort perception scale ( Borg ) pre and post exercise scale and, if necessary, during the performance will be performed. In addition, the individuals' heart rate (HR) will be assessed pre and post session in addition to being continuously monitored by a cardiofrequency meter throughout the entire session . C Abera the therapist to check the individual limits for each FC ind i vidual (which is previously obtained in accordance with the FC thresholds extracted from cardiopulmonary testing each participant).

The clinical signs and symptoms will be monitored throughout all sessions (Example: excessive tiredness, intense sweating, paleness, dizziness, vision blurred, palpitations , angina or pain pre cordial) and if the volunteer shows no indicative change risks for the year the training will be suspended and the volunteer will be sent to the responsible doctor for medical consultation .

Taking into account the complexity of the patient with heart failure and in order to attend any and all emergencies or complications, the Therapeutic Gymnasium at the University of Brasilia, where the physical training interventions will take place, will be equipped with all the necessary resources

. Resources will be available to therapists to contain events such as hypoglycemia / hyperglycemia and hypertension / hypotension crises (stethoscopes, sphygmomanometers, glucometer for checking blood glucose, emergency blood pressure and glycemic control medications ) , and cardiorespiratory arrest (oxygen cylinder, masks for oxygenation, defibrillator) .

It is worth mentioning that in order to contain a possible cardiorespiratory arrest, all research therapists who will be during the patients' physical training, will have first aid knowledge. This knowledge will give professionals total autonomy to carefully perform cardiopulmonary resuscitation procedures as well as resuscitation, following the recommendations of basic life support for adults. All therapists will have sufficient knowledge to reduce the risk of complications from this event if it occurs.

The individuals allocated to the CG will be monitored over the telephone for symptoms and possible discomfort experienced with training once a month. Also, in face-to-face meetings that will take place on a monthly basis, individuals will be able to clear their doubts with health professionals, which will reduce risks throughout their participation in the research. If necessary, they will be consulted by the team's cardiologist at any time during the research.

We believe that these measures will be sufficient for monitoring patients during training and ensuring safety for these patients.

- - 1. BENEFITS

Research participants will have direct benefits, since they will receive medical care with clinical and physical examinations, in addition to quality physiotherapeutic care throughout the entire cardiovascular rehabilitation protocol. The benefits of the rehabilitation protocol will happen regardless of the intervention group that the participant is selected (high intensity interval aerobic training and resistance training in circuit), since as the introduction of the present project, both modalities are known in the literature for their positive points. in rehabilitation in the population with HF. The rehabilitation protocol will help to reduce the clinical and functional symptoms caused by HF.

The GC not conduct intervention also have will benefit since they will receive medical care with conducting clinical and physical examinations. In addition, once a month they will receive lectures on how to deal with HF. The lectures will address topics such as nutrition, exercise, drug control, psychological aspects and will be held by professionals in the respective fields (nutritionist, physiotherapist, doctor and psychologist). Still, it is worth mentioning that after the research period in which the individuals in the CG need to be sedentary, they will be invited to participate in the exercise protocols (without research purpose), and will receive care supervised by the professionals involved in the study.

All patients, regardless of the group they are allocated, will receive a copy of all tests performed as well as feedback on their health condition and guidance.

We understand that these benefits will be of paramount importance for these patients resulting in the quality of life of the same .

- 1. STATISTICAL ANALYSIS AND SAMPLE CALCULATION

The statistical analysis of the data will be descriptive to characterize the sample . The test of normality of the data will be by Shapiro Wilk. Parametric and / or non-parametric tests will be applied and broken down into tables according to normality. Comparative parametric and / or non- parametric tests will be applied and broken down between groups in tables according to normal (ANOVA two way or Kruskal Walllis ). Possible correlations will be tested using Spearman's correlation . All analyzes will be performed using SPSS statistical software version 22.0 (SPSS, Inc. Chicago, IL, USA), and the significance level adopted is 5%.

- 1. OUTCOMES
     1. PRIMARY OUTCOME

Verification of flow-mediated dilation variables: vessel diameter and blood flow velocity before after taai and trc and control group.

Analysis of oxygen kinetics variables before after taai and trc and control group: oxygen consumption (o2), carbon dioxide production (co2), minute ventilation (e), tidal volume ( vc ), respiratory rate (f), respiratory exchange ratio (r), ventilatory equivalents for oxygen (e / o2) and carbon dioxide (e / co2), inspiratory time (ti), expiratory time (te), and ti / ttot ratio

Verification of functional capacity by the ergospirometric test in the variables vo2 peak and ve / co2 before after taai and trc and control group.

- - 1. SECONDARY OUTCOME

Analysis and comparison of autonomic modulation in patients with HF before and after TAAI and CRT using linear heart rate variability indices.

Analysis and comparison of body composition in patients with HF before and after TAAI and CRT by densitometry in the parameters% of body fat, lean mass (kg) and adipose mass (kg).

Characterization of the variation in muscle thickness and echo intensity by ultrasonography in patients with HF before and after TAAI and CRT.

Evaluation of the quality of life responses of patients with HF by the classification of states of functionality and disability (ICF) before and after interventions and control group.

Analysis of the effects of TAAI and CRT on handgrip strength by handgrip expressed in kilograms

/ strength

Analysis and functional physical capacity in patients with HF before and after interventions and control group by the short physical performance battery (short physical vattery performance).

Analysis of the effects of TAAI and CRT on peripheral muscle strength using the test of 1 maximum repetition (1RM)

Assessment of isokinetic muscle strength and peripheral oxygen extraction 7 BIBLIOGRAPHIC REFERENCES

1. Ponikowski P, Voors AA, Anker SD, Bueno H, Cleland JGF, Coats AJS, et al. 2016 ESC Guidelines for the diagnosis and treatment of acute and chronic heart failure The Task Force for the diagnosis and treatment of acute and chronic heart failure of the European Society of Cardiology (ESC) Developed with the special contribution. Eur J Heart Fail. 2016; 18 (8): 891–975.
2. Reibis R, Salzwedel A, Buhlert H, Wegscheider K, Eichler S, Völler H. Impact of training methods and patient characteristics on exercise capacity in patients in cardiovascular rehabilitation. Eur J Prev Cardiol [Internet]. 2016; 23 (5): 452–9. Available from: <http://www.ncbi.nlm.nih.gov/pubmed/26285771>
3. Xie B, Yan X, Cai X, Li J. Effects of High-Intensity Interval Training on Aerobic Capacity in Cardiac Patients: A Systematic Review with Meta-Analysis. Biomed Res Int. 2017; 2017.
4. Conraads VM, Pattyn N, De Maeyer C, Beckers PJ, Coeckelberghs E, Cornelissen VA, et al. Aerobic interval training and continuous training equally improve aerobic exercise capacity in patients with coronary artery disease: The SAINTEX-CAD study. Int J Cardiol [Internet]. 2015; 179: 203–10. Available from: <http://dx.doi.org/10.1016/j.ijcard.2014.10.155>
5. Ellingsen Ø, Halle M, Conraads V, Delagardelle C, Larsen A, Hole T, et al. High Intensity Interval Training in Heart Failure Patients with Reduced Ejection Fraction. Circulation. 2017; 136 (23): 1–7.
6. Suchy C, Massen L, Rognmo O, Van Craenenbroeck EM, Beckers P, Kraigher-Krainer E, et al. Optimizing exercise training in prevention and treatment of diastolic heart failure (OptimEx- CLIN): rationale and design of a prospective, randomized, controlled trial. Eur J Prev Cardiol. 2014 Nov; 21 (2 Suppl): 18–25.
7. Meka N, Katragadda S, Cherian B, Arora RR. Review: Endurance exercise and resistance training in cardiovascular disease. Ther Adv Cardiovasc Dis [Internet]. 2008; 2 (2): 115–21. Available from: <http://journals.sagepub.com/doi/10.1177/1753944708089701>
8. Polito MD, Farinatti PTV. Heart rate, blood pressure and dual-product responses to counter- resistance exercise: a literature review. Rev Port Sciences of the Despòorto. 2003; 3 (1): 79–91.
9. Kelemen MH, Stewart KJ, Gillilan RE, Ewart CK, Valenti SA, Manley JD, et al. Circuit weight training in cardiac patients. J Am Coll Cardiol. 1986; 7 (1): 38–42.
10. Hare DL, Ryan TM, Selig SE, Pellizzer a M, Wrigley TV, Krum H. Resistance exercise training increases muscle strength, endurance, and blood flow in patients with chronic heart failure. Am J Cardiol. 1999; 83 (12): 1674–7, A7.
11. Williams AD, Carey MF, Selig S, Hayes A, Krum H, Patterson J, et al. Circuit Resistance Training in Chronic Heart Failure Improves Skeletal Muscle Mitochondrial ATP Production Rate- A Randomized Controlled Trial. J Card Fail. 2007; 13 (2): 79–85.
12. Maupoint J, Besnier M, Gomez E, Bouhzam N, Henry JP, Boyer O, et al. Selective vascular endothelial protection reduces cardiac dysfunction in chronic heart failure. Circ Hear Fail. 2016; 9 (4).
13. Paulus WJ, Tschöpe C. A novel paradigm for heart failure with preserved ejection fraction: Comorbidities drive myocardial dysfunction and remodeling through coronary microvascular endothelial inflammation. J Am Coll Cardiol [Internet]. 2013; 62 (4): 263–71. Available from: <http://dx.doi.org/10.1016/j.jacc.2013.02.092>
14. Sandri M, Viehmann M, Adams V, Rabald K, Mangner N, Höllriegel R, et al. Chronic heart failure and aging - effects of exercise training on endothelial function and mechanisms of endothelial regeneration: Results from the Leipzig Exercise Intervention in Chronic heart failure and Aging (LEICA) study. Eur J Prev Cardiol [Internet]. 2016; 23 (4): 349–58. Available from: <http://journals.sagepub.com/doi/10.1177/2047487315588391>
15. Gutiérrez E, Flammer AJ, Lerman LO, Elízaga J, Lerman A, Francisco FA. Endothelial dysfunction over the course of coronary artery disease. Eur Heart J. 2013; 34 (41): 3175–81.
16. Katz SD, Hryniewicz K, Hriljac I, Balidemaj K, Dimayuga C, Hudaihed A, et al. Vascular endothelial dysfunction and mortality risk in patients with chronic heart failure. Circulation. 2005 Jan; 111 (3): 310–4.
17. Engelen M, Porszasz J, Riley M, Wasserman K, Maehara K, Barstow TJ. Effects of hypoxic hypoxia on O2 uptake and heart rate kinetics during heavy exercise. J Appl Physiol. 1996; 81 (6): 2500–8.
18. Stirling JR, Zakynthinaki M. Counterpoint: The kinetics of oxygen uptake during muscular exercise do not manifest time-delayed phases. J Appl Physiol [Internet]. 2009; 107 (5): 1665–7. Available from: <http://jap.physiology.org/cgi/doi/10.1152/japplphysiol.00158.2009a>
19. Poole DC, Richardson RS, Haykowsky MJ, Hirai DM, Musch TI. Exercise Limitations in Heart Failure with Reduced and Preserved Ejection Fraction. J Appl Physiol [Internet]. 2017; (October):

jap.00747.2017. Available from: <http://jap.physiology.org/lookup/doi/10.1152/japplphysiol.00747.2017>

1. Thijssen DHJ, Black MA, Pyke KE, Padilla J, Atkinson G, Harris RA, et al. Assessment of flow-mediated dilation in humans: a methodological and physiological guideline. AJP Hear Circ Physiol [Internet]. 2011; 300 (1): H2–12. Available from: <http://ajpheart.physiology.org/cgi/doi/10.1152/ajpheart.00471.2010>
2. Restaino RM, Holwerda SW, Credeur DP, Fadel PJ, Padilla J. Impact of prolonged sitting on lower and upper limb micro- and macrovascular dilator function. Exp Physiol [Internet]. 2015; 100 (7): 829–38. Available from: <http://doi.wiley.com/10.1113/EP085238>
3. Boyle LJ, Credeur DP, Jenkins NT, Padilla J, Leidy HJ, Thyfault JP, et al. Impact of reduced daily physical activity on conduit artery flow-mediated dilation and circulating endothelial microparticles. J Appl Physiol [Internet]. 2013; 115 (10): 1519–25. Available from: <http://jap.physiology.org/cgi/doi/10.1152/japplphysiol.00837.2013>
4. Padilla J, Sheldon RD, Sitar DM, Newcomer SC. Impact of acute exposure to increased hydrostatic pressure and reduced shear rate on conduit artery endothelial function: a limb-specific response. AJP Hear Circ Physiol [Internet]. 2009; 297 (3): H1103–8. Available from: <http://ajpheart.physiology.org/cgi/doi/10.1152/ajpheart.00167.2009>
5. Padilla J, Johnson BD, Newcomer SC, Wilhite DP, Mickleborough TD, Fly AD, et al. Adjusting flow-mediated dilation for shear stress stimulus allows demonstration of endothelial dysfunction in a population with moderate cardiovascular risk. J Vasc Res. 2009; 46 (6): 592–600.
6. Cornelis J, Beckers P, Taeymans J, Vrints C, Vissers D. Comparing exercise training modalities in heart failure: A systematic review and meta-analysis. Int J Cardiol [Internet]. 2016; 221: 867–

76. Available from: <http://dx.doi.org/10.1016/j.ijcard.2016.07.105>

1. by Rezende Barbosa MP da C, Silva NT da, by Azevedo FM, Pastre CM, Vanderlei LCM. Comparison of Polar ?? RS800G3 ??? heart rate monitor with Polar ?? S810i ??? and electrocardiogram to obtain the series of RR intervals and analysis of heart rate variability at rest. Clin Physiol Funct Imaging. 2016; 36 (2): 112–7.
2. Vanderlei LCM, Pastre CM, Hoshi RA, Carvalho TD De, Godoy MF De. Basic notions of heart rate variability and its clinical applicability. Rev Bras Cir Cardiovasc. 2009; 24 (2): 205–17.
3. Dias de Carvalho T, Marcelo Pastre C, Claudino Rossi R, by Abreu LC, Valenti VE, Marques Vanderlei LC. Geometric index of heart rate variability in chronic obstructive pulmonary disease. Rev Port Pneumol (English Ed [Internet]. 2011; 17 (6): 260–5. Available from: <http://www.sciencedirect.com/science/article/pii/S2173511511000467>
4. Tillquist M, Kutsogiannis DJ, Wischmeyer PE, Kummerlen C, Leung R, Stollery D, et al. Bedside Ultrasound Is a Practical and Reliable Measurement Tool for Assessing Quadriceps Muscle Layer Thickness. J Parenter Enter Nutr [Internet]. 2014; 38 (7): 886–90. Available from: <http://journals.sagepub.com/doi/10.1177/0148607113501327>
5. Abràmofff MD, Magalhães PJ, Ram SJ. Image processing with ImageJ Part II. Biophotonics Int. 2005; 11 (7): 36–43.
6. Sarwal A, Parry SM, Berry MJ, Hsu FC, Lewis MT, Justus NW, et al. Interobserver Reliability of Quantitative Muscle Sonographic Analysis in the Critically Ill Population. J Ultrasound Med [Internet]. 2015; 34 (7): 1191–200. Available from: <http://doi.wiley.com/10.7863/ultra.34.7.1191>
7. Pillen S, van Keimpema M, Nievelstein RAJ, Verrips A, van Kruijsbergen-Raijmann W, Zwarts MJ. Skeletal muscle ultrasonography: Visual versus quantitative evaluation. Ultrasound Med Biol. 2006; 32 (9): 1315–21.
8. Martins EF, Mara S, Fracon JDF, Sá C De. A Rtigos O Riginais Experience in the Combined Use of International Classifications to Describe Health Information Experience in the Combined Utilization of the International Classifications To Describe Health Information. 2010; 19–27.
9. American Society of Hand Therapists. Clinical assessment recommendations. 2nd ed. Chicago (401 N. Michigan Ave., Chicago IL 60611-4267): The Society © 1992, editor. 1992.
10. Freire AN, Guerra RO, Alvarado B, Guralnik JM, Zunzunegui MV. Validity and Reliability of the Short Physical Performance Battery in Two Diverse Older Adult Populations in Quebec and Brazil. J Aging Health [Internet]. 2012; 24 (5): 863–78. Available from: <http://journals.sagepub.com/doi/10.1177/0898264312438551>
11. Miller MR, Hankinson J, Brusasco V, Burgos F, Casaburi R, Coates A, Crapo R, Enright P, van der Grinten CP, Gustafsson P, Jensen R, Johnson DC, MacIntyre N, McKay R, Navajas D, Pedersen

OF, Pellegrino R, Viegi G WJATF. Standardization of spirometry. Eur Respir J. 2005; 26 (2): 319- 38.

1. Leong DP, Teo KK, Rangarajan S, Lopez-Jaramillo P, Avezum A Jr, Orlandini A, Seron P, Ahmed SH, Rosengren A, Kelishadi R, Rahman O, Swaminathan S, Iqbal R, Gupta R, Lear SA, Oguz A, Yusoff K, Zatonska K, Chifamba J, Igumbor E, Mohan V, Anjana RM, Gu H, Li YS. Prognostic value of grip strength: findings from the Prospective Urban Rural Epidemiology (PURE) study. Lancet. 2015; 18 (386 (9990)): 266–73.
2. Izawa KP, Watanabe S, Oka K, Hiraki K, Morio Y, Kasahara Y, Watanabe Y, Katata H, Osada N OK. Upper and Lower Extremity Muscle Strength Levels Associated With an Exercise Capacity of 5 Metabolic Equivalents in Male Patients With Heart Failure. J Cardiopulm Rehabil Prev. 2012; 32 (2): 85–91.
3. Ponikowski P, Voors AA, Anker SD, Bueno H, Cleland JGF, Coats AJS, et al. 2016 ESC Guidelines for the diagnosis and treatment of acute and chronic heart failure. Eur Heart J. 2016; 37 (27): 2129-2200m.
4. Chronic TTF for the diagnosis and treatment of acute and, (ESC) heart failure of the ES of C. 2016 ESC Guidelines for the diagnosis and treatment of acute and chronic heart failure. Eur Hear J (. 2016; 37: 2129–2200.
5. Nagueh SF, Smiseth OA, Appleton CP, Byrd BF, Dokainish HE, T et al. Recommendations for the evaluation of left ventricular diastolic function by echocardiography: na update from American Society of Echocardiography and the European Association ofn Cardiovascular Imaging. J Am Soc Echocardiogr. 2016; 29 (4): 277–314.
6. Lang RM, Badano LP, Mor-Avi V, Afilalo J, Armstrong A, Ernande L et al. Recommendations for cardiac chambre quantification by echocardiography in adults: na update from American Society of Echocardiography and the European Association of Cardiovascular Imaging. J Am Soc Echocardiogr. 2015; 28 (1): 1–39.

**Onderzoeksprotocol**

# Effects of High Intensity Interval Training versus Circuit Resistance Training on Endothelial Function and Cardiorespiratory Capacity in Patients with Heart Failure:A Randomized Trial

**Principal investigator:** Dominique Hansen, PhD, FESC;

**Representative researchers**: Natália Turri da Silva, Msc; Kenneth Verboven, PhD

# Study protocol (version December 2019)

## INTRODUCTION

Exercise physical training is strongly recommended as a therapeutic approach to treat individuals with Heart Failure (HF), an extremely prevalent cardiovascular disease impacting on patient’s life and society. The present research proposal aims to verify the repercussion exercise training modalities high-intensity intervaltraining (HIIT) and circuit resistance training (CRT) in HF. Findings of those modalities have already indicatedimprovements in HF status, mainly by increasing cardiorespiratory capacity. However, the mechanisms involved to increase functional capacity are not fully understood in the scientific literature, especially over endothelial function and oxygen peripheral extraction. Improvements in those outcomes can impact the quality of life and prognosis of HF individuals positively, by improving dyspnea and fatigue symptoms, increasing exercise tolerance. In order to better understand the physiological pathways responsible for the benefits of HIIT and CRT physical training, we aim to explore the training repercussions over endothelial function (FE) and cardiorespiratory exercise capacity (CPET). Improved EF and increased oxygen supply may result in improved vascular flow and peripheral oxygen extraction to peripheral musculature, justifying the benefits currently described in this population. Objective: To analyze and compare HIIT and CRT exercise modalities in endothelial function and cardiorespiratory capacity in patients with preserved and reduced HF. Methods: It is a multicenter randomized controlled trial involving patients with reduced and preserved HF, divided into HIIT, CRT and control groups (CG). Endothelial function and cardiorespiratory exercise capacity evaluations will occur on pre and post moments following standard recommendations. We hypothesized thatthere is no difference between HIIT and CRT for both outcomes based on interval training characteristic of both modalities, alternating pauses (CRT) or intensity reductions (HIIT) with higher exercise efforts impacting on intensity control. Oxidative metabolism is mainly required during HIIT to provide energy supply, positivelyinfluencing FMD and CPET, while for CRT those benefits can be related to cardiovascular demand and muscular alterations.

## PURPOSES

- - To analyze and compare the effects of HIIT and CRT on endothelial function and cardiorespiratoryexercise capacity in patients with HF.
  - To analyze and compare the effects of these modalities on skeletal muscle strength, muscle quality,body composition, pulmonary function, autonomic modulation and functional capacity.

## METHODS

Trial design

This multicentric randomized controlled trial is designed as longitudinal, parallel, and quantitative study following CONSORT recommendations (Consolidated Standards for Reporting) (1).

Group determination and study content

Heart failure patients will be recruited during the cardiology consultation. Suitable patients (based on the decision of the cardiologist) will be introduced the content of the study by the researcher, after which the patient receives the informed consent. The patient will be called one week later to determine whether he/she is willing to participate in the study. If the patient will not participate in the study, his/her contact details willbe removed after this phone call. Heart Failure patients will be allocated in three randomized (by closed envelope) groups: high-intensity interval training (HIIT), circuit- resistance training (CRT), and control group without exercise intervention (CG). Both training groups will perform a 12 week intervention, personally supervised by a team of physiotherapists and biomedical scientists. Patients in the control group will get thestandard clinical treatment trajectory by their cardiologist, after which these patients will be invited to take part in the training intervention. All measurements will be performed at the start of the intervention and willbe repeated after the intervention period of 12 weeks (or the follow-up period of the control group). There will be two measurement days: one at the Jessa Hospital (Revalidatie- en Gezondheidscentrum) and one at Hasselt University (Revalidatie Onderzoekscentrum). Both occasions will comprise about 2-3 hours of testing.Each patient will receive an instruction card which includes the most important guidelines to take into account the days before the measurements. Instructions for the measurements are given at the measurement days.

Population

For this study, only stable heart failure patients (no changes in medication throughout the last three months;decision to be suitable for inclusion is based on cardiologists’ opinion) with the following criteria can participate:

- Inclusion criteria

Individuals with HF diagnosis with reduced and preserved ejection fraction (HFrEF and HFpEF, respectively) according to 2016 ESC Guidelines (2), referred by doctors, both sex, older than 30 years, who did not participate in a previous exercise program six months before the protocol, non-smokers, non-Chagas disease, absence of exercise respiratory limitations according to spirometry (FEV1<50%)(3), absence of inflammatoryor infectious processes, absence of musculotendinous or osteoarticular lesions which could limit exercise performance.

- Exclusion criteria

Individuals who disagree participate to the research protocol. The follow up exclusion will occur for thoseabstaining more than 25% of the 36 training sessions, or more than three consecutive training sessions.

Outcomes

*Primary*: 1) flow-mediated vasodilation (FMD) and 2) cardiorespiratory exercise capacity (CPET).

*Secondary*: 3) muscular strength, 4) body composition, 5) pulmonary function, 6) autonomic modulation, 7)Muscle Quality 8) Short Physical Performance Battery.


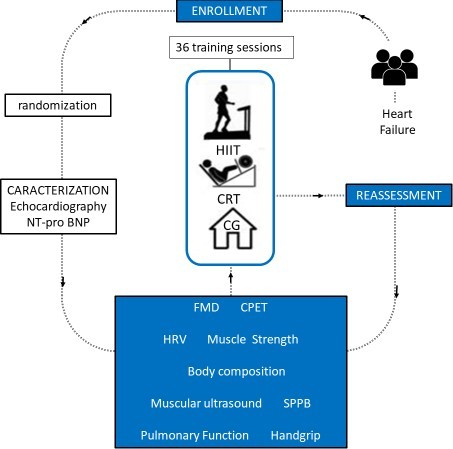
*Sample characterization only*: 9) cardiac function (echocardiography), 10) NT-proBNP

Figure 1: Study overview flowchart. HIIT, high-intensity interval training; CRT, circuit-resistance training; CG, control group; NT-pro BNP, brain natriuretic peptide; FMD, flow-mediated dilatation; CPET, cardiopulmonary exercise test; HRV, heart rate variability; M., muscular; DXA, dual-emission x-ray absorptiometry; SPPB, Short Physical Performance Battery.

## FLOW-MEDIATED DILATION (FMD)

Flow-mediated dilation (FMD) is a non-invasive technique to evaluate endothelial function (9) and has beenconsidered an important prognostic variable for this population (10). The technique allows the visualization of Doppler waveform associated with arterial diameter measurements (11,12). Brachial endothelial dysfunction is associated with carotid thickness (13).

Subjects preparation: Subjects will start the experimental procedure at least 2 h after a light meal and abstained from caffeine, alcohol, and exercise for 24 h before testing. Tests will be performed at the same period of the day to avoid the circadian effect, and temperature will be controlled (~ 24° C / 75° F). Subjectspreparation follows a previous standard recommendation (11).

Method Description: A cuff will be attached to the right arm of the subjects who will remain at rest supine position for 5 min for cardiovascular stabilization. Later, diameter and blood velocity of the brachial artery will be continuously measured by two minutes, according to Thijssen et al. 2011 (6), using high-resolution

Doppler duplex ultrasound equipment in Brazil (HD11.XZ, 1 and 3 MHZ, Phillips, Barueri, SP, Brazil) and at UHasselt (E95 Vivid, GE Healthcare, EUA)(12)(12)(8)(8). A 9 MHz linear matrix transducer will be positioned over the brachial artery discretely proximal to cubital fossa. Diameter and velocity signals will be obtained simultaneously in duplex mode at a pulse frequency of 5 MHz and corrected by an insonation angle of 60°. Sample volume will be adjusted to include the entire vessel's lumen without extending beyond the walls, andthe slider will be adjusted in the middle of the vessel. The FMD of the brachial artery will be evaluated on theright arm in the supine position, as previously described (14,15). After resting period evaluation, the cuff willbe inflated to a pressure of 220 mmHg and maintained for 5 minutes. After that, measurements of the diameter, and blood flow velocity will be recorded continuously for 3 min after rapid cuff deflation. All vascular variables will be obtained by using specialized border detection software (Cardiovascular Suite, Quipu, Pisa, Italy). Percentage variation of FMD will be normalized (16). Baseline arterial Diameter, baseline blood velocity, temporal kinetics of arterial diameters, and blood velocities will be considered for analysis(11,17).

## CARDIORESPIRATORY EXERCISE TEST

Cardiorespiratory exercise capacity will be obtained by the cardiorespiratory exercise test (CPX) within an incremental symptom-limited test on a 0-watt electro-magnetic bicycle (Corival, LODE BV Medical Technology Groningen - Netherlands) under a specialized physician supervision, blinded to allocation.

Subjects preparation: The subjects will be instructed to wear comfortable clothes without movement restriction and instructed to abstain from caffeine for 24h before testing and have a light meal until 2 hours earlier.

Method description: A breath-by-breath gas analyzer (CPET, Cosmed, Rome, Italy) will be used. This examination will determine ventilatory thresholds (18) for HIIT prescription (heart rate at the aerobic threshold and respiratory compensation point). Outcome measures including VO2, VCO2, HR, and VE and allkey variables for cardiorespiratory capacity assessment (19) will be determined.

## MUSCLE STRENGTH

Muscle strength will be assessed through:

1. Isokinetic dynamometer;
2. 1 RM;
3. Palmar hand grip.

For muscle strength assessment patients will be oriented to use a comfort short and perform the proceduresat least 2 hours after a light meal, and abstain from exercise for 24 hours prior to testing.

- 1. *ISOKINETIC DYNAMOMETER*

Subjects preparation: Patients will be instructed to adopt a seated position (90° hip) in a good posture. Beltswill be used to stabilize thigh, pelvis, and trunk. Joint axis will be aligned with the dynamometer allowing knee expose. Alignment between both legs will be verified. All patient measures and dynamometer position (as chair height, chair base, seat backrest, dynamometer distance, arms distance) will be standardized at baseline and at post protocol evaluation (20).

Method description: Calibration of the equipment will be performed according to the manufacturer’s specifications before every testing session. Patients will be carefully stabilized with Velcro belts, and the rotational axis of the dynamometer arm will be oriented with the lateral condyle of the right participant’s femur. In an angular adjustment of 75° s-1 (stimulating much increase on muscle strength in patients with heart failure) (20–23) the isometric familiarization will be performed. Familiarization consists of three voluntary submaximal contractions, each one with a 5s duration, including 30s of in between rest period, then, the test will be ready to start. The patients will be allowed to perform five contractions (considering 30 s between attempts), and the largest value will be considered for analysis.

After 3 minutes of rest, the patients start isokinetic familiarization by performing six maximal repetitions as fast as possible. Post 3 minutes of recovery, the endurance protocol starts, 20 repetitions at 180 ° s-1, once (24). Peak torque (N-M); peak torque and body weight ratio (%); total repetition maximum work (J); total work (J); fatigue (%) and mean load (W) will be obtained by isokinetic dynamometer (Biodex System 3 PRO, Medical Inc., New York, EUA).

- 1. *1RM TESTING*

Subjects preparation: After checking blood pressure at rest and medication use, patients will initiate the 1RMprotocol.

Method description: Following a brief familiarization, patients will perform five repetitions of a light to moderate load (~50% of predicted 1RM) in order to learn the movement amplitude motion. The patients will be instructed to graduate the load perception as: i) light, ii) light to moderate, iii) moderate, iv) moderate tointense, iv) intense. Familiarization and warm up consisted of 10 repetitions of each exercise load only if referred as light to moderate. Later, the 1RM test starts with increments according to the subject's perception and finish when finding the maximal load in a good posture. After each successful performance, the weight increased until a failed attempt occurred. No more than five attempts will be allowed for each muscular group. The 1RM test will be performed for an

experienced therapist. In case more than five attempts are needed, another test day will be scheduled to finish the test. 1RM will be collected before starting exercise protocol for prescription purposes.

- 1. *PALMAR HAND GRIP STRENGTH*

Palmar handgrip strength has been used to predict cardiovascular events and mortality (25–27).

Subjects preparation: The patient will be seated at rest for palmar grip strength test evaluation using the Digital Hand Dynamometer (Jamar Plus+, Jamar) following the American Society of Hand Therapists Guidelines (28).

Method description: Subject seated with arm supported, shoulders adducted in neutral rotation, elbow flexed at 90°, forearm in the neutral position and wrist between 0 and 30° of dorsiflexion. Three measures with the dominant and non-dominant hands will be performed. The highest value shall be expressed in kg and included for analyzes. The movements should be performed for 5 seconds following 3 minutes of rest after each attempt. Patients will be instructed to maintain spontaneous breathing and posture alignment. Accessories such as watches, rings, and bracelets will be removed from both participants' upper limbs prior to the test. Participants will be instructed not to look at the dynamometer display to avoid any visual feedback(28).

## BODY COMPOSITION

Whole body composition will be estimated by dual-emission x-ray absorptiometry (DXA - Lunar Prodigy BoneDensitometers, GE Healthcare, USA) (29,30). a) Fat mass (MG), b) lean mass (MLG), and c) bone mass will beexpressed in absolute values and percentages.

Subjects preparation: The subjects will be instructed to wear gym clothes and remove all metal belongs(bracelets, rings, earring, watch) before the exam. Individuals with pacemakers are not eligible for the test. Method description: All subjects will have their weight and height measured before to adopt a supineposition on DXA. During the exam, the patients will be stationary for 10 minutes average after assuming theadjusted position.

## PULMONARY FUNCTION

Pulmonary function and respiratory muscle strength will be accessed by spirometry (MicroLab ML3500MK8,CareFusion, EUA) and manovacuometer (MVD300, Globalmed, Brazil) tests.

Subjects preparation: Subjects will be instructed to abstain from autonomic stimulants (alcoholic drinks, coffee, tea or food containing caffeine) during 24h preceding the evaluation, as well as to ingest a light mealat least 2h before measurement. Patients shall rest 10 minutes before each test (spirometry, maximum respiratory pressures, and dynamic inspiratory pressure). They will then be placed sitting on a bench with their legs bent 90 degrees, erect posture, head in a neutral position, and nasal clip placed to prevent leakage(31).

- 1. *SPIROMETRY*

Method Description: Individuals will perform at least three forced expiration maneuvers according to the technical procedures and criteria of acceptability and reproducibility of American Thoracic Society (ATS) / European Respiratory Society (ERS)(31). Spirometry evaluation will be performed to check obstructive or restrictive patterns through outcome measures: forced expiratory volume in 1 second (FEV1), forced vital capacity (FVC), expiratory peak flow (EPF), FEV1/FVC ratio.

- 1. *MANOVACUOMETER*

The MVD300® manovacuometer (Globalmed, Porto Alegre, RS, Brazil) was utilized to measure the positive pressures (manometer) and negative pressures (vacuometer). Manovacuometer allows the static evaluation of maximum inspiratory pressure (MIP) and the maximum expiratory pressure (MEP) and play an importantrole in the diagnosis and prognosis of chronic diseases (25).

Method Description: The manovacuometer will be previously calibrated in cmH2O, with an operational limitof -300 to +300 cmH2O and scales ranging from 10 to 10 cmH2O. The methods and criteria used will be thoserecommended by ATS / ERS (24). To assess the MIP, patients will be instructed to perform a maximal expiration, following a maximal inspiratory effort so the pressure will be recorded close to the residual volume. For the assessment of MEP, a maximum inspiration will be requested before the maximal expiratoryeffort to evaluate the pressure, close to total lung capacity. A vigorous verbal command will be given duringthe evaluation.

- 1. *DYNAMIC INSPIRATORY PRESSURE*

Method Description: The evaluation of the dynamic respiratory muscle strength will be performed using thePOWERbreathe® KH2 device (London, England, UK), in which the maximal inspiratory effort will be assessed from residual volume. The maximum muscle strength (S-Index) will be obtained during the dynamic contraction of the inspiratory muscles. Patients will be instructed to achieve the highest possible inspiratoryflow rate after a previous expiration. In order to obtain the S- index, the patient must be collaborative and able to respond to verbal commands (24).

## HEART RATE VARIABILITY

Autonomic modulation will be accessed by heart rate variability (HRV) method using heart rate monitor (Polar® RS800, Polar Electro OY, Finland) following linear index analysis in the time and frequency domains (56).

Subjects preparation: Subjects will be instructed to abstain from autonomic stimulants (alcoholic drinks, coffee, tea or food containing caffeine) during 24h preceding the evaluation, as well as to ingest a light mealat least 2h before measurement. In order to reduce anxiety during HRV evaluation, the volunteers will be kept alone in a comfort, quiet, and climate room. Subjects will be oriented to keep in silence and awake, at rest, breathing spontaneously for 30 minutes in the supine position during evaluation by Polar RS800 monitor(Polar Electro®, Finland).

Method description: HRV data acquisition will be conducted in the morning to prevent circadian changes. The room temperature will be controlled (21 to 23º C / 70 to 73º F) and relative air humidity between 40% and 60%. Only period contained more than 95% sino-atrial node beats will be recorded and analyzed in the study. Stationary frames of 1000 R-R intervals (RRi) will be selected, according to the most stable signal of data acquisition. The Kubios software will be used to run HRV analysis (Biosignal Analysis and Medical Image Group, Department of Physics, University of Kuopio, Finland)(32).

Time domain analysis includes mean RR intervals (reflecting global variability); the square root of the mean squared difference between adjacent RR intervals (RMSSD) - reflecting parasympathetic modulations of HR; the standard deviation of all normal RR intervals (SDNN) - reflecting global variability. The geometric indexesfrom Poincaré plot (SD1 and SD2, reflecting parasympathetic and global modulations of HR, respectively);

triangular interpolation of normal to normal RR intervals (TINN) and triangular index (RRtri) both reflecting global variability. (33,34)

For the frequency domain, low frequency (LF: between 0.04 and 0.15Hz), high frequency (HF: between 0.15to 0.4Hz) and the relationship LF/HF will be computed. The spectral analysis includes Fast Fourier Transformcalculations. Spectral indexes will be expressed in absolute units (ms2) and normalized units (HFnu and LFnu).The power in the LF band is modulated by both the sympathetic and the parasympathetic branches of the autonomic nervous system and the power in the HF band is correlated with vagal modulation (33,34)

## MUSCLE QUALITY ASSESSMENT

Subjects preparation: Patients will be previously oriented to wear shorts.

Method description: Ultrasound imaging will be captured using ultrasound (HD11XE, Phillips, Amsterdam, The Netherlands) with 7.5-MHz linear array transducers.

Individuals will be evaluated in the supine position with the knee in passive extension and neutral rotation. Water-gel will be applied to the ultrasound transducer to allow acoustic contact without pressing the dermalsurface. The images will be acquired in the right leg (rectus femoris - knee at 45 °), with the transducer placedtransversely and perpendicular to the long axis of the anterior thigh (rectus femoris and vastus lateralis: 50%of the distance between the iliac spine anterior superior to upper anterior patellar border, anterior tibial: 25% of distance between medial condyle of tibia to lateral malleolus (35). The images will be saved on the ultrasound hard disk and transferred for later analysis on a computer using ImageJ software (bundled with 64- bit Java 1.8.0_112, 70 MB, NIH, Bethesda, USA) (36).

All ultrasound measurements will be performed three times, using the mean of the scores for the final analyses. According to the images, the muscular thickness, echo intensity (minimum, maximum and average of the grayscale) and thickness of the subcutaneous tissue will be evaluated. All thickness parameters will bemeasured in centimeters.

The echo intensity will be reported in pixels. The echo intensity will be determined using computer quantitative scales. A 2 × 2cm square pattern for analysis of the rectus femoris and vastus lateralis will be used to determine the ROI. The square method has stronger confidence compared to the tracking method (where the evaluator highlights the entire visible muscle area, excluding epimysium and artifacts) to define ROI80 (37). If the area to be analyzed is smaller than 2 × 2 cm, the largest possible square within the anatomical limits of the muscle will be examined. Mean, and standard deviation of the echo intensity of thisROI will be calculated using the ImageJ software histogram function (bundled with 64-bit Java 1.8.0_112, 70MB, NIH, Bethesda, USA) and expressed as a value between 0 (black) and 255 (white)(38).

## SHORT PHYSICAL PERFORMANCE BATTERY

The Short Physical Performance Battery (SPPB), is considered a brief performance battery based on short distance walking, repeated chair stands, and a set of balance tests (39). SPPB is a validated assessment tool able to measure lower extremity function which has been widely used in both clinical and research settings (40,41). Mainly used in aging studies, low scores in the SPPB is associated with a wide range of health outcomes such as hospitalization, length of hospital stay, mobility loss, disability, nursing home admission, and death (42,43).

Subjects preparation: Patients will be previously oriented to wear sports clothes.

Method description: SPPB execution will follow previous recommendations (39). Firstly, patients will performa static equilibrium test based on three different positions held for 10 seconds: side by side (parallel feet); semi-tandem (one foot partially in front of the other) and tandem (one foot positioned in front of the other).The maximum total score possible for equilibrium tests is 4 points, in which two points are attributable to the last task.

Further, patients will perform twice the walking speed test, considering the time spent to walk along three meters corridor in their usual step. The shortest time will be considered for analysis. Scores rates as follow: if the walking time is less than 4.82 seconds = 4 points; between 4.82 and 6.20 seconds = 3 points; between 6,21 and 8,70 = 2 points; greater than 8.70 = 1 point. If the patient does not perform the walk, so no punctuation is awarded.

Finally, the third test of the battery evaluates lower limbs strength (the time spent to stand up and sit downon a chair for five consecutive times, without the aid of hands). Patients must perform five

consecutive attempts without using upper limbs. The maximum score assigned is 4 points for a test time of 11.19 secondsor less; 3 points for a time of 11.20 to 13.69 seconds; 2 points for a test time of

13.70 to 16.69 seconds and

1 point for 16.70 seconds or more. If patients are unable to perform the test within 60 seconds, or if the patient is not able to get up from the chair five times, the score assigned is zero.

The total score of SPPB is a result of the sum of equilibrium, walking speed and lower limb strength, totalizing12 points. Scores from 0 to 3 points are considered incapacity or very poor performance, scores from 4 to 6 points are considered low performance; from 7 to 9 points means moderate performance and from 10 to 12points are considered good performance.

## TRAINING INTERVENTION PROTOCOL

Training protocols will occur three times per week during 36 sessions with a matched session duration (≈40 minutes). Training familiarization for both modalities will be established for the patient's adaptation, with a duration of 6 sessions. In case a patient requires more than 6 familiarization sessions to reach the adequateHIIT or CRT protocol intensities, the number of familiarization sessions will be recorded for further analysis. The exercises on the CRT group will be conducted on six large muscle groups, according to the established sequence: pull down, leg press, pectoralis machine, flexor chair, shoulder press, and extensor chair machines.Before the CRT sessions, 10 minutes of warming up will be guided by the therapist, 5 minutes focusing on muscle stretching, and 5 minutes on dynamic movements to promotes HR and blood flow increase. CRT will be performed in resistive stations, as (EN-Dynamic, Enraf-Nonius, Rotterdam). Exercise order will vary cyclically in each session, but respecting exercise sequence. During the six sessions of familiarization on CRT, training load will be set at 50% of 1RM, with 3 circuit series of 12 repetitions. After the familiarization period, the workloads will be set as 60% 1RMnin the 1st month; 70%1RM in the 2nd monthand 80% 1RM in the 3rd month, following adaptation and overload principles (57). Repetitions: 6 to 12 repetitions in the first two weeks of each month and 15 to 20 in the last two weeks of each month. 3 circuit series of each exercise with 1 min of rest between exercises will be performed.

During the familiarization period on HIIT, the moderate intensity will be performed daily-alternated on a treadmill (T150, COSMED, EUA) and at an ergometric bicycle (Corival, LODE BV Medical Technology Groningen - Netherlands), in which the heart rate should reach values higher than the 1st ventilatory

threshold for 30 minutes. HIIT will be gradually incorporated during familiarization to guarantee an adequateHR response during the research protocol. Before starts HIIT protocol, individuals will perform 10 minutes of warm-up at 10% above the HR equivalent to the 1st ventilatory threshold obtained by previous CPX. HIIT loads should vary between two intensities, 10% above the 1st (low-intensity) and 10% higher the 2nd ventilatory threshold (high-intensity) obtained from CPX. HIIT protocol will be applied by 4 minutes at low intensity followed by 3 minutes at high-intensity protocol, totalizing four cycles of 7 minutes. in 28 minutes of HIIT (44).

Polar Software will continuously register HR monitorization during training sessions.

Results information

Only clinically validated results will be communicated to the patient as well as their corresponding cardiologist.

2

Insurance and costs

Participation in the study will not result in additional costs for the patient. All costs will be handled by the researchers. If any problem occurs throughout the study period, as a result of the study participation, an insurance policy can be consulted.

Data handling and protection

All data will be handled confidentially and coded uniquely. A subject identification list will be used to link data to the subjects. The key to the code will be safeguarded by an independent investigator that is not involved in the study. All collected data will be stored in digital Case Report Forms at the Google Drive File Stream (protected by Hasselt University) and only the responsible researcher will have access to the source data. The participants have the right to ask the researchers for which data are being collected and what is the purpose of these obtained data. Participants can always ask to take a look in the obtained data and to correct these in case of necessity. Source data will be stored for 25 years in accordance with GCP standards to re-use them or to validate results. Future research in line with the current study (re-use of the data) will only be conducted with subject’s approval on the informed consent form for re-use of the data and approvalof the new study by the ethical committee.

The obtained personal data will not contain such elements that would make it possible to identify individualpatients, which is in accordance with the EU guidelines (2016/679) and GDPR guidelines concerning personaldata protection. Anonymized research data can, provided that permission has been obtained, only be evaluated by authorized collaborators of the research institute or the ethical committee via the responsible researcher Dominique Hansen.

## STATISTICAL ANALYSIS

Descriptive analysis will be used for sample characterization. Shapiro Wilk test will check data normality. Parametric or non-parametric tests will be applied according to standard or non-standard data distribution (ANOVA two way with Tukey post-test or Kruskal Wallis with Newman Keuls post-test). Possible correlationswill be tested using Spearman or Pearson correlation. Statistical software SPSS version 22.0 (SPSS, Inc.

3

Chicago, IL, USA) will be used following a 5% significance level. Sample size calculation made by Manova repeated measures, between factors from pilot data (n=11) and based on baseline arterial diameter (mm) indicated a minimum total sample size of 15 individuals (α err prob =0,05; power 1-β err prob = 0,95, effect size f = 0,914).

## REFERENCES

1. Schulz KF, Altman DG, Moher D. CONSORT 2010 Statement: updated guidelines for reporting parallel group randomised trials. BMJ [Internet]. 2010 Mar 23;340(mar23 1):c332–c332. Available from: <http://www.bmj.com/cgi/doi/10.1136/bmj.c332>
2. Ponikowski P, Voors AA, Anker SD, Bueno H, Cleland JGF, Coats AJS, et al. 2016 ESC Guidelines for the diagnosis and treatment of acute and chronic heart failure The Task Force for the diagnosis and treatment of acute and chronic heartfailure of the European Society of Cardiology ( ESC

) Developed with the special contribution. Eur J Heart Fail. 2016;18(8):891–975.

1. Pellegrino R. Interpretative strategies for lung function tests. Eur Respir J [Internet]. 2005 Nov 1;26(5):948–68. Available from: <http://erj.ersjournals.com/cgi/doi/10.1183/09031936.05.00035205>
2. Ponikowski P, Voors AA, Anker SD, Bueno H, Cleland JGF, Coats AJS, et al. 2016 ESC Guidelines for the diagnosis and treatment of acute and chronic heart failure. Eur Heart J. 2016;37(27):2129-2200m.
3. Bugge C, Sether EM, Pahle A, Halvorsen S SKI. Diagnosing heart failure with NT-proBNP point-of-care testing: lower costsand better outcomes. A decision analytic study. BJGP. 2018;2:bjgpopen18X101596.

4

1. Otto ME, Pereira MM, Beck AL MM. Correlation between diastolic function and maximal exercise capacity on exercise test. Arq Bras Cardiol. 2011;96(2):107–13.
2. Nagueh SF, Smiseth OA, Appleton CP, Byrd BF, Dokainish H E, T et al. Recommendations for the evaluation of left ventricular diastolic function by echocardiography: na update from American Society of Echocardiography and the European Association ofn Cardiovascular Imaging. J Am Soc Echocardiogr. 2016;29(4):277–314.
3. Lang RM, Badano LP, Mor-Avi V, Afilalo J, Armstrong A, Ernande L et al. Recommendations for cardiac chambre quantification by echocardiography in adults: na update from American Society of Echocardiography and the European Association of Cardiovascular Imaging. J Am Soc Echocardiogr. 2015;28(1):1–39.
4. Celermajer DS, Sorenses KE, Gooch V, Spiegehalter D, Miller O, Sullivan I, et al. Non-invasive detection of endothelial dysfunction in children and adults at risk of atherosclerosis. 1992;(340):1111–5.
5. Matsuzawa Y, Kwon T, Lennon RJ, Lerman LO, Lerman A. Prognostic Value of Flow‐Mediated Vasodilation in Brachial Arteryand Fingertip Artery for Cardiovascular Events: A Systematic Review and Meta‐Analysis. 2015;4(e002270):1–15.
6. Harris RA, Nishiyama SK, Wray DW, Richardson RS. Ultrasound Assessment of Flow-Mediated Dilation. Hypertension. 2010;55:1075–85.
7. Thijssen DHJ, Black MA, Pyke KE, Padilla J, Atkinson G, Harris RA, et al. Assessment of flow-mediated dilation in humans: amethodological and physiological guideline. AJP Hear Circ Physiol [Internet]. 2011;300(1):H2–
   1. Available from:

5

<http://ajpheart.physiology.org/cgi/doi/10.1152/ajpheart.00471.2010>

- 1. Juonala M, Viikari JSA, Laitinen T, Marniemi J, Helenius H, Rönnemaa T, et al. Interrelations Between Brachial Endothelial Function and Carotid Intima-Media Thickness in Young Adults. Circulation [Internet]. 2004 Nov 2;110(18):2918–23. Available from: https[://w](http://www.ahajournals.org/doi/10.1161/01.CIR.0000147540.88559.00)ww[.ahajo](http://www.ahajournals.org/doi/10.1161/01.CIR.0000147540.88559.00)u[rnals.org/doi/10.1161/01.CIR.0000147540.88559.00](http://www.ahajournals.org/doi/10.1161/01.CIR.0000147540.88559.00)
  2. Restaino RM, Holwerda SW, Credeur DP, Fadel PJ, Padilla J. Impact of prolonged sitting on lower and upper limb micro- and macrovascular dilator function. Exp Physiol [Internet]. 2015;100(7):829–38. Available from: <http://doi.wiley.com/10.1113/EP085238>
  3. Boyle LJ, Credeur DP, Jenkins NT, Padilla J, Leidy HJ, Thyfault JP, et al. Impact of reduced daily physical activity on conduit artery flow-mediated dilation and circulating endothelial microparticles. J Appl Physiol [Internet]. 2013;115(10):1519–25. Available from: <http://jap.physiology.org/cgi/doi/10.1152/japplphysiol.00837.2013>
  4. Padilla J, Johnson BD, Newcomer SC, Wilhite DP, Mickleborough TD, Fly AD, et al. Adjusting flow-mediated dilation for shear stress stimulus allows demonstration of endothelial dysfunction in a population with moderate cardiovascular risk. JVasc Res. 2009;46(6):592–600.
  5. Corretti MC, Anderson TJ, Benjamin EJ, Ms C, Celermajer D, Charbonneau F, et al. Guidelines for the Ultrasound Assessment of Endothelial-Dependent Flow-Mediated Vasodilation of the Brachial Artery A Report of the International Brachial Artery Reactivity Task Force. J Am Coll Cardiol [Internet]. 2002;39(2):257–65. Available from: <http://dx.doi.org/10.1016/S0735-1097(01)01746-6>
  6. Beaver WL, Wasserman K WB. A new method for detecting anaerobic

6

threshold by gas exchange. J Appl Physiol (1985). 1986;60(6):2020–7.

- 1. Cornelis J, Beckers P, Taeymans J, Vrints C, Vissers D. Comparing exercise training modalities in heart failure: A systematicreview and meta-analysis. Int J Cardiol [Internet]. 2016;221:867–76. Available from: <http://dx.doi.org/10.1016/j.ijcard.2016.07.105>
  2. Bottaro M. THE EFFECTS OF REST INTERVAL ON QUADRICEPS TORQUE DURING AN ISOKINETIC TESTING PROTOCOL IN ELDERLY. 2005;4:285–90.
  3. Rice AJC and CL. Fatigue and recovery of power and isometric torque following isotonic knee extensions. J Appl Physiol. 2005;99:1446 –1452.
  4. Rozand V, Cattagni T, Theurel J, Martin A LR. Neuromuscular Fatigue Following Isometric Contractions with Similar Torque Time Integral. Int J Sports Med. 2015;36(1):35–40.
  5. Toth MJ, Miller MS, Vanburen P, Bedrin NG, Lewinter MM, Ades PA, et al. Resistance training alters skeletal musclestructure and function in human heart failure : effects at the tissue , cellular and molecular levels. 2012;5:1243–59.
  6. Toth MJ, Shaw AO, Miller MS, VanBuren P, LeWinter MM, Maughan DW, et al. Reduced knee extensor function in heartfailure is not explained by inactivity. Int J Cardiol [Internet]. 2010 Sep;143(3):276–82. Available from: <http://linkinghub.elsevier.com/retrieve/pii/S0167527309002277>
  7. Gubelmann C, Vollenweider P M-VP. Association of grip strength with cardiovascular risk markers. Eur J Prev Cardiol[Internet]. 2017;24(5):514–21. Available from: https[://w](http://www.ncbi.nlm.nih.gov/pubmed/27885059)ww[.n](http://www.ncbi.nlm.nih.gov/pubmed/27885059)c[bi.nlm.nih.gov/pubmed/27885059](http://www.ncbi.nlm.nih.gov/pubmed/27885059)

7

- 1. Izawa KP, Watanabe S, Oka K, Hiraki K, Morio Y, Kasahara Y, Watanabe Y, Katata H, Osada N OK. Upper and Lower Extremity Muscle Strength Levels Associated With an Exercise Capacity of 5 Metabolic Equivalents in Male Patients With Heart Failure. J Cardiopulm Rehabil Prev. 2012;32(2):85–91.
  2. Leong DP, Teo KK, Rangarajan S, Lopez-Jaramillo P, Avezum A Jr, Orlandini A, Seron P, Ahmed SH, Rosengren A, Kelishadi R, Rahman O, Swaminathan S, Iqbal R, Gupta R, Lear SA, Oguz A, Yusoff K, Zatonska K, Chifamba J, Igumbor E, Mohan V, Anjana RM, Gu H, Li YS. Prognostic value of grip strength: findings from the Prospective Urban Rural Epidemiology (PURE) study. Lancet. 2015;18(386(9990)):266–73.
  3. American Society of Hand Therapists. Clinical assessment recommendations. 2nd ed. Chicago (401 N. Michigan Ave., Chicago IL 60611-4267) : The Society ©1992, editor. 1992.
  4. Glickman SG, Marn CS, Supiano MA DD. Validity and reliability of dual- energy X-ray absorptiometry for the assessment ofabdominal adiposity. J Appl Physiol (1985). 2004;97(2):509–14.
  5. Hull H, He Q, Thornton J, Javed F, Allen L, Wang J, Pierson RN Jr GD. iDXA, Prodigy, and DPXL dual-energy X-ray absorptiometry whole-body scans: a cross-calibration study. J Clin Densitom. 2009;12(1):95-102.
  6. Miller MR, Hankinson J, Brusasco V, Burgos F, Casaburi R, Coates A, Crapo R, Enright P, van der Grinten CP, Gustafsson P, Jensen R, Johnson DC, MacIntyre N, McKay R, Navajas D, Pedersen OF, Pellegrino R, Viegi G WJATF. Standardisation of spirometry. Eur Respir J. 2005;26(2):319-38.

8

- 1. Tarvainen MP, Niskanen JP, Lipponen JA, Ranta-aho PO, Karjalainen PA. Kubios HRV - Heart rate variability analysissoftware. Comput Methods Programs Biomed [Internet]. 2014;113(1):210–20. Available from: <http://dx.doi.org/10.1016/j.cmpb.2013.07.024>
  2. Vanderlei LCM, Pastre CM, Hoshi RA, Carvalho TD De, Godoy MF De. Basic notions of heart rate variability and its clinicalapplicability. Rev Bras Cir Cardiovasc. 2009;24(2):205–17.
  3. Electrophysiology TF of the ES of C the NAS of P. Heart Rate Variability Standards of Measurement, Physiological Interpretation, and Clinical Use. Circulation [Internet]. 1996;93(1):1043–65. Available from: https://doi.org/10.1161/01.CIR.93.5.1043
  4. Tillquist M, Kutsogiannis DJ, Wischmeyer PE, KummTillquist M, Kutsogiannis DJ, Wischmeyer PE, Kummerlen C, Leung R5, Stollery D, Karvellas CJ, Preiser JC, Bird N, Kozar R, Heyland DKerlen C, Leung R, Stollery D, Karvellas CJ, Preiser JC, Bird N, Kozar R HD. Bedside Ultrasound Is a Practical and Reliable Measurement Tool for Assessing Quadriceps Muscle Layer Thickness. J Parenter Enter Nutr. 2013;38(7):886– 90.
  5. Abràmofff MD, Magalhães PJ, Ram SJ. Image processing with ImageJ Part II. Biophotonics Int. 2005;11(7):36–43.
  6. Arts IMP, Phillens S, Schelhaas HJ, Overeem S ZM. Normal values for quantitative muscle ultrasonography in adults. Muscle Nerve. 2010;41(1):32– 41.
  7. Pillen S, van Keimpema M, Nievelstein RAJ, Verrips A, van Kruijsbergen- Raijmann W ZM. Skeletal muscle ultrasonography: Visual versus quantitative evaluation. Ultrasound Med Biol. 2006;32(9):1315–21.

9

- 1. Guralnik JM, Simonsick EM, Luigi Ferrucci RJG, Berkman LF, Blazer DG, Paul A. Scherr, et al. A Short Physical Performance Battery Assessing Lower Extremity Function: Association With Self-Reported Disability and Prediction of Mortality and Nursing Home Admission. J Gerontol Med Sci. 1994;49(2):M85–94.
  2. Tomohiro Yasuda, Toshiaki Nakajima, Tatsuya Sawaguchi, Naohiro Nozawa T, Arakawa, Reiko Takahashi, Yuta Mizushima, Satoshi Katayanagi KM, Inoue ST& T. Short Physical Performance Battery for cardiovascular disease inpatients: implications for critical factors and sarcopenia. Sci Rep. 2017;7(1):17425.
  3. Freire AN, Guerra RO, Alvarado B, Guralnik JM, Zunzunegui MV. Validity and Reliability of the Short Physical Performance Battery in Two Diverse Older Adult Populations in Quebec and Brazil. J Aging Health [Internet]. 2012;24(5):863–78. Available from: <http://journals.sagepub.com/doi/10.1177/0898264312438551>
  4. Penninx, B. W., Ferrucci, L., Leveille, S. G., Rantanen, T., Pahor, M., & Guralnik JM. Lower extremity performance innondisabled older persons as a predictor of subsequent hospitalization. Journals Gerontol Ser A Biol Sci Med Sci. 2000;55(11):691–7.
  5. Volpato, S., Cavalieri, M., Guerra, G., Sioulis, F., Ranzini, M., Maraldi C, Guralnik JM. Performance-based functional assessment in older hospitalized patients: Feasibility and clinical correlates. Journals Gerontol Ser A Biol Sci Med Sci. 2008;63(12):1393–8.
  6. Ellingsen Ø, Halle M, Conraads V, Støylen A, Dalen H, Delagardelle C, et al. High-Intensity Interval Training in Patients with Heart Failure with Reduced Ejection Fraction. Circulation. 2017;135(9):839–49.

10

Correspondent Campus Virga Jesse Stadsomvaart 11

3500 Hasselt

Ethical Review Committee

Advice Form

(X) Study Protocol

( ) Amendement protocol ( ) Medican need program

ONS KENMERK

Hasselt, 5 december 2019

## Title Protocol: Effects of High Intensity Interval Training versus Circuit Resistance Training on Endothelial Function and Cardiorespiratory Capacity in Patients with Heart Failure:A Randomized Trial

Belgish registration: B243201942026

Lead Researcher: Prof. Dr. Dominique Hansen

## DEFINITIVE

FINAL APPROVAL OF ETHICS REVIEW COMMITTEE JESSA

11

Dear Colleague,

On 21/11/2019, the Ethics Review Committee made comments regarding the submitted study file. We hereby confirm that we have received your modified study application:

- 19.94-REVA19.06_Reasons_adjustments
- Application form_01122019_first adjustments
- InformedConsent_December2019
- Instruction sheet

The amended documents comply with the stated comments and will comply with the study file being added. The Ethics Review Committee hereby gives its final approval for the start of the research. This approval applies to all participating sites, as stated in the preliminar advice. This approval is valid until 31/12/2020.

The Ethical Review Committee is organized and acts according to the guidelines of GCP / ICH.

After the advice on the initial / file has been issued by the Ethical Review Committee, you can there is no amendment to add a new research site for 3 months were submitted.

If there are changes to the study and / or approved documents, the investigator is required to report these changes to the Ethics Review Committee, as stipulated in the internationally established guidelines by the International Conference on Harmonization; ICH E6: Good Clinical Practice, Consolidated Guideline CPMP / ICH

/ 135/95:

The researcher refrains from deviating from the protocol or implementing changes without permission from the sponsor and review / approval by the appropriate ethical commission, except when it concerns an immediate threat to the participant or When it concerns administrative changes (such as change of telephone number, ...).

12

The investigator is required to immediately report to the Ethics Review Committee, in following cases:

- - Deviating or changing the protocol to pose an immediate danger to the participant appearance.
  - Changing the protocol which increases the risk for the participant or which increases the course of the study changes significantly.
  - Preventing Adverse Drug Reactions (Adverse Drug Reactions) that are serious and unexpected.
  - New information that can affect the safety of the participant.

Inform the Ethics Review Committee if a study is not started or when it is closed or interrupted prematurely (with reasons). If the inclusion has not started one year after approval, approval for the study expires and must be completed a new application was submitted to the Ethics Review Committee for approval. In addition, the Ethics Review Committee asks the researcher to inform faar / ifks were kept from the course of the study. If this is done with, the approval will expire the stud1e. In addition, the Ethics Review Committee asks that upon termination, the following be taken:

- - the end of the study (end date, number of treated / patients, possible complications and my overall impression).
  - any publicatles.

Please find attached the list of members of the Ethical Review Committee. With best regards,

For approval,

Vo te Assessment Committee Jes is

December 5, 2019

13

Members of the Ethics Review Committee 2019

Dr. Koen Magerman, clinical bio / eye (chair) Mieke Bieghs, pharmacist / substitute Inge Dreesen Joyce Bollen - nurse and lie. moraa / sciences

Dr. Martin Herklots, neurologist Je5 ~

HOPITAL

Rosita Jakers, psycho / age / alternates Hanne Heymans / Kelly Pauwels Dr. Herman Kuppers - general practitioner

Anne / a Lintermans - patient representative Fabienne Mertens - head nurse

Dr. Jean-Luc Rummens, clinical bio / eye Dr. Geert Souverijns, radiologist

Dr. Bjorn Stesse / - Anesthetist

Kimberly Vanhees - operational manager biobank Ohr. Pros Vanhe / mont - lawyer

Dr. Johan Vanwal! Eghem, nephro / eye (vice-chair)

Dr. Pascal Vranckx - cardiologist, expert pharmacology, pharmacotherapy, pharmacokinetics and clinical trial

design methodology expert

Dr. Renate Zeevaert - pediatrician

14


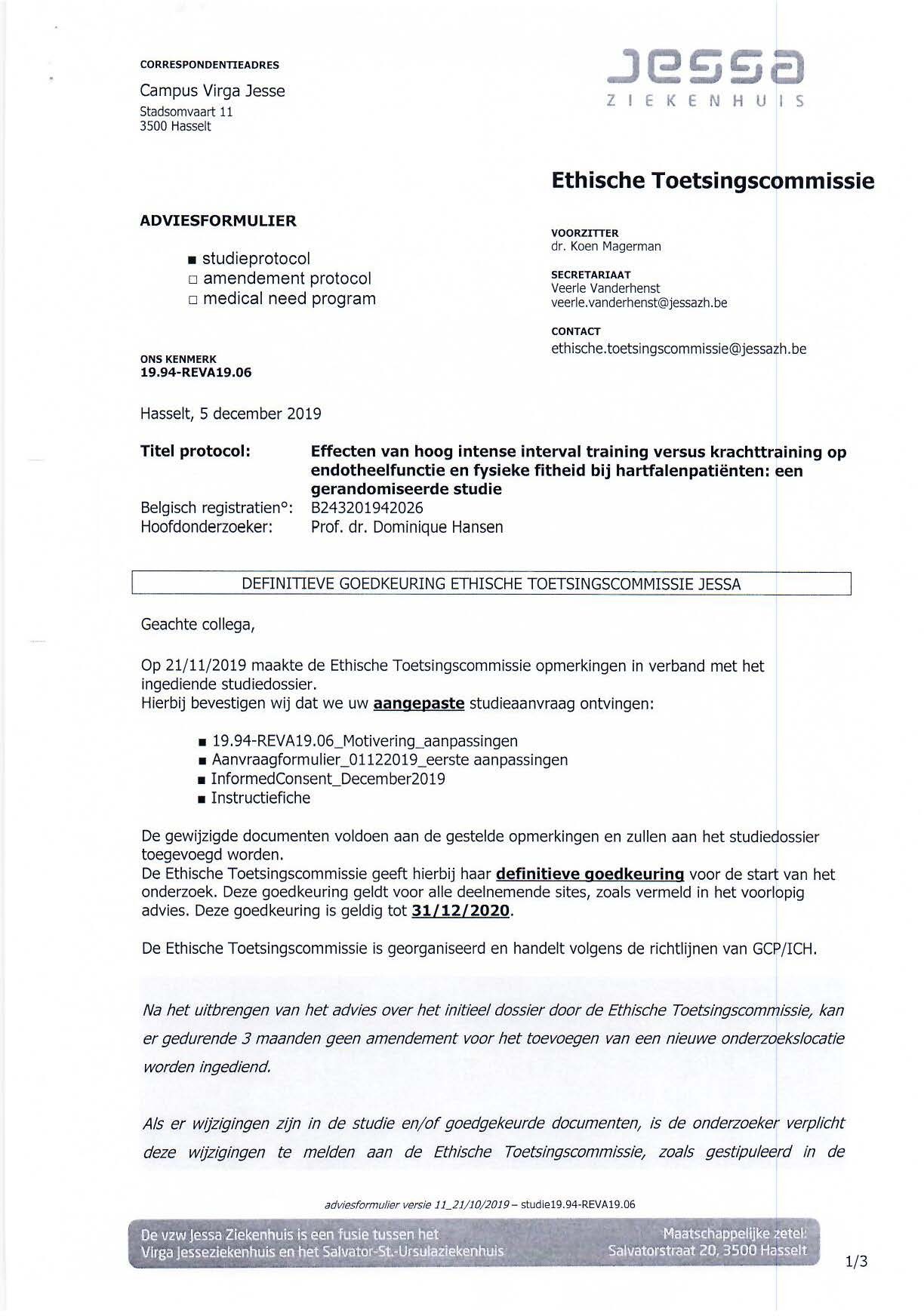


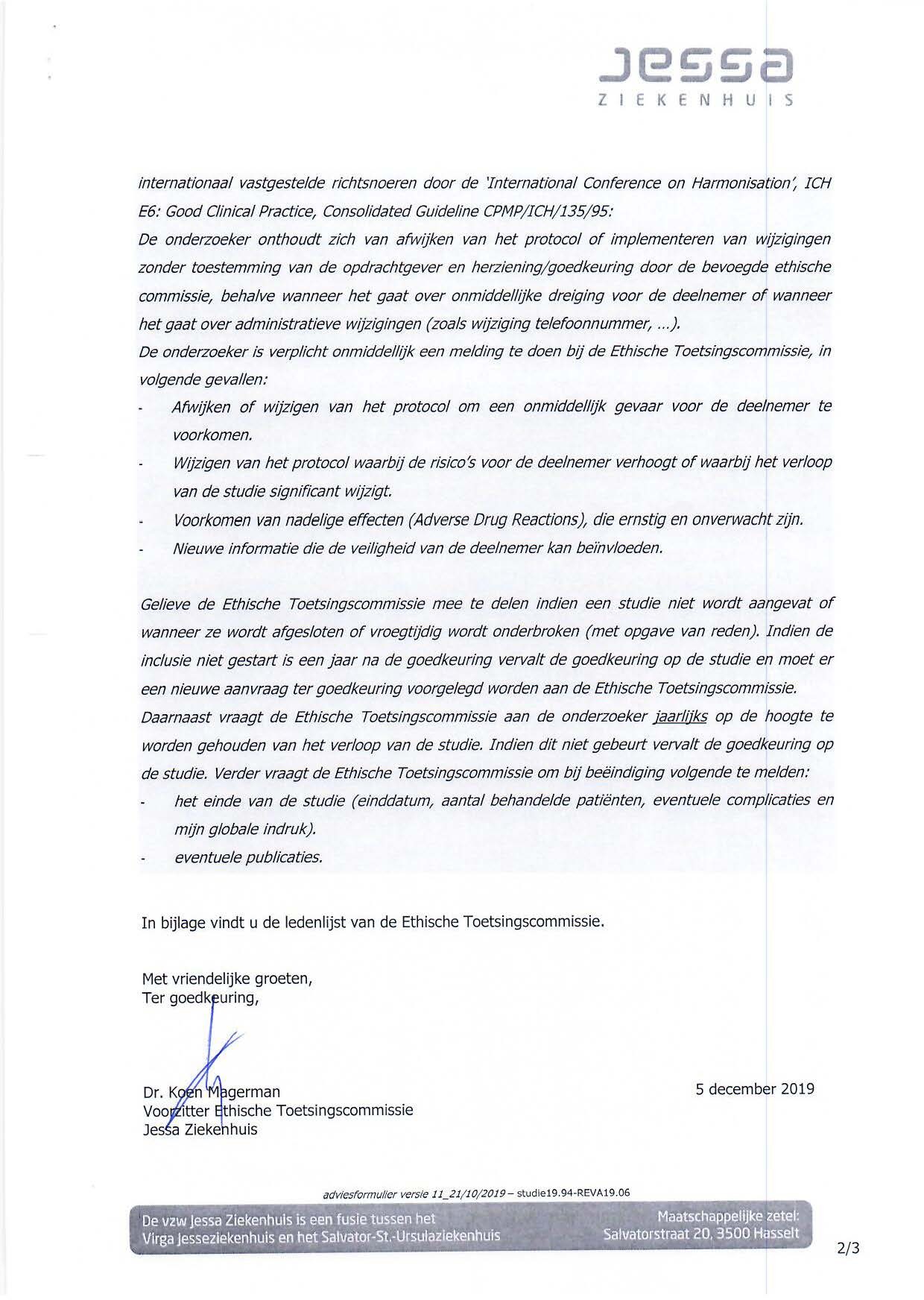


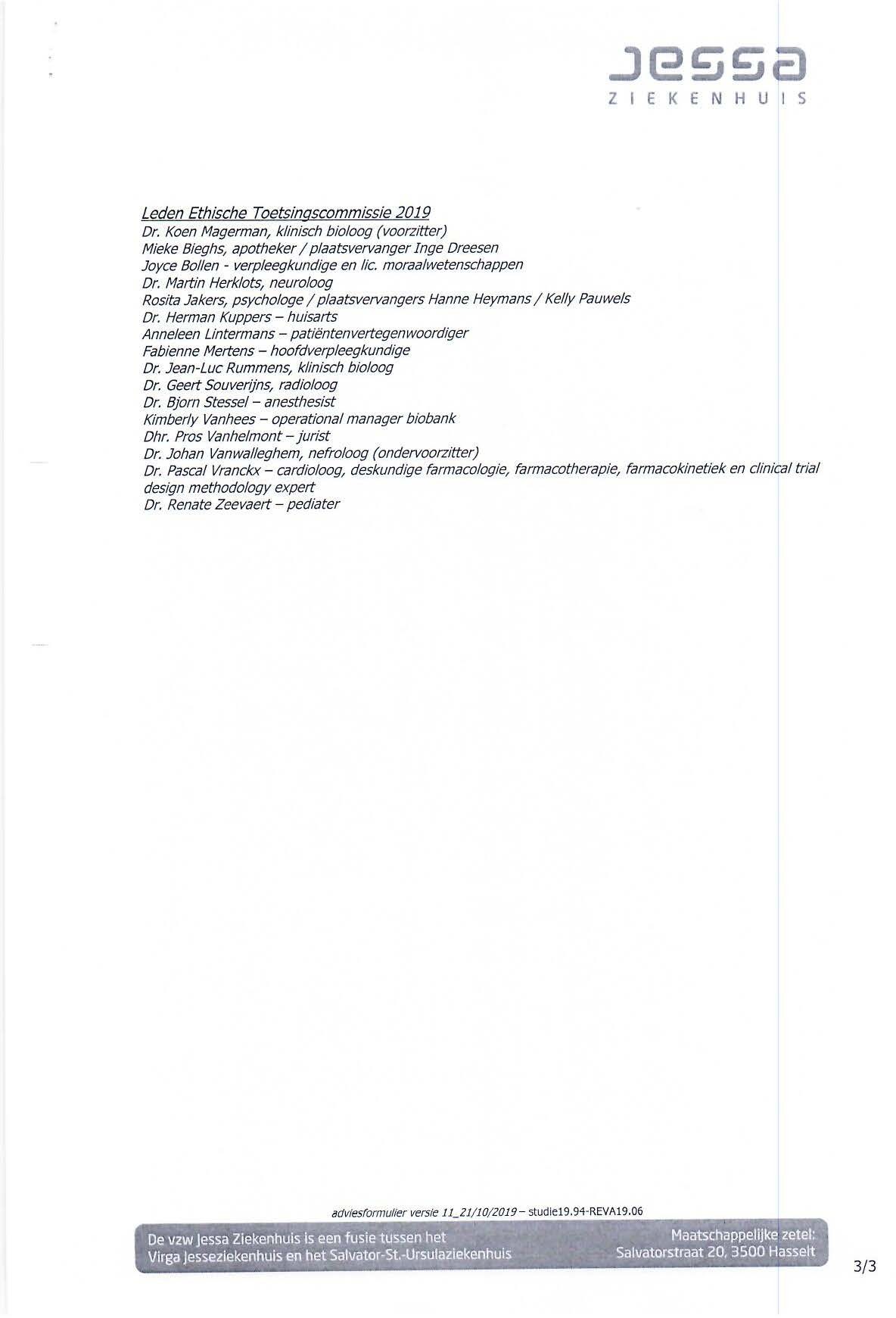


Projeto de Pesquisa de Doutorado

**COMPARAÇÃO DO TREINAMENTO AERÓBIO DE ALTA INTENSIDADEVS. TREINAMENTO RESISTIDO EM CIRCUITONA FUNÇÃO ENDOTELIAL E NA CINÉTICA DE OXIGÊNIO NA INSUFICIÊNCIA CARDÍACA: ENSAIO CLÍNICO RANDOMIZADO**

NATÁLIA TURRI DA SILVA

Colaboradores: Amanda Oliveira do Vale Lira, Lilian Bocchi Portugal, Marianne Lucena da Silva, Tatiana Zacarias Rondinel, Alexandra Corrêa de Lima, Júlia Fontenele dos Santos, Amanda Rafaely Baldoino, Daniela Mendonça,Jessica Desirre Lima Macedo de Oliveira, Luciana D’Ávila, Sérgio Henrique Rodolfo Ramalho,Robson Fernando Borges, Dominique Hansen, Graziella França Cipriano

Universidade de Brasília – UnB, Faculdade de Ceilândia – FCE. Projeto apresentado ao Programa de Pós- Graduação em Ciências e Tecnologias em Saúde.Área de Concentração: Promoção, Prevenção e Intervenção em Saúde.Linha de Investigação: Saúde, Funcionalidade, Ocupação e Cuidado.Temática: Fisiologia Clínica do Exercício.

**Orientador:** Prof. Dr. Gerson Cipriano Júnior

Brasília Dezembro de 2017

**RESUMO**

**INTRODUÇÃO**: Indivíduos com insuficiência cardíaca (IC)apresentam dispneia e fadiga, sintomas que diminuem a tolerância ao exercício e o desempenho físico funcional. Dentre as modalidades de treinamentos físicos que podem contribuir com este quadro,o treinamento aeróbio intervalado de alta intensidade (TAAI) e o treinamento resistido em circuito (TRC) são alternativas que já mostraram benefícios. Porém ainda há lacunas importantes quanto aos seus efeitos na função endotelial (FE) e cinética de oxigênio (∆VO2) e preencher esta lacuna pode trazer informações relevantes quantotratamento destes indivíduos. A potencial melhora na FE e ∆VO2 pode resultar em um melhor aporte sanguíneo e de oxigênio para musculatura periférica. **OBJETIVO**:Analisar e comparar as modalidades TAAI e TRC nos desfechos função endotelial e cinética de oxigênio em pacientes com IC.**MÉTODOS**: Ensaio clínico randomizado controlado, longitudinal, paralelo envolvendo pacientes IC com fração de ejeção reduzida com idade maior ou igual a 35 anos, divididos em 3 grupos: treinamento aeróbio intervalado de alta intensidade, treinamento resistido em circuito e grupo controle. Os treinamentos ocorrerão 3 vezes por semana, totalizando 36 sessões. As avaliações de função endotelial e cinética de oxigênio ocorrerão nos momentos pré e pós intervenção. A função endotelial será avaliada pela dilatação mediada pelo fluxo (DMF)e a avaliação da cinética de oxigênio será realizada em bicicleta ergométrica seguindo protocolo de exercício em carga constante.

1. **INTRODUÇÃO**

As doenças cardiovasculares tem sido a principal causa de morte no Brasil desde o final da década de 1960, dentre as quais a insuficiência cardíaca se destaca por sua maior prevalência (IC)(1). A IC gera dispneia e fadiga, sintomas que diminuem a tolerância ao exercício, repercutindo no desempenho físico funcional destes pacientes(1)^.^O treinamento físico por meio de exercícios é essencial para melhora deste quadro, e são fortemente recomendados (indicação de Classe I) para pacientes após um evento cardiovascular (2). A literatura aponta benefícios obtidos tanto por meio da modalidade aeróbia ou resistida, das quais o treinamento aeróbio intervalado de alta intensidade e o treinamento resistido em circuito (TRC)fazem parte.Entretanto, não se sabe quais destas modalidades geram melhores respostas sistêmicas na IC.

Em relação ao treinamento aeróbio intervalado de alta intensidade (TAAI), recente metanálise (Xie 2017) indicou melhores respostas na capacidade cardiorrespiratória quandocomparadas atreinamentos com intensidades menores em pacientes com IC(3).Nesta metanálise foi observado maiores valores deVO2 pico, o que ocorreuindependentemente da idade ou do limiar anaeróbio(3). Tal achado corrobora

com a informação de que a intensidade parece ser um importante preditor da eficácia dos programas de reabilitação cardíaca, mesmo após ajuste para outras variáveis relacionadas ao treinamento(4). Mais recentemente, estudo multicêntrico demonstrou não haver vantagens do TAAI em pacientes com IC em comparação ca moderada intensidade. Entretanto,neste estudo51% do TAAI treinaram abaixo da intensidade adequada enquanto 80% do treinamento aeróbio moderado treinaram acima da intensidade pretendida(5). Desta forma, o TAAI nessa população parecem ainda ser destaque terapêutico(3,4,6).

Quanto a modalidade treinamento resistido, apesar de quando isolado ser benéfico para ganho de força muscular, sabe-se que esta modalidade exerce menorinfluência do que o treino aeróbio para ganho de capacidade cardiorrespiratória(7), reforçando assim a vantagem da combinação destas duas modalidades de treino. Neste sentido o TRC se destaca, poisé capaz de estimular também as adaptações dos sistemas aeróbio e cardiovascular uma vez que demanda maiores valores de frequência cardíaca durante o treino(8). Isto porque os valores máximos de FC ocorrem, normalmente, durante as últimas repetições de uma série(8). O TRC também já mostrou benefícios na IC na força muscular esquelética, VO2 pico(9–11) além de já ter sido expresso forte correlação entre a mudança na taxa de produção de ATP mitocondrial do músculo esquelético e a mudança no consumo máximo de oxigênio do corpo total (VO2 pico) (11). Tais achados demonstram que o TRC parece ser uma modalidade interessante para pacientes com IC.

Apesar dos benefícios já relatados tanto do TAAI quanto do TRC, ainda há lacunas importantes sobre os efeitos de ambos os treinamentos sobre a população IC. Entender a totalidade dos efeitos das modalidades de treinamentos físicos na saúde destes pacientes é importante pois contribui com a adequada escolha terapêutica na reabilitação dos mesmos. Neste sentido não foram encontrados estudos avaliando as respostas na função endotelial e tampouco quanto a cinética de oxigênio entre estas modalidades nestes indivíduos. A importância de se estudar tais desfechos será elucidada nos parágrafos subsequentes.

O estudo da função endotelial em pacientes com IC é fundamental, visto que estase encontra reduzida. A redução da função endotelial é resultado de uma menor produção de oxido nítrico(12)e aumento do estresse oxidativo(13–15), que gera aumento da resposta de vasoconstrição e resistência vascular. Isto compromete a vasodilatação periférica avaliada por meio da dilatação mediada pelo fluxo (DMF), o que acarreta em redução do aporte sanguíneo para o músculo. Recursos terapêuticos como o exercício, são capazes de melhorar a função endotelial, podendo resultar em um melhor aporte sanguíneo para musculatura periférica e cardíaca (15)(16). A melhora da função endotelial reduz a disfunção cardíaca na IC(12)o que reforça a relevância de sua avaliação nas intervenções TAAI e TRC.

Além da importância evidenciada em se estudar a função endotelial em pacientes com IC, estudar a cinética do consumo de oxigênio (ΔVO2), ou seja, amagnitude e natureza do ajuste no consumo de oxigênio durante o exercício(17,18), é também relevante para estes pacientes. Isto porque a perfusão e

difusão de oxigênio (O2) encontra-se prejudicada nesta população graças a distúrbios dentro da via de transporte de O2, o que reduz a capacidade física nestes indivíduos(19). A melhora da cinética do consumo de oxigênio minimiza o prejuízo ocasionado pela IC nestes indivíduos, enfatizando a pertinência de sua avaliação após intervenções de treinamentos físico(19). Esta avaliação pode fornecer e elucidar as adaptações dentro do sistema de utilização e distribuição de O2 para o músculo esquelético fornecendo informações relevantes no estudo desta população (19), além defornecer recursos complementares para interpretação do teste ergoespirométrico.

A resolução dos mecanismos subjacentes à disfunção do músculo esquelético e à intolerância ao exercício é essencial para o desenvolvimento e aperfeiçoamento dos tratamentos mais eficazes para pacientes com IC.

Levando-se em consideração o exposto, e buscando sanar as lacunas na literatura a respeito do TAAI e TRC, o presente estudo visará analisar e comparar tais modalidades nos desfechos função endotelial e cinética de oxigênio em pacientes com insuficiência cardíaca.

Hipotetiza-se que os indivíduos participantes do grupo TAAI promoverá melhores resultados comparado ao TRC, gerando maiores aumentos na função endotelial e otimização da curva de cinética de oxigênio.

1. **OBJETIVO GERAL**

Analisar e comparar os treinamentos aeróbio de alta intensidade e treinamento resistido em circuito em pacientes com insuficiência cardíaca

- 1. OBJETIVOS ESPECÍFICOS Objetivo Primário:

Analisar e comparar função endotelial, cinética de oxigênio e capacidade funcional antes e após intervenções de treinamento físico (TAAI e TRC) e grupo controle.

Objetivo Secundário:

Analisar e comparar respostas em modulação autonômica, composição corporal, qualidade muscular, qualidade de vida, força de preensão palmar, força muscular e capacidade físico funcional em pacientes com IC antes e após intervenções de treinamento físico (TAAI e TRC) e grupo controle.

1. **MATERIAL E MÉTODOS**
   1. DELINEAMENTO DO ESTUDO

Trata-se de um ensaio clínico randomizado controlado, multicêntrico, longitudinal, paralelo, de abordagem quantitativa. Estudo seguirá as recomendações preconizadas pelo CONSORT ^16^ para ensaios clínicos de alta qualidade metodológica, com participação de indivíduos portadores de IC alocados em três grupos distintos: protocolos de treinamento aeróbio intervalado de alta intensidade (TAAI), protocolo treinamento resistido em circuito (TRC) e grupo controle sem intervenção (GC).

- 1. AMOSTRA

A amostra será composta por indivíduos com diagnóstico de IC com fração de ejeção reduzida e preservada, hemodinamicamente estáveis, encaminhados por médicos cardiologistas de Brasília e região (Distrito Federal, Brasil), bem como da cidade de Hasselt (Bélgica).

Conforme dados provenientes de estudo piloto realizado no Brasil, é necessária uma amostra total de 42 indivíduos, considerando tamanho de efeito de 0,36 alfa 0,05 power 0,8 considerando a variável de desfecho FMD. Para cumprir o tamanho amostral proposto, a amostra total será composta por 18 participantes no Brasil e 24 na Bélgica.

- 1. CRITÉRIOS DE INCLUSÃO E EXCLUSÃO

Serão adotados como critérios de inclusão: indivíduos com diagnóstico de IC com fração de ejeção reduzida e preservada, de ambos os sexos (mulheres se na menopausa), idade maior ou igual a 35 anos, os quais deverão não ter realizado nenhum tipo de atividade física nos últimos 6 meses, indivíduos não fumantes, ausência de doenças respiratórias bem como de processo inflamatório ou infeccioso e lesão músculo tendínea ou osteoarticular, que desempenhasse limitação ao exercício. As medicações regulares aceitas serão apenas para controle de fatores cardiovasculares necessários de acordo com indicação de cardiologista. Os critérios de exclusão serão: pacientes que realizarem qualquer tipo de atividade física no período de reabilitação fora as que lhes serão atribuídas, indivíduos que não cumprirem a periodização faltando mais que 25% das sessões.

- 1. LOCAL DA PESQUISA

A pesquisa será realizada na Universidade de Brasília, Campus de Ceilândia e também na Universidade de Hasselt, Campus Diepenbeek, sendo que o treinamento físico ocorrerá no Ginásio Terapêutico da universidade e no Reval (Bégica, Uhasselt). As avalições para os desfechos apontados anteriormente serão realizadas no Laboratório de Fisiologia e Biofísica da Universidade de Brasília, Campus de Ceilândia e também no Jesse Hospital, Hasselt, Bélgica.

- 1. CONSIDERAÇÕES ÉTICAS

Todos os procedimentos utilizados nesse estudo serão encaminhados para aprovação pelo Comitê de Ética em Pesquisa da Instituição. Os participantes do estudo assinarão um termo de consentimento

livre e esclarecido confirmando a participação e comprovando ciência a todas as etapas do estudo, sendo que o mesmo, caso opte, poderá desistir ao longo do trabalho.

- 1. PROCEDIMENTOS E PROTOCOLO DE AVALIAÇÃO

Um pesquisador independente preparará a alocação de sequência aleatória randomizada, a qual será obtida por meio do software random.com. A alocação do tipo de tratamento será realizada na primeira visita ao fisioterapeuta, com pelo menos duas semanas de antecedência ao início das intervenções. O fisioterapeuta responsável saberá a intervenção adotada pelos voluntários, entretanto o avaliador de cada desfecho será cego.

As avaliações serão feitas em dois momentos para ambos os grupos: antes de iniciar o protocolo experimental e após o término do mesmo. Os desfechos a serem analisados nestes momentos serão: função endotelial, cinética do consumo de oxigênio, capacidade funcional, qualidade de vida, modulação autonômica, composição corporal,capacidade fisico funcional, qualidade muscular, força de preensão palmar.

- - 1. Vasodilatação mediada pelo fluxo (DMF)

Previamente, os sujeitos serão instruídos para evitar quaisquer sessões de exercícios planejadas e serão convidados a abster-se de consumir cafeína / álcool e praticar exercícios físicos durante 24 h antes dos testes. Os sujeitos serão orientados a participar da avaliação desde que no mínimo 2h pós- prandiais. Para evitar potenciais variações diurnas, os testes serão realizados sempre na mesma hora do dia e na mesma sala com temperatura controlada (~ 24 ° C).

Inicialmente, os indivíduos descansarão em posição supina durante um período de 15 min para garantir a obtenção e estabilização de variáveis cardiovasculares. O diâmetro da artéria braquial e a velocidade do sangue serão medidos usando ultra-sonografia duplex-Doppler de alta resolução (Sistema de Ultrassom HD11.XZ, 1 e 3 MHZ, Phillips, Barueri, SP, Brasil) seguindo as diretrizes presentes (20). Um transdutor de matriz linear de 9 MHz será colocado sobre a artéria braquial discretamente proximal à fossa cubital. Os sinais de diâmetro e velocidade serão obtidos simultaneamente em modo duplex em uma freqüência pulsada de 5 MHz e corrigidos com um ângulo de insonação de 60 °. O volume da amostra será ajustado para abranger todo o lúmen do vaso sem se estender além das paredes e o cursor será ajustado no meio do vaso. A DMF da artéria braquial será avaliada no braço direito na posição supina como descrito anteriormente (21,22).

Resumidamente, um manguito será acoplado no braço. Durante dois minutos de repouso os dados hemodinâmicos serão registrados, e então o manguito será insuflado até uma pressão de 220 mmHg e mantido assim durante 5 min. As medidas do diâmetro contínuo e da velocidade do sangue serão registradas continuamente durante 3 min após a deflação rápida do manguito. As análises de todas

as variáveis vasculares serão analisadas off-line usando o software de detecção de bordas especializado (Cardiovascular Suite, Quipu, Pisa, Itália). A variação da porcentagem de DMF foi normalizada para a área incremental de taxa de cisalhamento sob a curva até o diâmetro do pico (23)(24)

- - 1. Teste Cardiopulmonar de Exercício Incremental

A avaliação da capacidade funcional será realizada por meio de exame ergoespirométrico efetuado por médico especialista (cegado quanto à alocação). Este exame será importante para determinação dos limiares aeróbio e anaeróbio, necessários à prescrição doTAAI, além de também fornecer medidas de desfecho para os grupos, dentre as quais serão utilizadas VO2 pico e VE/CO2, reportados em recente revisão sistemática em protocolos de treinamento como os parâmetros mais amplamente usados e indicativo de resultados (25).

Os pacientes se apresentarão ao Laboratório de Fisiologia e Biofísica da Unb, para realização do teste incremental limitado por sintomas, em bicicleta eletromagnética com sistema 0-watt (Corival, Lode Co., Groningen, Holanda) utilizando um protocolo de rampa (5- 10 watts/minuto). Antes do início de cada teste, será observado um período de 5 minutos para adaptação ao cicloergômetro e a estabilização das trocas gasosas. O eletrocardiograma de 12 derivações será monitorado continuamente (T12, Cosmed, Roma, Itália) com registro associado ao programa de captação 27 de gases. A pressão arterial será verificada com esfigmomanômetro padrão, com o paciente sentado no próprio ergômetro, a cada 2 minutos durante o exame e até 15 minutos após o final da parte ativa do teste. Os gases expirados serão coletados por alíquotas a cada respiração por um analisador de gases computadorizado (Quark CPET, Cosmed, Roma, Itália).

- - 1. Avaliação da cinética de oxigênio

A avaliação da cinética de oxigênio será realizada em bicicleta ergométrica estacionária. Para isso, os voluntários serão orientados a manterem-se sentados, com máscara de captação dos gases expirados por um analisador de gases computadorizado (Quark CPET, Cosmed, Roma, Itália). Os voluntários serão orientados a utilizarem vestimentas adequadas para este teste de exercício previamente a data de avaliação. Não será permitida a circulação de pessoas pela sala durante a execução do teste, de modo a reduzir a ansiedade dos indivíduos e os erros de captação.

A avaliação da cinética de oxigênio será realizada de acordo com protocolo de exercício em carga constante na bicicleta ergométrica estacionária, realizado com uma fase inicial sem carga (0 watt start- up system, Lode, Holanda) por três minutos e logo após um exercício inicial com carga constante, realizado abaixo do LA (limiar anaeróbio), durante 6 minutos com uma carga constante sub-LA (1ª sessão). Logo após a sessão de exercício moderado será realizado um intervalo de 15 minutos e um novo exercício na sequência, realizado com uma fase inicial de carga 0 watt por três minutos e após isso um exercício com carga supra-LA (80%Δ VO2max) realizado até o máximo possível (Tlim) e após o exercício será realizado a coleta dos gases expirados por 15 minutos.

Durante o teste serão analisados o consumo de oxigênio ( O2), produção de dióxido de carbono ( CO2), ventilação minuto ( E), volume corrente (VC), frequência respiratória (ƒ), razão de troca respiratória (R), equivalentes ventilatórios para oxigênio ( E/ O2) e dióxido de carbono ( E/ CO2), tempo inspiratório (TI), tempo expiratório (TE), e relação TI/TTOT. Os dados continuarão a ser coletados mesmo após o término do exercício, pelo menos 15 minutos de recuperação passiva.

Durante todo o protocolo será registrado o índice de percepção de esforço para fadiga dos membros inferiores. O eletrocardiograma de 12 derivações será continuamente monitorizado. Os indivíduos serão questionados acerca da sensação de esforço ventilatório e cansaço nos membros inferiores a cada 2 minutos, de acordo com a escala Borg. O sistema será calibrado diariamente antes de cada teste.

- - 1. Avaliação da Força Muscular

A força Muscular será avaliada por meio do teste de 1RM, o qual irá determinar a carga máxima, que cada indivíduo conseguirá realizar durante o movimento exigido pelo exercício em questão, para posteriormente, serem determinadas as cargas de treinamento. Este teste representa a maior resistência que pode ser movimentada através da amplitude de movimento plena de uma maneira controlada e com boa postura.

O teste se inicia recebendo incrementos de acordo com a percepção do sujeito, até ser concluído quando o voluntário alcançar a carga máxima, na qual conseguir executar o movimento sem falha mecânica. Não será permitido mais do que cinco tentativas para estabelecimento desta carga máxima e caso isto ocorra, o teste será desconsiderado com agendamento de nova data para avaliação. Esta variável será coletada no momento basal antes do início dos treinamentos e 72 horas após o término da última sessão.

- - 1. Avaliação da modulação autonômica

A avaliação da modulação autonômica será realizada por meio da Variabilidade da Frequência Cardíaca (VFC). Para isso, os voluntários serão orientados a manterem-se em repouso, acordados, na posição supina e respiração espontânea por 30 minutos por meio de cardiofrequencímetro da marca Polar® RS800 (Polar Electro OY, Finlândia) no pulso**,** equipamento previamente validado quanto a sua utilização para captação dos intervalos entre batimentos cardíacos consecutivos (em ms)(26). Os voluntarios serão orientados ao não consumo de estimulantes do SNA 24h antes desta avaliação. Não será permitida a circulação de pessoas pela sala durante a execução das coletas, de modo a reduzir a ansiedade dos indivíduos e os erros de captação. Os dados obtidos de VFC serão transferidos para um computador por meio do software Polar Pro Trainer e, posteriormente, para cálculo dos índices de VFC será utilizado o software Kubios HRV - versão 2.0 (Kubios, Biosignal Analysis and Medical Image Group, Department of Physics, University of Kuopio, Finland), considerando 1000 intervalos sinusais (mínimo de 95% de batimentos sinusais). Isto ocorrrá após filtragem digital (software Polar Pro Trainer)

complementada por manual (Microsoft Excel), para eliminação de batimentos ectópicos prematuros e artefatos.

Para análise da VFC serão usados índices lineares, obtidos nos domínios do tempo (RMSSD e SDNN) (27), índices geométricos (*plot* de Poincaré, interpolação triangular dos intervalos NN (TINN)(27) e índice triangular (RRtri) (28) e no domínio da frequência avaliados por meio da transformada rápida de Fourier segmentada em baixa frequência (LF – Frequência entre 0,04 a 0,15Hz), alta frequência (HF – Frequência entre 0,15 a 0,4Hz) e relação LF/HF expressos em unidades normalizadas e em ms(27). Métodos não lineares já validados também serão utilizados como Recorrência (REC), Análise depurada de frequência (DFA alfa 1 e alfa 2), Determinismo (DET) e entropias (Apen e Sampen).

- - 1. Avaliação da composição corporal

A composição corporal será estimada por meio da DXA (DPX-MD, software 4.7; marca Lunar, Madizon, WI, EUA). Os sujeitos serão posicionados numa posição supina durante todo o exame, onde deverão ficar imóveis durante um tempo de cerca de 10 minutos. Massa de gordura (MG) e massa livre de gordura (MLG) serão expressas em valores absolutos. Para classificar os grupos em relação à composição corporal, por causa da ausência de pontos de corte, utilizar-se-á a mediana da quantidade maior ou menor de MG e MLG.

- - 1. Avaliação da espessura e ecogenicidade muscular

Os indivíduos serão avaliados em posição supinacom o joelho em extensão passiva e rotação neutra. Uma solução aquosa, denominada gel de transmissão será aplicado à cabeça de ultra-som para permitircontato acústico sem pressionar a superfície dérmica. Duas imagens serão adquiridas na perna direita: (1) imagem anterior:transdutor colocado perpendicular ao eixo longo da coxa anterior,dois terços da distância da espinha ilíaca anterior superior ao superiorborda da patela (29), e (2) imagem lateral: 5 cm lateralmente a partir do primeiroponto de imagem. A imagem lateral será obtida em um campo prolongadode modo de visão em uma distância de 10 cm correndo em uma direçãocrânio-caudal. Para habilitar a replicação da localização da imagem no ultra-som pós treinamento físico, uma marca será desenhada sobre a perna do voluntário e uma foto será registrada para conferência no momento final. As imagens serão salvas no disco rígido do ultra-som e transferidas para análise posteriorem um computador usando o software ImageJ (NIH, Bethesda, MD) (30)**.**

Todas as medidas de ultra-sonografia serão realizadas três vezes, com a média dos escores utilizados nas análises finais. Na imagem anterior, espessura muscular e ecogenicidade de vasto intermédio, reto femoral, espessura do tecido subcutâneo e área transversal de reto femoralserão avaliadas. Todo os parâmetros de espessuraserão medidos em centímetros, e aárea transversal de reto femoralserá medida em centímetros quadrados e no ponto mais largo do músculo.

A ecogenicidade será relatada em pixels. A ecogenicidade será determinada usando análises quantitativas por escalas quantificadas por computador. Um padrão quadrado 2 × 2 cm para análise dos músculos reto femoral e vasto intermédio separadamente será usado para determinar a região de interesse (ROI).O método quadrado tem uma confiança mais forte em comparação com o método de rastreamento (onde o avaliador destaca toda a área muscular visível excluindo epimísio e artefatos) para definir o ROI (31). Se a área a analisar for menor do que 2 × 2 cm, o maior quadrado possível dentro dos limites anatômicos do músculo será examinado. Média e desvio padrão da ecogenicidade deste ROI serão calculados usando a função de histograma do software ImageJ (NIH, Bethesda, MD) e expresso como um valor entre 0 (= preto) e 255 (= branco) (32). Na imagem lateral, as medidas incluirão espessura vasto lateral e ângulo de penação do vasto lateral (ângulo em graus entre fascículos de fibras musculares e Aponeurose muscular profunda).

As medidas de ultra-sonografia serão realizadas em dois momentos: antes de iniciar o protocolo de treinamento e 72 horas após o termino da última sessão do mesmo.

- - 1. Classificação dos estados de funcionalidade e incapacidade (CIF)

A classificação dos estados de funcionalidade e incapacidade será avaliada por meio da CIF em sua versão abrangente, que consiste na aplicação de todos os seus códigos. A avaliação da CIF será em forma de entrevista feita por fisioterapeuta previamente treinado para uso do instrumento, conforme Martins et al. 2010(33).

Os códigos da CIF serão estabelecidos a partir dos seguintes componentes: I – Funções do corpo; II Estruturas do corpo; III – Atividade e Participação; IV – Fatores Ambientais. Os componentes I, II, e III estão relacionados com parte 1 da CIF, destinados a classificar funcionalidade e incapacidade e o componente IV estão relacionados com a classificação dos fatores contextuais.

Será realizado pelo pesquisador o registro manual do número de ocorrências para todos os níveis de codificação, de todos os sujeitos. Contudo, no processamento dos dados, serão consideradas apenas as ocorrências de primeiro nível (capítulos) para classificação. Cada código especificado será relacionado com seu respectivo qualificador, sendo o qualificador 0 determinante de funcionalidade, ou seja, nenhuma deficiência para aquele código. Os qualificadores de 1 a 4 serão considerados como determinantes de incapacidade, pois classificam a presença de deficiência de ligeira a completa. O qualificador 8 será considerado como não especificado, quando não se podia determinar a presença ou não de deficiência, e o qualificador 9, como não aplicável, quando o código não era aplicável.

- - 1. Força de preensão palmar

Para mensuração da força de preensão palmar será utilizado o instrumento handgrip.

O posicionamento dos participantes será de acordo com a *American Society of HandTherapists Guidelines*(34): sujeito sentado com braço apoiado, ombros aduzidos, em rotação neutra, cotovelo

fletido a 90 °, antebraço na posição neutra e punho entre 0 e 30 ° de dorsiflexão. Três medidas com a mão dominante e não dominante serão realizadas. O valor mais alto será expresso em kg, e incluído nas análises. As manobras devem ser realizadas com 5 segundos de sustentação, e 3 minutos de descanso entre uma e outra. Instruiremos os pacientes a manterem a respiração espontânea e a evitarem a realização da manobra de Valsalva, conjuntamente ao exercício.

Os participantes serão instruídos a manter o posicionamento durante os testes e corrigidos pelo examinador quando necessário. Acessórios tais como relógios, pulseiras, anéis e braceletes serão removidos de ambos os membros superiores dos participantes antes do início dos testes. Todos os participantes serão avaliados individualmente. Os participantes serão orientados a não olhar para o mostrador do dinamômetro para evitar qualquer retorno (*feedback*) visual.

- - 1. Bateria curta de performance física (SPPB)

A SPPB será lida, e aplicada, para os sujeitos visando reduzir o risco de viés. Os domínios explorados pelos questionários serão pontuados como previsto pela SPPB.

Considerando que a*Short Physical Performance Battery* (SPPB) é um instrumento composto por três testes distintos, as avaliações funcionarão da seguinte forma: 1)equilíbrio estático - avaliado em três posições - *side by side* (pés unidos), *semi tandem*(um pé parcialmente a frente do outro) e *tandem* (um pé a frente do outro); 2) velocidade de marcha (cronometra-se o tempo gasto para percorrer três metros em passo habitual); 3) força dos membros inferiores (cronometra-se o tempo gasto para levantar e sentar de uma cadeira por cinco vezes consecutivas, sem auxílio das mãos). A pontuação varia de 0 a 4, para cada um dos testes e de acordo com o tempo realizado em cada tarefa, sendo 0 o pior desempenho e 4 o melhor desempenho. Na impossibilidade de realização de qualquer uma das etapas a pontuação será zero. (35)

O escore total do teste é resultado da somatória dos três testes já citados, ou seja, 12 pontos, sendo

0 o pior desempenho e 12 o melhor desempenho. Escore de 0 a 3 pontos serão considerados incapacidade ou desempenho muito ruim, escore de 4 a 6 pontos serão considerados baixo desempenho; 7 a 9 pontos serão de moderado desempenho e escore de 10 a 12 pontos serão considerados bom desempenho. (35)

- - 1. Avaliação da força muscular isocinética e extração periférica de oxigênio

Para avaliação da força muscular isocinética, será utilizado o dinamômetro BIODEX System 3 PRO, *New York*.

Os sujeitos serão posicionados com o quadril a 90° com o eixo articular alinhado com o fulcro do dinamômetro de forma que o joelho do membro avaliado fique livre (distância de 2 dedos) e o sujeito esteja com todo o glúteo próximo do encosto da cadeira. Será verificado o alinhamento da perna avaliada com o membro contralateral. Todas as mediadas de posicionamento do sujeito no dinamômetro (altura da cadeira; base da cadeira; encosto da cadeira; distância do dinamômetro; distância do braço) será registrado em ficha confeccionada pelos pesquisadores a fim de tornar a medida reprodutível após a intervenção. Os sujeitos realização a extensão de joelho a 70°^s-1^, para avaliação do pico de torque isométrico, 5 vezes por 4s com tempo de repouso de 30s entre as repetições. Será considerado para análise a média dos 3 maiores valores. Para a medida de força dinâmica, os sujeitos realizarão a extensão de joelho a 180°^s-1^ por até 30 repetições.

A avaliação da extração periférica de oxigênio ocorrerá de forma contínua e não invasiva por meio do*NIRS* (*Near-Infrared Spectroscopy* Portamon (Artinis Medical Systems, Einsteinweg 17, 6662 PW, Elst, The Netherlands). Na região próxima ao infravermelho, a hemoglobina - incluindo suas duas principais variantes: a oxihemoglobina (O 2 Hb) e a desoxihemoglobina (HHb) - exibem absorção dependente do oxigênio. Usando um número de comprimentos de onda diferentes, as alterações relativas na concentração de hemoglobina podem ser exibidas continuamente. O posicionamento será no músculo vasto lateral da perna avaliada, visualizado local de maior ventre muscular após contração isométrica, onde a fixação do aparelho será por meio de tiras de velcro e coberto por uma bandagem preta para eliminar a luz ambiente. Esta mensuração iniciará 5 minutos antes dos procedimentos de avaliação de força muscular isocinética, será mantida durante toda a avaliação, e 5 minutos após o término. O posicionamento do *NIRS* será demarcado com caneta demográfica e registrado por meio de câmera fotográfica. As variáveis analisadas serão índice de oxigenação tecidual (TSI%), oxiemoblogina (O2Hb), deoxiemoglobina (HHb), hemoglobina total (tHb) e diferencial de hemoglobina (HbDiff). Os dados serão analisados com OxySoft Software (v2.1.2, Artinis Sistemas Médicos). O índice de saturação tecidual (TSI) será calculado usando a equação abaixo:

[𝑂2𝐻𝑏]

𝑇𝑆𝐼 = 100 𝑥 [𝑂2𝐻𝑏 + 𝐻𝐻𝑏]

- - 1. Função pulmonar e força muscular respiratória

A função pulmonar e a força muscular respiratória são importantes avaliações para o método de seleção/avaliação respiratória dos pacientes (anamnese). Tais avaliações serão acessadas pelos testes de

espirometria (MicroLab ML3500MK8, CareFusion, EUA), manovacuômetro (MVD300, Globalmed, Brasil) e pressão inspiratória dinâmica .

Os indivíduos serão instruídos a abster-se de estimulantes autonômicos (bebidas alcoólicas, café, chá ou alimentos contendo cafeína) durante 24 horas antes da avaliação, bem como a ingestão de uma refeição leve pelo menos 2 horas antes da medição. Os pacientes devem descansar 10 minutos antes de cada teste (espirometria, pressões respiratórias máximas e pressão inspiratória dinâmica). Eles serão colocados em um banco com as pernas dobradas a 90 graus, postura ereta, cabeça em posição neutra e clipe nasal para evitar vazamentos.

Espirometria

Os indivíduos realizarão pelo menos três manobras de expiração forçada de acordo com os procedimentos técnicos e critérios de aceitabilidade e reprodutibilidade da American Thoracic Society (ATS) / European Respiratory Society (ERS)(36). A avaliação espirométrica será realizada para verificar padrões obstrutivos ou restritivos através de medidas de desfecho: volume expiratório forçado no primeiro segundo (VEF1), capacidade vital forçada (CVF), pico de fluxo expiratório (PFE), relação VEF1 / CVF.

Manovacuômetro

O manovacuômetro MVD300® (Globalmed, Porto Alegre, RS, Brasil) foi utilizado para medir as pressões positivas (manômetro) e as pressões negativas (vacuômetro). O manovacuômetro permite a avaliação estática da pressão inspiratória máxima (PImáx) e da pressão expiratória máxima (PEmáx) e desempenha importante papel no diagnóstico e prognóstico de doenças crônicas (37).

O manovacuômetro será previamente calibrado em cmH2O, com limite operacional de -300 a

+300 cmH2O e escalas variando de 10 a 10 cmH2O. Os métodos e critérios utilizados serão os recomendados pela ATS / ERS (38). Para avaliar a PImáx, os pacientes serão instruídos a realizar uma expiração máxima, seguindo um esforço inspiratório máximo, para que a pressão seja registrada próxima ao volume residual. Para a avaliação da PEmáx, uma inspiração máxima será solicitada antes do esforço expiratório máximo para avaliar a pressão, próximo da capacidade pulmonar total. Um vigoroso comando verbal será dado durante a avaliação.

Pressão inspiratória dinâmica

A avaliação da força muscular dinâmica respiratória será realizada utilizando o dispositivo POWERbreathe® KH2 (Londres, Inglaterra, Reino Unido), no qual o esforço inspiratório máximo será avaliado a partir do volume residual. A força muscular máxima (índice S) será obtida durante a contração dinâmica dos músculos inspiratórios. Os pacientes serão instruídos a atingir a taxa de fluxo

inspiratório mais alta possível após uma expiração anterior. Para obter o índice-S, o paciente deve ser colaborativo e capaz de responder aos comandos verbais (38).

- - 1. NT-pro-BNP

O NT-pro-BNP será utilizado para caracterizar insuficiência cardíaca com fração de ejeção preservada ou reduzida (39), especificando critério de entrada dos pacientes juntamente com outras variáveis (anamnese) segundo *European Society Cardiology Guidelines*(40)*.*

Os pacientes não precisam jejuar ou ter qualquer preparação especial para o teste.

O soro de NT-proBNP será medido usando tiras de teste (CARDIAC proBNP +, Roche Diagnostics, Basiléia, Suíça) contendo anticorpos monoclonais e policlonais contra epitopos da molécula NT- proBNP em um dispositivo point-of-care (Cobas h232, Roche Diagnostics Basileia, Suíça). Uma amostra de sangue venoso será mantida em tubos heparinizados (sódio) (Vacuette da Greiner Bio-One, Roche Diagnostics, Basiléia, Suíça) à temperatura ambiente e analisados em até 5 horas. Os controles do fabricante serão utilizados para monitorar o controle de qualidade com limites de aceitabilidade definidos pelo fabricante (4). As avaliações serão sempre realizadas no laboratório de fisiologia por profissionais habilitados (cardiologistas colaboradores da pesquisa).

- - 1. Avaliação ecocardiográfica

Os indivíduos serão instruídos a abster-se de cafeína por 24 horas antes do teste.

O exame ecocardiográfico por strain com um transdutor de 4-2 MHz equipado com uma segunda imagem harmônica (HDI 5000 2-4 MHZ, Philips ATL, Bothell, WA) será usado para acessar a função cardíaca através do formato DICOM (Digital Imaging and Communication in Medicine) (31)(anamnese). As medidas de resultado incluem fração de ejeção do ventrículo esquerdo por Simpson (LEF,%), índice de volume do átrio esquerdo (VAE, ml / m²), índice de massa do ventrículo esquerdo (IMVE, G / M²), diâmetro diastólico do ventrículo esquerdo (DDVE, mm) e pressão arterial (PAP, mmH) seguindo recomendações da Sociedade Americana de Ecocardiografia e da Associação Europeia de Imagem Cardiovascular (41,42)

- 1. PROTOCOLO DE INTERVENÇÃO

O protocolo experimental deste estudo será submetido para a plataforma nacional de registro de ensaios clínicos <http://www.ensaiosclinicos.gov.br/>*.*

Importante salientar que será realizado estudo piloto como uma versão em miniatura do estudo principal para testar se os componentes deste estudo (todas as avaliações e etapas) podem funcionar concomitantemente. Este procedimento possuirá todos os desfechos do estudo principal que poderão

contribuir com a análise final bem como cálculo amostral e se necessário serão realizadas adaptações no delineamento do estudo para que o mesmo seja o mais adequado possível.

- - 1. Reabilitação cardíaca

O estudo será composto por três grupos randomizados de intervenção: TAAI – treinamento aeróbio de alta intensidade;TRC – treinamento resistido em circuito (TRC); GC – grupo controle.

Os grupos treinamentos realizarão exercícios três vezes por semana, com intervalo de pelo menos 24h entre as sessões até totalizar 36 sessões de treino.

Previamente a realização dos treinos será feita familiarização com os equipamentos da seguinte forma: para o TRC o indivíduo realizará 10 repetições com carga referida como um pouco intensa (13 na escala de Borg) orientado em boa postura; para o TAAI o indivíduo será orientado a caminhar na esteira ergométrica por 15 minutos com percepção um pouco intensa (13 na escala Borg). Estes critérios colaborarão para que os voluntários executem os movimentos com a melhor desenvoltura possível, sendo corrigidos por fisioterapeutas durante o período de familiarização.

Exercícios em solo e alongamentos globais serão usados com objetivo de aquecimento (15 minuto)

, a fim de evitar possíveis intercorrências, reduzindo risco de lesão. O intervalo entre as séries dos exercícios irá variar entre 40 segundos a 1 minuto e meio, respeitando a relação diretamente proporcional entre tempo e carga do exercício. Também ocorrerá familiarização com o equipamento previamente ao início das sessões.

Os grupos musculares eleitos para TRC serão: quadríceps, isquiotibiais, costas, peitoral, ombro, bíceps e tríceps. O teste de uma repetição máxima (1RM) será utilizado para mensuração da carga máxima de cada indivíduo, para posteriormente determinar as cargas de TR. As cargas do protocolo de TRC iniciarão à 30% de 1RM e aumentando gradativoao longo das sessões até 80% 1RM, (intensidade entre moderada e intensa) respeitando sempre os princípios de adaptação e sobrecarga. Já as cargas eleitas para execução de TAAI, que ocorrerá em esteira ergométrica e bicicleta ergométrica variarão entre duas intensidades, sendo a mais alta acima do primeiro limiar anaeróbio e a mais baixa, abaixo do primeiro limiar anaeróbio obtido pelo teste ergoespirométrico, sendo que as cargas serão alterada ao longo das sessões de acordo com a FC referente para atingir aos respectivos limiares.

Já em relação os indivíduos do GC, estes assistirão a palestras e serão orientados a realizarem atividade física leve de forma não supervisionada, 3vezes por semana, tanto aeróbia quanto resistida, sendo a intensidade de ambas considerada adequada se abaixo do primeiro limiar obtido no exame de VO2. Para isto o indivíduo será previamente instruído na sessão de avaliação ergoespirométrica quanto à percepção do esforço e o limite adequado para exercícios.

- 1. RISCOS

Os treinamentos físicos serão realizados no Ginásio Terapêutico da Universidade de Brasília, Câmpus Ceilandia, por fisioterapeutas treinados e durante a sua realização será acompanhado por uma médica cardiologista colaboradora (Dra. Alexandra CGB de Lima) que garantirá suporte médico ao longo de todo o trabalho a fim de controlar os riscos dos pacientes.

Durante a realização das sessões serão realizadas mensurações da pressão arterial (PA), saturação de oxigênio (SATO2), escala de percepção do esforço (Borg) pré e pós exercício e, caso necessário, durante a sua realização. Além disso, a frequência cardíaca (FC) dos indivíduos será avaliada pré e pós sessão além de ser continuamente monitorada por cardiofrequencímetro ao longo de toda sessão.

Caberá ao terapeuta checar os limites individuais de FC de cada indivíduo (que será obtido previamente segundo os limiares de FC extraídos do teste ergoespirométrico de cada participante).

Os sinais e sintomas clínicos serão monitorados ao longo de todas as sessões (Exemplo: cansaço excessivo, sudorese intensa, palidez, tontura, visão turva,palpitações, angina ou dor pré cordial) e caso o voluntário apresente qualquer alteração indicativa de riscos para o exercício o treinamento será suspenso e o voluntário encaminhado para o médico responsável para consulta médica.

Levando em consideração a complexidade do paciente com insuficiência cardíaca e a fim de atender toda e qualquer emergência ou intercorrência o Ginásio Terapêutico da Universidade de Brasília, local onde ocorrerá as intervenções de treinamento físico, estará equipado com todos os recursos necessários. Os recursos estarão disponíveis aos terapeutas para conter eventos como crises de hipoglicemia/hiperglicemia e hipertensão/hipotensão (estetoscópios, esfigmomanômetros, glicosímetro para checagem da glicemia, medicamentos de controle pressórico e glicêmico de emergência), e parada cardiorrespiratória (cilindro de oxigênio, máscaras para oxigenação, desfibrilador).

Cabe ressaltar que a fim de conter uma possível parada cardiorrespiratória, todos os terapeutas da pesquisa que estarão durante o treinamento físico dos pacientes, terão conhecimentos de primeiros socorros. Estes conhecimentos darão total autonomia aos profissionais de executarem com cuidado os procedimentos de reanimação cardiorrespiratória bem como ressuscitação, seguindo as recomendações do suporte básico de vida para adultos. Todos os terapeutas terão conhecimentos suficientes para reduzir o risco de complicações deste evento caso sua ocorrência.

Os indivíduos alocados no GC serão monitorados por telefone quanto aos sintomas e possíveis desconfortos sentidos com o treinamento uma vez por mês. Ainda, nos encontros presenciais que ocorrerão mensalmente, os indivíduos poderão tirar suas dúvidas com os profissionais de saúde, o que reduzirá os riscos ao longo de toda a participação na pesquisa. Caso tenha necessidade, serão consultados pela médica cardiologista da equipe em qualquer momento da pesquisa.

Entendemos que essas medidas serão suficientes para monitorização dos pacientes durante o treinamento e garantia de segurança a estes pacientes.

- 1. BENEFÍCIOS

Os participantes da pesquisa terão benefícios diretos, uma vez que receberão atendimento médico com realização de exames clínicos e físicos, além de atendimento fisioterapêutico de qualidade ao longo de todo protocolo de reabilitação cardiovascular. Os benefícios do protocolo de reabilitação acontecerão independente do grupo intervenção que o participante for sorteado (treinamento aeróbio intervalado de alta intensidade e treinamento resistido em circuito), uma vez que conforme introdução do presente projeto, ambas as modalidades são conhecidas na literatura pelos seus pontos positivos na reabilitação na população com IC. O protocolo de reabilitação auxiliará na redução de sintomas clínicos e funcionais provocados pela IC.

O GC que não realizará intervenção também terá benefícios uma vez que receberão atendimento médico com realização de exames clínicos e físicos. Além disso, uma vez por mês receberão palestras sobre como lidar com a IC. As palestras abordarão temas como nutrição, prática de exercícios, controle medicamentoso, aspectos psicológicos e serão realizadas por profissionais das respectivas áreas (nutricionista, fisioterapeuta, medica e psicóloga). Ainda, cabe ressaltar que após o período de pesquisa em que os indivíduos do GC precisam ficar sedentários, eles serão convidados a participar dos protocolos de exercício (sem finalidade de pesquisa), e receberão atendimento supervisionado pelos profissionais envolvidos no estudo.

Todos os pacientes, independente do grupo que forem alocados, receberão cópia de todos os exames realizados bem como *feedback* quanto a condição de saúde e orientação.

Entendemos que estes benefícios serão de suma importância para estes pacientes implicando na qualidade de vida dos mesmos.

1. **ANÁLISE ESTATÍSTICA E CÁLCULO AMOSTRAL**

A análise estatística dos dados será de forma descritiva para caracterização da amostra. O teste de normalidade dos dados será por Shapiro Wilk. Testes paramétricos e/ou não paramétricos serão aplicados e discriminados em tabelas segundo a normalidade. Testes comparativos paramétricos e/ou não paramétricos serão aplicados e discriminados entre os grupos em tabelas segundo a normalidade (ANOVA two way ou Kruskal Walllis). Possíveis correlações serão testadas usando a correlação de Spearman. Todas as análises serão realizadas utilizando o software estatístico SPSS versão 22.0 (SPSS, Inc. Chicago, IL, EUA), e o nível de significância adotado de 5%.

1. **DEESFECHOS**
   1. DESFECHO PRIMÁRIO
      - Verificação das variáveis de dilatação mediada pelo fluxo: diâmetro do vaso e velocidade do fluxo sanguíneo antes após taai e trc e grupo controle.
      - Análise das variáveis de cinética de oxigênio antes após taai e trc e grupo controle: consumo de oxigênio (o2), produção de dióxido de carbono (co2), ventilação minuto (e), volume corrente (vc), frequência respiratória (f), razão de troca respiratória (r), equivalentes ventilatórios para oxigênio (e/o2) e dióxido de carbono (e/ co2), tempo inspiratório (ti), tempo expiratório (te), e relação ti/ttot
      - Verificação da capacidade funcional pelo teste ergoespirométrico nas variáveis vo2 pico e ve/co2 antes após taai e trc e grupo controle.
   2. DESFECHO SECUNDÁRIO
      - Análise e comparação da modulação autonômica em pacientes com IC antes e após TAAI e TRC pelos índices lineares de variabilidade da frequência cardíaca.
      - Análise e comparação da composição corporal em pacientes com IC antes e após TAAI e TRC pela densitometria nos parâmetros % de gordura corporal, massa magra (kg) e massa adiposa(kg).
      - Caracterização da variação da espessura e ecointensidade muscular pela ultrassonografia em pacientes com IC antes e após TAAI e TRC.
      - Avaliação das respostas de qualidade de vida dos pacientes com IC pela classificação dos estados de funcionalidade e incapacidade (CIF) antes e após intervenções e grupo controle
      - Análise dos efeitos do TAAI e TRC na força de preensão palmar pelo handgrip expressos em quilogramas/força
      - Análise da capacidade físico funcional em pacientes com IC antes e após intervenções e grupo controle pela bateria performance física curta (short physical vattery performance).
      - Análise dos efeitos do TAAI e TRC na força muscular periférica por meio do teste de 1 repetição máxima (1RM)
      - Avaliação da força muscular isocinética e extração periférica de oxigênio
2. **REFERÊNCIAS BIBLIOGRÁFICAS**
3. Ponikowski P, Voors AA, Anker SD, Bueno H, Cleland JGF, Coats AJS, et al. 2016 ESC Guidelines for the diagnosis and treatment of acute and chronic heart failure The Task Force for the diagnosis and treatment of acute and chronic heart failure of the European Society of Cardiology ( ESC ) Developed with the special contribution. Eur J Heart Fail. 2016;18(8):891–

975.

1. Reibis R, Salzwedel A, Buhlert H, Wegscheider K, Eichler S, Völler H. Impact of training methods and patient characteristics on exercise capacity in patients in cardiovascular rehabilitation. Eur J Prev Cardiol [Internet]. 2016;23(5):452–9. Available from: <http://www.ncbi.nlm.nih.gov/pubmed/26285771>
2. Xie B, Yan X, Cai X, Li J. Effects of High-Intensity Interval Training on Aerobic Capacity in Cardiac Patients: A Systematic Review with Meta-Analysis. Biomed Res Int. 2017;2017.
3. Conraads VM, Pattyn N, De Maeyer C, Beckers PJ, Coeckelberghs E, Cornelissen VA, et al. Aerobic interval training and continuous training equally improve aerobic exercise capacity in patients with coronary artery disease: The SAINTEX-CAD study. Int J Cardiol [Internet]. 2015;179:203–10. Available from: <http://dx.doi.org/10.1016/j.ijcard.2014.10.155>
4. Ellingsen Ø, Halle M, Conraads V, Delagardelle C, Larsen A, Hole T, et al. High Intensity Interval Training in Heart Failure Patients with Reduced Ejection Fraction. Circulation. 2017;136(23):1–7.
5. Suchy C, Massen L, Rognmo O, Van Craenenbroeck EM, Beckers P, Kraigher-Krainer E, et al. Optimising exercise training in prevention and treatment of diastolic heart failure (OptimEx- CLIN): rationale and design of a prospective, randomised, controlled trial. Eur J Prev Cardiol. 2014 Nov;21(2 Suppl):18–25.
6. Meka N, Katragadda S, Cherian B, Arora RR. Review: Endurance exercise and resistance training in cardiovascular disease. Ther Adv Cardiovasc Dis [Internet]. 2008;2(2):115–21. Available from: <http://journals.sagepub.com/doi/10.1177/1753944708089701>
7. Polito MD, Farinatti PTV. Respostas de frequência cardíaca, pressão arterial e duplo-produto ao exercício contra-resistência: uma revisão da literatura. Rev Port Ciências do Despòorto. 2003;3(1):79–91.
8. Kelemen MH, Stewart KJ, Gillilan RE, Ewart CK, Valenti SA, Manley JD, et al. Circuit weight training in cardiac patients. J Am Coll Cardiol. 1986;7(1):38–42.
9. Hare DL, Ryan TM, Selig SE, Pellizzer a M, Wrigley T V, Krum H. Resistance exercise training increases muscle strength, endurance, and blood flow in patients with chronic heart failure. Am J Cardiol. 1999;83(12):1674–7, A7.
10. Williams AD, Carey MF, Selig S, Hayes A, Krum H, Patterson J, et al. Circuit Resistance Training in Chronic Heart Failure Improves Skeletal Muscle Mitochondrial ATP Production Rate-A Randomized Controlled Trial. J Card Fail. 2007;13(2):79–85.
11. Maupoint J, Besnier M, Gomez E, Bouhzam N, Henry JP, Boyer O, et al. Selective vascular endothelial protection reduces cardiac dysfunction in chronic heart failure. Circ Hear Fail. 2016;9(4).
12. Paulus WJ, Tschöpe C. A novel paradigm for heart failure with preserved ejection fraction: Comorbidities drive myocardial dysfunction and remodeling through coronary microvascular endothelial inflammation. J Am Coll Cardiol [Internet]. 2013;62(4):263–71. Available from: <http://dx.doi.org/10.1016/j.jacc.2013.02.092>
13. Sandri M, Viehmann M, Adams V, Rabald K, Mangner N, Höllriegel R, et al. Chronic heart failure and aging – effects of exercise training on endothelial function and mechanisms of endothelial regeneration: Results from the Leipzig Exercise Intervention in Chronic heart failure and Aging (LEICA) study. Eur J Prev Cardiol [Internet]. 2016;23(4):349–58. Available from: <http://journals.sagepub.com/doi/10.1177/2047487315588391>
14. Gutiérrez E, Flammer AJ, Lerman LO, Elízaga J, Lerman A, Francisco FA. Endothelial dysfunction over the course of coronary artery disease. Eur Heart J. 2013;34(41):3175–81.
15. Katz SD, Hryniewicz K, Hriljac I, Balidemaj K, Dimayuga C, Hudaihed A, et al. Vascular endothelial dysfunction and mortality risk in patients with chronic heart failure. Circulation. 2005 Jan;111(3):310–4.
16. Engelen M, Porszasz J, Riley M, Wasserman K, Maehara K, Barstow TJ. Effects of hypoxic hypoxia on O2 uptake and heart rate kinetics during heavy exercise. J Appl Physiol. 1996;81(6):2500–8.
17. Stirling JR, Zakynthinaki M. Counterpoint: The kinetics of oxygen uptake during muscular exercise do not manifest time-delayed phases. J Appl Physiol [Internet]. 2009;107(5):1665–7. Available from: <http://jap.physiology.org/cgi/doi/10.1152/japplphysiol.00158.2009a>
18. Poole DC, Richardson RS, Haykowsky MJ, Hirai DM, Musch TI. Exercise Limitations in Heart Failure with Reduced and Preserved Ejection Fraction. J Appl Physiol [Internet]. 2017;(October):jap.00747.2017. Available from: <http://jap.physiology.org/lookup/doi/10.1152/japplphysiol.00747.2017>
19. Thijssen DHJ, Black MA, Pyke KE, Padilla J, Atkinson G, Harris RA, et al. Assessment of flow-mediated dilation in humans: a methodological and physiological guideline. AJP Hear Circ Physiol [Internet]. 2011;300(1):H2–12. Available from: <http://ajpheart.physiology.org/cgi/doi/10.1152/ajpheart.00471.2010>
20. Restaino RM, Holwerda SW, Credeur DP, Fadel PJ, Padilla J. Impact of prolonged sitting on lower and upper limb micro- and macrovascular dilator function. Exp Physiol [Internet].

2015;100(7):829–38. Available from: <http://doi.wiley.com/10.1113/EP085238>

1. Boyle LJ, Credeur DP, Jenkins NT, Padilla J, Leidy HJ, Thyfault JP, et al. Impact of reduced daily physical activity on conduit artery flow-mediated dilation and circulating endothelial microparticles. J Appl Physiol [Internet]. 2013;115(10):1519–25. Available from: <http://jap.physiology.org/cgi/doi/10.1152/japplphysiol.00837.2013>
2. Padilla J, Sheldon RD, Sitar DM, Newcomer SC. Impact of acute exposure to increased hydrostatic pressure and reduced shear rate on conduit artery endothelial function: a limb- specific response. AJP Hear Circ Physiol [Internet]. 2009;297(3):H1103–8. Available from: <http://ajpheart.physiology.org/cgi/doi/10.1152/ajpheart.00167.2009>
3. Padilla J, Johnson BD, Newcomer SC, Wilhite DP, Mickleborough TD, Fly AD, et al. Adjusting flow-mediated dilation for shear stress stimulus allows demonstration of endothelial dysfunction in a population with moderate cardiovascular risk. J Vasc Res. 2009;46(6):592– 600.
4. Cornelis J, Beckers P, Taeymans J, Vrints C, Vissers D. Comparing exercise training modalities in heart failure: A systematic review and meta-analysis. Int J Cardiol [Internet]. 2016;221:867–

76. Available from: <http://dx.doi.org/10.1016/j.ijcard.2016.07.105>

1. de Rezende Barbosa MP da C, Silva NT da, de Azevedo FM, Pastre CM, Vanderlei LCM. Comparison of Polar?? RS800G3??? heart rate monitor with Polar?? S810i??? and electrocardiogram to obtain the series of RR intervals and analysis of heart rate variability at rest. Clin Physiol Funct Imaging. 2016;36(2):112–7.
2. Vanderlei LCM, Pastre CM, Hoshi RA, Carvalho TD De, Godoy MF De. Basic notions of heart rate variability and its clinical applicability. Rev Bras Cir Cardiovasc. 2009;24(2):205–17.
3. Dias de Carvalho T, Marcelo Pastre C, Claudino Rossi R, de Abreu LC, Valenti VE, Marques Vanderlei LC. Geometric index of heart rate variability in chronic obstructive pulmonary disease. Rev Port Pneumol (English Ed [Internet]. 2011;17(6):260–5. Available from: <http://www.sciencedirect.com/science/article/pii/S2173511511000467>
4. Tillquist M, Kutsogiannis DJ, Wischmeyer PE, Kummerlen C, Leung R, Stollery D, et al. Bedside Ultrasound Is a Practical and Reliable Measurement Tool for Assessing Quadriceps Muscle Layer Thickness. J Parenter Enter Nutr [Internet]. 2014;38(7):886–90. Available from: <http://journals.sagepub.com/doi/10.1177/0148607113501327>
5. Abràmofff MD, Magalhães PJ, Ram SJ. Image processing with ImageJ Part II. Biophotonics Int. 2005;11(7):36–43.
6. Sarwal A, Parry SM, Berry MJ, Hsu F-C, Lewis MT, Justus NW, et al. Interobserver Reliability of Quantitative Muscle Sonographic Analysis in the Critically Ill Population. J Ultrasound Med [Internet]. 2015;34(7):1191–200. Available from: <http://doi.wiley.com/10.7863/ultra.34.7.1191>
7. Pillen S, van Keimpema M, Nievelstein RAJ, Verrips A, van Kruijsbergen-Raijmann W, Zwarts MJ. Skeletal muscle ultrasonography: Visual versus quantitative evaluation. Ultrasound Med Biol. 2006;32(9):1315–21.
8. Martins EF, Mara S, Fracon JDF, Sá C De. a Rtigos O Riginais Experiência No Uso Combinado Das Classificações Internacionais Para Descrever Informações Em Saúde Experience in the Combined Utilization of the International Classifications To Describe Health Information. 2010;19–27.
9. American Society of Hand Therapists. Clinical assessment recommendations. 2nd ed. Chicago (401 N. Michigan Ave., Chicago IL 60611-4267) : The Society ©1992, editor. 1992.
10. Freire AN, Guerra RO, Alvarado B, Guralnik JM, Zunzunegui MV. Validity and Reliability of the Short Physical Performance Battery in Two Diverse Older Adult Populations in Quebec and Brazil. J Aging Health [Internet]. 2012;24(5):863–78. Available from: <http://journals.sagepub.com/doi/10.1177/0898264312438551>
11. Miller MR, Hankinson J, Brusasco V, Burgos F, Casaburi R, Coates A, Crapo R, Enright P, van der Grinten CP, Gustafsson P, Jensen R, Johnson DC, MacIntyre N, McKay R, Navajas D, Pedersen OF, Pellegrino R, Viegi G WJATF. Standardisation of spirometry. Eur Respir J. 2005;26(2):319-38.
12. Leong DP, Teo KK, Rangarajan S, Lopez-Jaramillo P, Avezum A Jr, Orlandini A, Seron P, Ahmed SH, Rosengren A, Kelishadi R, Rahman O, Swaminathan S, Iqbal R, Gupta R, Lear SA, Oguz A, Yusoff K, Zatonska K, Chifamba J, Igumbor E, Mohan V, Anjana RM, Gu H, Li YS. Prognostic value of grip strength: findings from the Prospective Urban Rural Epidemiology (PURE) study. Lancet. 2015;18(386(9990)):266–73.
13. Izawa KP, Watanabe S, Oka K, Hiraki K, Morio Y, Kasahara Y, Watanabe Y, Katata H, Osada N OK. Upper and Lower Extremity Muscle Strength Levels Associated With an Exercise Capacity of 5 Metabolic Equivalents in Male Patients With Heart Failure. J Cardiopulm Rehabil Prev. 2012;32(2):85–91.
14. Ponikowski P, Voors AA, Anker SD, Bueno H, Cleland JGF, Coats AJS, et al. 2016 ESC Guidelines for the diagnosis and treatment of acute and chronic heart failure. Eur Heart J. 2016;37(27):2129-2200m.
15. Chronic TTF for the diagnosis and treatment of acute and, (ESC) heart failure of the ES of C. 2016 ESC Guidelines for the diagnosis and treatment of acute and chronic heart failure. Eur Hear J (. 2016;37:2129–2200.
16. Nagueh SF, Smiseth OA, Appleton CP, Byrd BF, Dokainish H E, T et al. Recommendations for the evaluation of left ventricular diastolic function by echocardiography: na update from American Society of Echocardiography and the European Association ofn Cardiovascular Imaging. J Am Soc Echocardiogr. 2016;29(4):277–314.
17. Lang RM, Badano LP, Mor-Avi V, Afilalo J, Armstrong A, Ernande L et al. Recommendations for cardiac chambre quantification by echocardiography in adults: na update from American Society of Echocardiography and the European Association of Cardiovascular Imaging. J Am Soc Echocardiogr. 2015;28(1):1–39.
