# Supplementary material for: High-intensity interval training versus progressive high-intensity circuit resistance training on endothelial function and cardiorespiratory fitness in heart failure: A preliminary randomized controlled trial
Source: PLoS One. 2021 Oct 1;16(10):e0257607. doi: 10.1371/journal.pone.0257607 (PMC8486136; doi:10.1371/journal.pone.0257607)
Supplement: S3 File — (DOCX) [file pone.0257607.s004.docx]

Chronological dates of the study steps:

- March 21st, 2018 - approval on Brazilian Ethical Commite
- May 21st, 2018 - approval on Study Registration Platform – REBEC
- June 1st, 2018 - first date for enrollment in Brazil
- September 8th, 2019 - last date for enrollment in Brazil
- December 5th, 2020 - approval on Belgic Ethical Commite

This study did not started as a multicentric one, but along the PhD of the main researcher there was an opportunity to make it, stablishing a joint PhD partnership between Brazil and Belgium.

Changes were made in the initial protocol in order to adapt the trial to a multicentric one. The outcomes initially cited on Brazilian Ethical Committe “quality of life” and “oxygen extraction” were not included on Belgic ethical Commite due to technical limitations. In addition, the outcomes “autonomic modulation”, “muscle ultrasound” and “pulmonar function” were not included on this paper, but they were obtained and can be presented in a future pape or upon reasonable request.

Natália Turri-Silva

(on behalf of all coauthors)
