# Supplementary figures and images for: High-intensity interval training versus progressive high-intensity circuit resistance training on endothelial function and cardiorespiratory fitness in heart failure: A preliminary randomized controlled trial
Source: PLoS One. 2021 Oct 1;16(10):e0257607. doi: 10.1371/journal.pone.0257607 (PMC8486136; doi:10.1371/journal.pone.0257607)

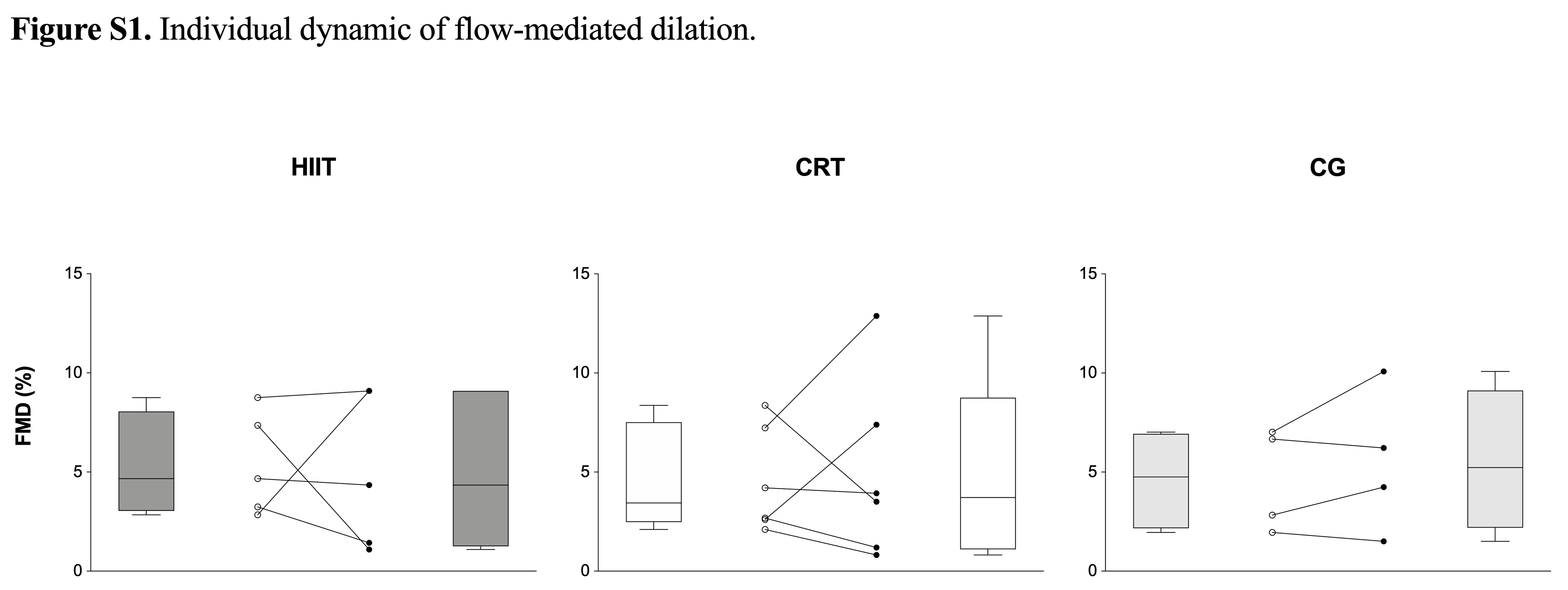

Supplement: S1 Fig — Flow-mediated dilation individual dynamics. HIIT, high-intensity interval training; CRT, circuit-resistance training; CG, control group. (TIF) [file pone.0257607.s005.tif]
