# Supplementary material for: High-intensity interval training versus progressive high-intensity circuit resistance training on endothelial function and cardiorespiratory fitness in heart failure: A preliminary randomized controlled trial
Source: PLoS One. 2021 Oct 1;16(10):e0257607. doi: 10.1371/journal.pone.0257607 (PMC8486136; doi:10.1371/journal.pone.0257607)
Supplement: S1 Data — DXA (dual x-ray absorptiometry); HIIT (high-intensity interval training); CRT (circuit-resistance training); CG (control group). Comparisons between groups were analyzed by the Two-way ANOVA (group*time interaction and group interaction). The baseline and post were analyzed by Kruskall-Wallis Test. The normality was analyzed by Kolmogorov-Smirnov test. Values are expressed as mean ± standard deviation (SD). A statistically significant difference was considered when there was a p value <0.05. Obs. Considering some patients made use of pacemakers, only 16 patients made biodex assessment. No differences among baseline parameters were found, neither differences related to the intervention. (DOCX) [file pone.0257607.s006.docx]

**Supporting information_tables**

| **DXA** | **HIIT** | | **CRT** | | **CG** | | **Within-group difference**  **(post minus pre)** | | | **Between-group difference** | | |
| --- | --- | --- | --- | --- | --- | --- | --- | --- | --- | --- | --- | --- |
|  | **Mean ± SD (95% CI)** | | **Mean ± SD (95% CI)** | | **Mean ± SD (95% CI)** | | **MD (95% CI)** | | | **MD (95% CI)** | | |
|  | **pre (n=4)** | **post (n=4)** | **pre (n=4)** | **post (n=4)** | **pre (n=7)** | **post (n=7)** | ∆ **HIIT** | ∆ **CRT** | ∆ **CG** | **HIIT vs. CRT** | **HIIT vs. CG** | **CRT vs. CG** |
| Total fat mass (%) | 36.9 ± 0.5  (36.0 to 37.7) | 37.1 ± 1.7  (34.4 to 39.9) | 40.2 ± 8.2  (27.2 to 53.1) | 39.9 ± 8.6  (22.2 to 53.5) | 33.9 ± 3.8  (30.4 to 37.4) | 33.2 ± 4.2  (29.3 to 37.1) | 0.3  (-1.9 to 2.4) | -0.3  (-2.5 to 1.9) | -0.7  (-2.4 to 0.9) | 3.0  (-6.9 to 12.9) | -3.5  (-12.2 to 5.3) | -6.5  (-15.2 to 2.3) |
| p value |  |  |  |  |  |  | >0.999 | >0.999 | 0.748 | >0.999 | 0.886 | 0.188 |
| Total lean mass (%) | 60.5 ± 0.6  (59.5 to 61.5) | 61.2 ± 1.5  (58.7 to 63.6) | 57.8 ± 8.1  (45.0 to 70.7) | 58.6 ± 7.8  (46.2 to 70.9) | 63.8 ± 3.9  (60.2 to 67.5) | 64.1 ± 3.6  (60.8 to 67.5) | 0.7  (-1.5 to 3.0) | 0.8  (-1.5 to 3.0) | 0.3  (-1.4 to 2.0) | 0.5  (-4.4 to 5.3) | 0.1  (-5.3 to 5.5) | -0.3  (-5.5 to 4.9) |
| p value |  |  |  |  |  |  | 0.783 | 0.755 | 0.951 | >0.999 | >0.999 | >0.999 |

**Table SS1.**

**Legend:** Values are expressed as mean ± standard deviation (SD), mean difference (MD) and 95% CI (confidence interval). Comparisons between groups were analyzed by the mixed ANOVA with Bonferroni post-test (within and between) for all parameters. *****p ≤0.05. DXA, dual x-ray absorptiometry; HIIT, high-intensity interval training; CRT, circuit-resistance training; CG, control group; ∆, post - pre values. **Obs.** Considering some patients made use of pacemakers, only 15 patients made DXA assessment. No differences among baseline parameters were found, neither differences related to the intervention.

**Table SS2.**

| **FMD** | **HIIT** | | **CRT** | | **CG** | | **Within-group difference**  **(post minus pre)** | | | **Between-group difference** | | |
| --- | --- | --- | --- | --- | --- | --- | --- | --- | --- | --- | --- | --- |
|  | **Mean ± SD (95% CI)** | | **Mean ± SD (95% CI)** | | **Mean ± SD (95% CI)** | | **MD (95% CI)** | | | **MD (95% CI)** | | |
|  | **pre (n=5)** | **post (n=5)** | **pre (n=6)** | **post (n=6)** | **pre (n=4)** | **post (n=4)** | ∆ **HIIT** | ∆ **CRT** | ∆ **CG** | **HIIT vs. CRT** | **HIIT vs. CG** | **CRT vs. CG** |
| Rest diameter (mm) | 4.54 ± 1.09  (3.19 to 5.88) | 4.54 ± 0.92  (3.39 to 5.68) | 4.38 ± 0.58  (3.77 to 4.98) | 4.52 ± 0.61  (3.88 to 5.17) | 4.36 ± 1.17  (2.94 to 6.23) | 4.35 ± 0.51  (3.55 to 5.16) | 0.00  (-0.67 to 0.67) | 0.15  (-0.47 to 0.76) | -0.01  (-0.76 to 0.74) | 0.09  (-1.24 to 1.41) | 0.18  (-1.29 to 1.65) | 0.09  (-1.32 to 1.50) |
| p value |  |  |  |  |  |  | >0.999 | >0.999 | >0.999 | >0.999 | >0.999 | >0.999 |
| Peak diameter (mm) | 4.77 ± 1.10  (3.41 to 6.13) | 4.75 ± 0.88  (3.66 to 5.84) | 4.57 ± 0.59  (3.95 to 5.19) | 4.74 ± 0.57  (4.14 to 5.33) | 4.54 ± 1.12  (2.77 to 6.31) | 4.58 ± 0.39  (3.96 to 5.21) | -0.02  (-0.72 to 0.68) | 0.17  (-0.47 to 0.80) | 0.04  (-0.74 to 0.82) | 0.10  (-1.16 to 1.37) | 0.19  (-1.21 to 1.60) | 0.09  (-1.26 to 1.44) |
| p value |  |  |  |  |  |  | >0.999 | >0.999 | >0.999 | >0.999 | >0.999 | >0.999 |
| FMD (%) | 5.37 ± 2.59  (2.16 to 8.59) | 5.01 ± 3.93  (0.13 to 9.90) | 4.54 ± 2.65  (1.76 to 7.32) | 4.96 ± 4.54  (0.19 to 9.72) | 4.62 ± 2.60  (0.48 to 8.76) | 5.51 ± 3.61  (-0.22 to 11.25) | -0.36  (-5.05 to 4.33) | 0.42  (-3.86 to 4.70) | 0.90  (-4.35 to 6.14) | 0.45  (-4.42 to 5.31) | 0.13  (-5.27 to 5.52) | -0.32  (-5.51 to 4.87) |
| p value |  |  |  |  |  |  | >0.999 | >0.999 | >0.999 | >0.999 | >0.999 | >0.999 |

**Legend:** Values are expressed as mean ± standard deviation (SD), mean difference (MD) and 95% CI (confidence interval). Comparisons between groups were analyzed by the mixed ANOVA with Bonferroni post-test (within and between) for all parameters. *****p ≤0.05. HIIT, high-intensity interval training; CRT, circuit-resistance training; CG, control group; FMD, flow-mediated dilatation; mm, millimeter; ∆, post - pre values.

**Table SS3.**

| **CPET** | **HIIT** | | **CRT** | | **CG** | | **Within-group difference**  **(post minus pre)** | | | **Between-group difference** | | |
| --- | --- | --- | --- | --- | --- | --- | --- | --- | --- | --- | --- | --- |
|  | **Mean ± SD (95% CI)** | | **Mean ± SD (95% CI)** | | **Mean ± SD (95% CI)** | | **MD (95% CI)** | | | **MD (95% CI)** | | |
|  | **pre (n=8)** | **post (n=8)** | **pre (n=6)** | **post (n=6)** | **pre (n=8)** | **post (n=8)** | ∆ **HIIT** | ∆ **CRT** | ∆ **CG** | **HIIT vs. CRT** | **HIIT vs. CG** | **CRT vs. CG** |
| Time Exercise (min) | 9.2 ± 2.6  (7.0 to 11.4) | 10.6 ± 2.4  (8.5 to 12.6) | 10.0 ± 2.8  (7.0 to 12.9) | 10.8 ± 2.1  (8.6 to 13.0) | 10.2 ± 2.6  (8.1 to 12.4) | 10.4 ± 2.6  (8.2 to 12.5) | -1.3  (-0.3 to 3.0) | 0.8  (-1.1 to 2.7) | 0.1  (-1.5 to 1.8) | 0.5 (-2.9 to 3.9) | 0.4 (-2.7 to 3.5) | -0.1 (-3.5 to 3.3) |
| p value |  |  |  |  |  |  | 0.152 | 0.837 | >0.999 | >0.999 | >0.999 | >0.999 |
| Cycling peak power output (watt) | **96.8 ± 26.3**  **(74.8 to 118.7)** | **114.9 ± 34.7 (85.9 to 143.9)** | 104.2 ± 27.7 (75.1 to 133.3) | 112.0 ± 34.1 (76.2 to 147.8) | 123.3 ± 35.2 (93.8 to 152.7) | 123.6 ± 37.5 (99.3 to 155.0) | **18.1**  **(4.4 to 31.9)*** | 7.8  (-8.1 to 23.7) | 0.4 (-13.4 to 14.1) | 2.3 (-43.4 to 47.9) | 17.6 (-24.6 to 59.9) | 15.4 (-30.3 to 61.0) |
| p value |  |  |  |  |  |  | **0.008*** | 0.633 | >0.999 | >0.999 | >0.861 | >0.999 |
| RER peak | 1.2 ± 0.1  (1.1 to 1.3) | 1.2 ± 0.1  (1.1 to 1.2) | 1.2 ± 0.1  (1.0 to 1.3) | 1.1 ± 0.1  (1.0 to 1.2) | 1.2 ± 0.1  (1.1 to 1.3) | 1.2 ± 0.1  (1.2 to 1.3) | -0.1  (-0.2 to 0.1) | -0.1  (-0.2 to 0.1) | 0.0  (-0.2 to 0.1) | -0.1 (-0.2 to 0.1) | 0.0 (-0.1 to 0.1) | 0.1 (-0.1 to 0.2) |
| p value |  |  |  |  |  |  | 0.552 | 0.750 | >0.999 | 0.799 | >0.861 | 0.359 |
| HR VT1 (bpm) | 100.3 ± 13.0 (89.4 to 111.1) | 100.4 ± 11.0 (91.2 to 109.6) | 109.2 ± 12.8 (93.4 to 125.0) | 107.6 ± 8.5 (97.1 to 118.1) | 101.4 ± 20.5 (84.3 to 118.5) | 96.3 ± 15.3 (83.5 to 109.0) | 0.1  (-13.5 to 13.8) | -1.6 (-18.8 to 15.6) | -5.1 (-18.8 to 8.5) | 8.1 (-10.7 to 26.9) | -1.5 (-18.0 to 15.0) | -9.6 (-28.4 to 9.2) |
| p value |  |  |  |  |  |  | >0.999 | >0.999 | >0.999 | 0.813 | >0.999 | 0.585 |
| HR VT2 (bpm) | 113.1 ± 19.0  (97.3 to 129.0) | 122.4 ± 13.2 (110.2 to 134.7) | 119.3 ± 16.4 (102.1 to 136.6) | 123.2 ± 5.6 (116.1 to 130.1) | 122.8 ± 20.8 (105.3 to 140.2) | 116.4 ± 12.7 (105.8 to 127.0) | 7.6 (-7.8 to 23.0) | 2.0 (-16.1 to 20.2) | -6.4 (-21.0 to 8.3) | 3.4 (-17.0 to 23.8) | 2.6 (-16.0 to 21.3) | -0.8 (-21.0 to 19.4) |
| p value |  |  |  |  |  |  | 0.620 | >0.999 | 0.791 | >0.999 | >0.999 | >0.999 |
| HR peak (bpm) | 125.0 ± 24.7 (104.3 to 145.7) | 129.4 ± 20.5 (112.2 to 146.6) | 122.7 ± 16.9 (104.9 to 140.4) | 129.2 ± 9.9 (118.8 to 139.5) | 142.0 ± 17.9 (127.0 to 157.0) | 139.9 ± 14.5 (128.8 to 152.0) | 4.4 (-13.4 to 22.1) | 6.5 (-14.0 to 27.0) | -2.1 (-19.9 to 15.6) | -1.3 (-23.5 to 21.0) | 13.8 (-6.9 to 34.4) | 15.0 (-7.3 to 37.3) |
| p value |  |  |  |  |  |  | >0.999 | >0.999 | >0.999 | >0.999 | 0.288 | 0.278 |
| V̇O_2_ VT1 (ml.kg.min) | 11.0 ± 2.2  (9.2 to 12.8) | 13.8 ± 1.9  (12.2 to 15.4) | 13.8 ± 3.2  (10.4 to 17.2) | 15.0 ± 2.9  (12.0 to 18.1) | 13.5 ± 6.4 (8.1 to 18.9) | 13.1 ± 3.0  (10.6 to 15.6) | 2.7  (-1.6 to 7.1) | 1.3  (-3.8 to 6.3) | -0.4  (-4.8 to 4.0) | 2.0 (-2.0 to 6.0) | 0.9 (-2.8 to 4.6) | -1.1 (-5.1 to 2.9) |
| p value |  |  |  |  |  |  | 0.351 | >0.999 | >0.999 | 0.600 | >0.999 | >0.999 |
| V̇O_2_ VT2 (ml.kg.min) | **15.1 ± 2.5**  **(13.1 to 17.1)** | **18.8 ± 3.4**  **(15.6 to 21.9)** | 17.9 ± 5.0  (12.7 to 23.2) | 19.2 ± 2.6  (16.5 to 21.9) | 17.4 ± 2.0  (15.7 to 19.0) | 16.2 ± 2.3  (14.2 to 18.1) | **3.7**  **(1.0 to 6.3)*** | 1.3  (-1.6 to 4.2) | -1.2  (-3.7 to 1.3) | 1.6 (-2.2 to 5.4) | -0.2 (-3.7 to 3.1) | -1.8 (-5.6 to 2.0) |
| p value |  |  |  |  |  |  | **0.006*** | 0.751 | 0.682 | 0.832 | >0.999 | 0.666 |
| V̇O_2_ peak (ml.kg.min) | **17.5 ± 4.2**  **(14.0 to 21.0)** | **19.6 ± 4.9**  **(15.6 to 23.7)** | **16.9 ± 2.5**  **(14.3 to 19.5)** | **19.9 ± 3.4**  **(16.4 to 23.5)** | 20.2 ± 3.3  (17.4 to 23.0) | 20.1 ± 4.2  (16.6 to 24.0) | **2.2**  **(0.2 to 4.1)*** | **3.1**  **(0.8 to 5.3)*** | -0.1  (-2.0 to 1.9) | -0.1 (-5.5 to 5.2) | 1.6 (-3.4 to 6.5) | 1.7 (-3.6 to 7.1) |
| p value |  |  |  |  |  |  | **0.026*** | **0.006*** | >0.999 | >0.999 | >0.999 | >0.999 |
| V̇O_2_ peak (ml.min) | 1437.3 ± 411.3  (1093.4 to 1781.1) | 1563.4 ± 445.7  (1190.7 to 1936.0) | 1565.7 ± 365.6  (1182.0 to 1949.3) | 1749.0 ± 421.7  (1306.4 to 2191.6) | 1605.0 ± 458.4  (1221.8 to 1988.2) | 1586.4 ± 474.3  (1189.9 to 1982.9) | 126.1 (-37.4 to 289.6) | 183.3 (-5.5 to 372.2) | -18.6 (-182.1 to 144.9) | 157.0  (-446.3 to 760.4) | 95.4  (-463.2 to 654.0) | -61.7  (-665.0 to 541.7) |
| p value |  |  |  |  |  |  | 0.172 | 0.059 | >0.999 | >0.999 | >0.999 | >0.999 |
| Slope VE/VCO_2_ | 30.1 ± 5.8  (25.3 to 35.0) | 31.9 ± 5.8  (27.1 to 36.7) | 27.1 ± 5.0  (21.8 to 32.4) | 30.4 ± 7.2  (22.9 to 38.0) | **28.7 ± 7.1**  (22.8 to 34.6) | **32.9 ± 6.7**  (27.3 to 38.4) | 1.8  (-1.6 to 5.1) | 3.3  (-0.6 to 7.2) | **4.2**  **(0.8 to 7.5)*** | 2.3 (-6.3 to 10.8) | 0.2 (-7.7 to 8.2) | -2.0 (-10.6 to 6.6) |
| p value |  |  |  |  |  |  | 0.560 | 0.118 | **0.013*** | >0.999 | >0.999 | >0.999 |
| METs max | **5.0 ± 1.2**  **(4.0 to 6.0)** | **5.6 ± 1.4**  **(4.4 to 6.8)** | **4.8 ± 0.7**  **(4.1 to 5.6)** | **5.7 ± 1.0**  **(4.7 to 6.7)** | 5.8 ± 1.0  (5.0 to 6.6) | 5.8 ± 1.2  (4.8 to 6.7) | **0.6**  **(0.0 to 1.2)*** | **0.9**  **(0.2 to 1.5)*** | 0.0  (-0.6 to 0.5) | 0.0 (-1.6 to 1.5) | 0.5 (-1.0 to 1.9) | -0.5 (-1.1 to 2.0) |
| p value |  |  |  |  |  |  | **0.033*** | **0.006*** | >0.999 | >0.999 | >0.999 | >0.999 |

**Legend:** Values are expressed as mean ± standard deviation (SD), mean difference (MD) and 95% CI (confidence interval). Comparisons between groups were analyzed by the mixed ANOVA with Bonferroni post-test (within and between) for all parameters. *****p ≤0.05. HIIT, high-intensity interval training; CRT, circuit-resistance training; CG, control group; CPET, cardiopulmonary exercise testing; sec, seconds; RER, respiratory exchange ratio; HR, heart rate; VT1, first ventilatory threshold; VT2, second ventilatory threshold; V̇O_2_, oxygen uptake; ml, millimeter; kg, kilogram; min, minute; METs max, maximal metabolic equivalent

**Table SS4.**

| **BIODEX** | **HIIT** | | **CRT** | | **CG** | | **Within-group difference**  **(post minus pre)** | | | **Between-group difference** | | |
| --- | --- | --- | --- | --- | --- | --- | --- | --- | --- | --- | --- | --- |
|  | **Mean ± SD (95% CI)** | | **Mean ± SD (95% CI)** | | **Mean ± SD (95% CI)** | | **MD (95% CI)** | | | **MD (95% CI)** | | |
|  | **pre (n=7)** | **post (n=7)** | **pre (n=5)** | **post (n=5)** | **pre (n=6)** | **post (n=6)** | ∆ **HIIT** | ∆ **CRT** | ∆ **CG** | **HIIT vs. CRT** | **HIIT vs. CG** | **CRT vs. CG** |
| Isokinetic torque peak (Nm) | 101.4 ± 37.2  (66.9 to 135.8) | 110.2 ± 41.6  (71.8 to 148.7) | 112.9 ± 47.4  (54.1 to 171.8) | 118.5 ± 46.1  (61.3 to 175.6) | 116.7 ± 41.4  (73.3 to 160.1) | 118.3 ± 40.1  (76.1 to 160.4) | 8.9  (-0.2 to 18.0) | 5.5  (-5.2 to 16.3) | 1.5  (-8.3 to 11.4) | 9.9 (-55.9 to 75.7) | 11.7 (-50.8 to 74.2) | 1.8 (-66.2 to 69.8) |
| p value |  |  |  |  |  |  | 0.057 | 0.557 | >0.999 | >0.999 | >0.999 | >0.999 |
| Isokinetic torque peak / Body weight (%) | **117.3 ± 28.8**  **(90.7 to 144.0)** | **129.4 ± 32.3**  **(99.6 to 159.3)** | 122.6 ± 40.5 (72.4 to 172.9) | 129.3 ± 37.3  (83.0 to 175.6) | 146.0 ± 29.1  (115.4 to 176.6) | 151.9 ± 36.7  (113.4 to 190.4) | **12.1**  **(1.8 to 22.4)*** | 6.7  (-5.5 to 18.8) | 5.9  (-5.2 to 17.0) | 2.6 (-50.2 to 55.4) | 25.6 (-24.6 to 75.7) | 23.0 (-31.6 to 77.5) |
| p value |  |  |  |  |  |  | **0.019*** | 0.479 | 0.514 | >0.999 | 0.569 | 0.824 |
| Total Work (J) | 1742.5 ± 590.5  (1196.3 to 2288.6) | 1902.1 ± 610.2  (1337.8 to 2466.5) | 2016.3 ± 733.7  (1105.0 to 2927.5) | 1981.3 ± 1181.1  (514.8 to 3447.9) | 2060.5 ± 664.4  (1363.3 to 2757.7) | 2117.9 ± 731.8  (1350.0 to 2885.8) | 159.7 (-224.7 to 544.1) | -34.9 (-489.8 to 419.9) | 57.4 (-357.8 to 472.6) | 273.8  (-1358.7 to 811.1) | -318.1  (-1348.8 to 712.8) | -44.3  (-1166.2 to 1077.7) |
| p value |  |  |  |  |  |  | 0.842 | >0.999 | >0.999 | 0.809 | 0.730 | 0.995 |
| Work / Body weight (%) | 132.3 ± 32.5  (102.3 to 162.4) | 148.5 ± 39.7  (111.8 to 185.2) | 139.6 ± 39.1  (91.0 to 188.1) | 144.7 ± 38.5  (97.0 to 192.5) | 166.8 ± 31.5  (133.7 to 199.9) | 176.9 ± 40.0  (134.9 to 218.8) | 16.2  (-3.4 to 35.9) | 5.2  (-18.1 to 28.4) | 10.1  (-11.2 to 31.3) | 1.7 (-54.4 to 57.8) | 31.4  (-21.9 to 87.7) | 29.7  (-28.4 to 87.7) |
| p value |  |  |  |  |  |  | 0.126 | >0.999 | 0.663 | >0.999 | 0.401 | 0.566 |
| Total Work Max Repetition (J) | 115.1 ± 45.8  (72.4 to 157.4) | 124.6 ± 42.1  (85.7 to 163.5) | 127.4 ± 45.2  (71.3 to 183.5) | 132.7 ± 49.9  (70.6 to 194.6) | 132.9 ± 42.6  (88.3 to 177.6) | 138.0 ± 45.2  (90.6 to 185.4) | 9.5  (-6.8 to 25.9) | 5.5  (-14.1 to 24.6) | 5.1  (-12.6 to22.7) | 10.2 (-59.5 to 80.0) | 15.6 (-50.6 to 81.9) | 5.4 (-66.7 to 77.5) |
| p value |  |  |  |  |  |  | 0.412 | >0.999 | >0.999 | >0.999 | >0.999 | >0.999 |
| Work Fatigue (%) | 35.3 ± 14.3  (26.7 to 43.9) | 41.3 ± 4.2  (38.8 to 43.7) | 39.2 ± 6.8  (34.3 to 44.1) | 35.7 ± 11.6  (23.5 to 44.0) | 39.2 ± 12.7  (25.8 to 52.5) | \| 43.4 ± 4.9  (38.3 to 48.5) \| \| --- \| | 8.1  (-4.3 to 20.5) | -3.1  (-17.7 to 11.6) | 4.2  (-9.2 to 17.6) | -0.1 (-14.2 to 14.0) | 4.1 (-9.4 to 17.5) | 4.2 (-10.5 to 18.8) |
| p value |  |  |  |  |  |  | 0.295 | >0.999 | >0.999 | >0.999 | >0.999 | >0.999 |
| Average power (Watt) | **141.1 ± 50.8**  **(94.2 to 188.1)** | **161.9 ± 63.2**  **(103.4 to 220.4)** | 175.2 ± 77.4  (79.1 to 271.4) | 191.3 ± 75.7  (97.3 to 285.2) | 167.6 ± 59.7  (105.0 to 230.2) | \| 169.0 ± 60.5  (105.5 to 232.6) \| \| --- \| | **20.8**  **(4.7 to 36.8)*** | 16.0  (-2.9 to 35.0) | 1.4 (-15.9 to 18.7) | 31.7 (-68.3 to 131.8) | 16.8 (-78.2 to 111.9) | -14.9  (-118.3 to 88.5) |
| p value |  |  |  |  |  |  | 0.010* | 0.114 | >0.999 | >0.999 | >0.999 | >0.999 |

**Legend:** Values are expressed as mean ± standard deviation (SD), mean difference (MD) and 95% CI (confidence interval). Comparisons between groups were analyzed by the mixed ANOVA with Bonferroni post-test (within and between) for all parameters. *****p ≤0.05. HIIT, high-intensity interval training; CRT, circuit-resistance training; CG, control group; Nm, newton-meter; J, Joules; Max, maximum.

**Table SS5**.

| **SPPB** | **HIIT** | | **CRT** | | **CG** | | **Within-group difference**  **(post minus pre)** | | | **Between-group difference** | | |
| --- | --- | --- | --- | --- | --- | --- | --- | --- | --- | --- | --- | --- |
|  | **pre (n=8)** | **post (n=8)** | **pre (n=5)** | **post (n=5)** | **pre (n=6)** | **post (n=6)** | ∆ **HIIT**  **MD (95% CI)** | ∆ **CRT**  **MD (95% CI)** | ∆ **CG**  **MD (95% CI)** | **HIIT vs. CRT** | **HIIT vs. CG** | **CTR vs. CG** |
| **Balance test** |  |  |  |  |  |  |  |  |  |  |  |  |
| Score  [Q1-Q3] | 4.0  [2.5-4.0] | 4.0  [4.0-4.0] | 4.0  [3.0-4.0] | 4.0  [4.0-4.0] | 4.0  [4.0-4.0] | 4.0  [4.0-4.0] | - | - | - | **0.046*** | | |
| **Gait test** |  |  |  |  |  |  |  |  |  |  |  |  |
| Score  [Q1-Q3] | 3.5  [3.0-4.0] | 3.5  [3.0-4.0] | 3.0  [3.0-4.0] | 4.0  [3.0-4.0] | 3.0  [3.0-4.0] | 4.0  [3.0-4.0] | - | - | - | 0.739 | | |
| Seconds  (95% CI) | 3.6 ± 0.6  (3.1 to 4.1) | 3.7 ± 0.9  (2.9 to 4.4) | 3.5 ± 0.6  (2.8 to 4.2) | 3.4 ± 0.6  (2.7 to 4.1) | 3.6 ± 0.7  (2.7 to 4.5) | 3.4 ± 0.7  (2.6 to 4.2) | 0.0  (-0.8 to 0.8) | -0.1  (-1.1 to 0.9) | -0.2  (-1.2 to 0.8) | -0.2  (-1.1 to 0.6) | -0.2  (-1.0 to 0.7) | 0.0  (-0.9 to 1.0) |
| p value |  |  |  |  |  |  | <0.999 | <0.999 | <0.999 | <0.999 | <0.999 | <0.999 |
| **Chair stand test** |  |  |  |  |  |  |  |  |  |  |  |  |
| Score  [Q1-Q3] | 2.5  [2.0-3.0] | 3.0  [2.3-3.8] | 2.0  [1.0-3.5] | 3.0  [3.0-4.0] | 3.0  [2.0-3.3] | 3.0  [2.5-4.0] | - | - | - | 0.083 | | |
| Seconds  (95% CI) | 13.5 ± 2.0  (11.8 to 15.1 | 12.8 ± 2.4  (10.8 to 14.8) | **14.3 ± 2.9**  **(10.7 to 18.0)** | **11.0 ± 1.4**  **(9.3 to 12.7)** | 12.9 ± 1.6  (11.0 to 14.9) | 12.6 ± 2.1  (10.0 to 15.3) | -0.6  (-3.1 to 1.9) | **-3.3**  **(-6.5 to -0.2)*** | -0.3  (-3.4 to 2.9) | -0.5  (-3.1 to 2.1) | -0.4  (-3.0 to 2.2) | 0.1  (-2.8 to 3.0) |
| p value |  |  |  |  |  |  | <0.999 | **0.037*** | <0.999 | <0.999 | <0.999 | <0.999 |
| **Total SPPB** |  |  |  |  |  |  |  |  |  |  |  |  |
| Score  [Q1-Q3] | 10.0  [8.3;10.8] | 11.0  [9.3;11.0] | 10.0  [8.0;10.0] | 11.0  [10.0;12.0] | 10.0  [9.5;11.0] | 11.0  [10.0;11.5] | - | - | **-** | **0.008*** | | |

**Legend:** SPPB, short physical performance battery; HIIT, high-intensity interval training; CRT, circuit-resistance training; CG, control group. Values are expressed as median and interquatile range (Q1-Q3) for scores and mean ± standard deviation (SD), mean difference (MD) and 95% CI (confidence interval) for seconds. Comparisons between groups were analyzed by mixed ANOVA for parametric distribution, while Friedman two-way test was applied for the difference among groups (HIIT, CRT and CG) which does not allow the comparison between groups pairwise. *****p ≤0.05
