# Supplementary material for: High-intensity interval training versus progressive high-intensity circuit resistance training on endothelial function and cardiorespiratory fitness in heart failure: A preliminary randomized controlled trial
Source: PLoS One. 2021 Oct 1;16(10):e0257607. doi: 10.1371/journal.pone.0257607 (PMC8486136; doi:10.1371/journal.pone.0257607)
Supplement: S1 List — (DOCX) [file pone.0257607.s007.docx]

**Supplier’s list**

^a^ Heparinized (sodium) tubes - Vacuette^®^ from Greiner Bio-One, Kremsmünster, Áustria

^b^ Cobas h232 - Roche Diagnostics, Basel, Switzerland

^c^ Ultrasound for echocardiography - HDI 5000 2-4 MHZ, Philips ATL, Bothell, WA

^d^ Spirometry - MicroLab ML3500MK8, CareFusion, EUA

^e^ High-resolution Doppler duplex ultrasound - HD11.XZ, Phillips, Barueri, SP, Brazil

^f^ Edge-detection software - Cardiovascular Suite, Quipu, Pisa, Italy

^g^ Cycle ergometer - Corival, LODE BV Medical Technology Groningen, Netherlands

^h^ Breath-by-breath gas analyzer - CPET, Cosmed, Rome, Italy

^i^ Electrocardiogram - Quark T12x, Cosmed, Rome, Italy

^j^ Isokinetic dynamometer - Biodex System 3 PRO, Medical Inc., New York, EUA

^k^ Dual x-ray absorptiometry scan - DXA - Lunar Prodigy Bone Densitometers, GE Healthcare, USA

^l^ Polar® - RS800, Polar Pro Trainer, Kempele, Finland

^m^ Resistive stations - EN-Dynamic, Enraf-Nonius, Rotterdam, the Netherlands

^n^ Treadmill - Gait Trainer, Biodex Medical Systems, Inc., New York, EUA

^o^ Ergometric bicycle - BioStep™ Semi-Recumbent Elliptical, Biodex Medical Systems, Inc., New York, EUA

^p^ G*Power Software 3 - 3.1.9.6 version, Heinrich Heine Universität Düsseldorf, Germany

^q^ Statistical software SPSS version 22.0 - SPSS, Inc. Chicago, IL, USA

^r^ GraphPad Prism - 8.4.0 version, California, San Diego
